# Reactivity of hypervalent iodine(III) reagents bearing a benzylamine with sulfenate salts

**DOI:** 10.3762/bjoc.20.272

**Published:** 2024-12-19

**Authors:** Beatriz Dedeiras, Catarina S Caldeira, José C Cunha, Clara S B Gomes, M Manuel B Marques

**Affiliations:** 1 LAQV-REQUIMTE, Department of Chemistry, NOVA School of Science and Technology, NOVA FCT , 2829-516 Caparica, Portugal

**Keywords:** electrophilic amination, hypervalent iodine reagents, sulfinamide, sulfonamide

## Abstract

The reactivity of our recently disclosed hypervalent iodine reagents (HIRs) bearing a benzylamine with in situ-generated sulfenate salts was investigated. Under the studied conditions sulfonamides have been obtained in up to 52% yield. This reaction has been extended to a variety of HIRs and sulfenate salts to explore the different reactivity of these new reagents. A plausible mechanism is proposed, suggesting a possible radical pathway.

## Introduction

Iodine(III) compounds, known as λ^3^-iodanes, have been extensively applied in organic synthesis. Although initially used as strong oxidizing agents [1], during the last decades HIRs have been investigated as group-transfer reagents, useful in several bond-forming reactions, such as in C–C, C–N, and C–O [2–5].

The benziodoxol(on)e family, cyclic iodine(III) reagents, stands out for their thermal stability and reactivity, yielding numerous derivatives with wide applications [2–4,6–8]. Their enhanced stability, compared to other HIRs, is due to: (i) the molecular geometry, which allows better overlap between the non-bonding electrons of the central iodine atom and the π-orbitals of the aromatic ring [2,9]; (ii) the incorporation of the iodine atom in the 5-membered heterocycle [10]; and (iii) the *trans* effect, due to the interaction of the hypervalent bonds established by the axial ligands, where iodine orbitals are shared with both heteroatoms [11]. As a result, benziodoxol(on)es have found application in electrophilic transfer reactions, with emphasis on umpolung reactivity of usually nucleophilic functional groups. Thus constituting a powerful synthetic tool, opening room for new synthetic disconnections [10].

**Figure 1:**
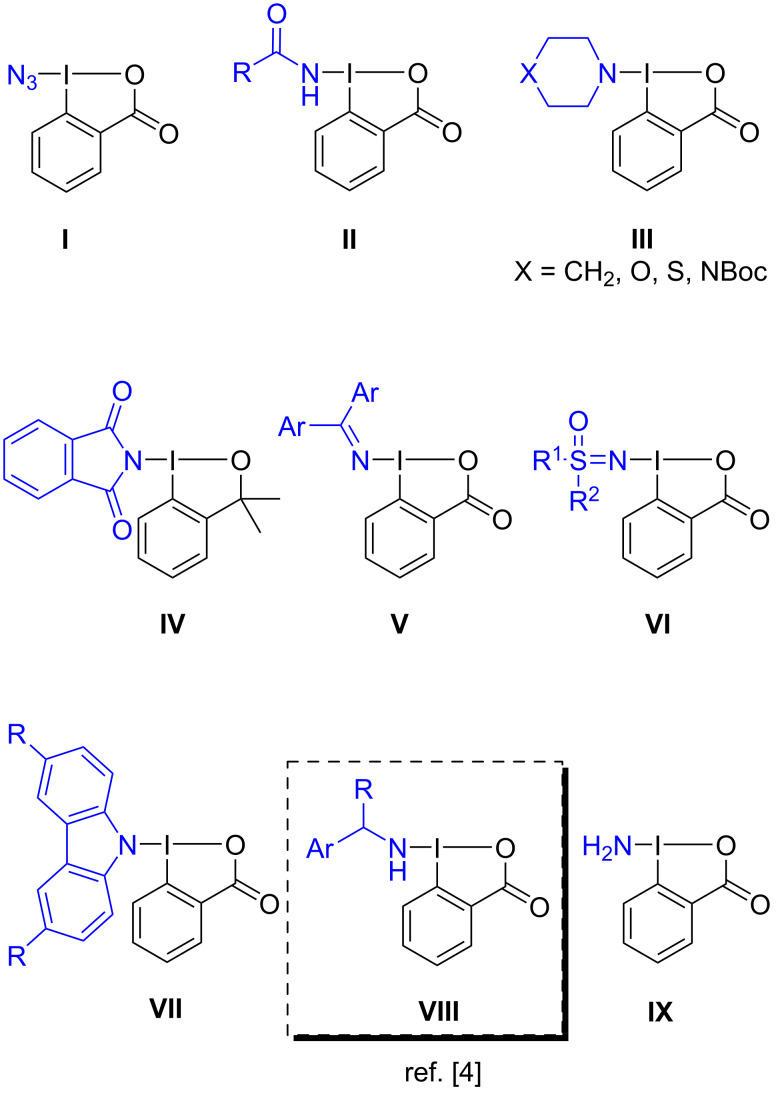
Examples of cyclic HIRs with a nitrogen-based group transfer [4,10,13–20].

Within the benziodoxol(on)e class, a range of HIRs featuring nitrogen-containing groups have been reported [12]. These reagents have proven effective in delivering azides (**I**) [13], amides (**II**) [14], aliphatic cyclic amines (**III**) [15], phthalimidates (**IV**) [16], imines (**V**) [17], sulfoximides (**VI**) [18], carbazoles (**VII**) [19], secondary (**VIII**) [4] and primary (**IX**) [20] amines (Figure 1).

**Scheme 1:**
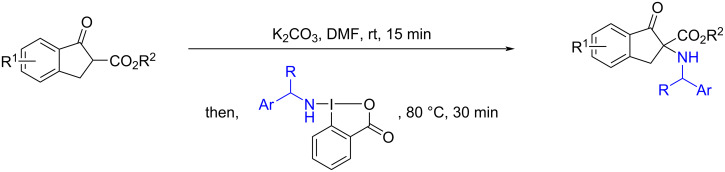
Electrophilic α‑amination of indanone-based β-ketoesters [4].

The first report from Zhdankin and co-workers in 1994, described the preparation of azidobenziodoxolone, ABX (**I**), a reagent widely used in oxidative azide transfer reactions [21]. Years later, Zhdankin’s group also reported the synthesis of amidobenziodoxolone (**II**) [14]. Other examples of N-containing benziodoxol(on)es can be found in the literature, including reagents featuring cyclic aliphatic amine moieties (**III**) [15], phthalimidates (**IV**) [16], and carbazoles (**VII**) [19]. Minakata and co-workers proposed an innovative approach for transferring imine groups using iodane-containing (diarylmethylene)amino groups (**V**), which proved to be useful in the transfer of imine radicals [17]. Bolm et al. contributed also to this topic by introducing a sulfoximidoyl-containing benziodoxolone (**VI**) [18]. Recently, our group disclosed the first HIRs bearing a primary amine moiety, the benzylamine benziodoxolone reagent **VIII** (named BBX), and investigated its reactivity on the α-amination of indanone-based β-ketoesters (Scheme 1) [4]. Very recently, Minakata’s group also reported iodine(III) reagents with transferable amino groups, particularly a benziodoxolone bearing a transferable NH_2_ group (**IX**) [20].

HIRs have been a central theme of the work carried out by our research group in recent years, focusing on the formation of the S–N bond by applying the umpolung reactivity of HIRs, in particular in the preparation of sulfonamides and sulfonyl hydrazides [22–23]. In both approaches, a sulfonyl-containing benziodoxolone was generated in situ, via a reaction of chlorobenziodoxolone with sulfinate salts, followed by the addition of amines or hydrazines, respectively.

On the follow-up on our research, we envisaged to extend the diversity of BBX reagents as electrophilic amine reagents and investigated their reactivity with in situ-generated sulfenate anions, from β-sulfinyl esters, to achieve S–N bond formation. The importance of establishing this S–N bond results from the widespread presence of sulfonyl-containing bioactive compounds, such as the sulfonamide group which can be found in many pharmaceuticals, commonly referred to as sulfa drugs. These include top seller drugs, e.g., antimicrobials, anti-inflammatories, antihypertensives, and antitumor agents [24–26]. Particularly, the sulfonamide motif can act as a bioisostere of carboxylic acids, establishing a set of hydrogen bonds similar to those formed by carboxylic acids, enhancing its versatility and effectiveness in drug design [27].

Traditional sulfonamide preparation involves combining sulfonyl chlorides and amines [25,28–29]. Despite the efficiency of traditional methods, challenges still remain, e.g., use of harsh conditions, like oxidative chlorination with aqueous chlorine [30], or treatment with toxic sulfur dioxide. Thus, we envisaged to further investigate BBX reactivity to address the S–N-bond formation, as an alternative method towards sulfur-containing compounds [22].

## Results and Discussion

**Scheme 2:**
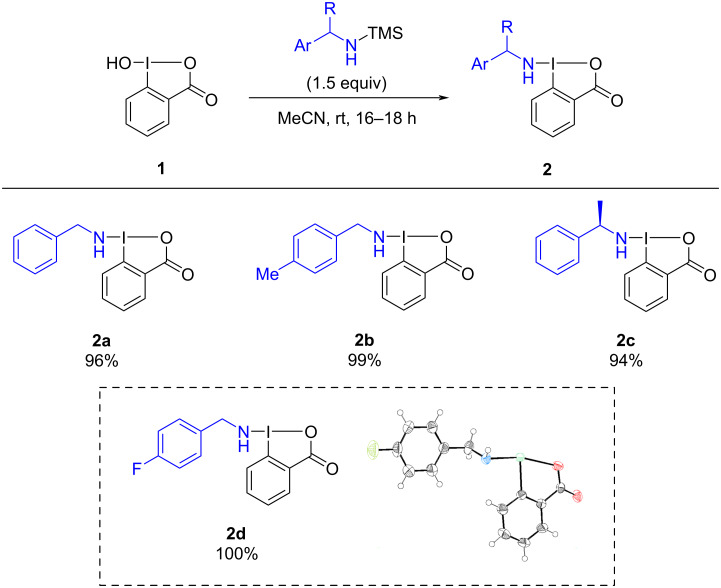
Scope of the different (benzylamino)benziodoxolones (BBXs) **2** with ORTEP-3 diagram of compound **2d**, using 50% probability level ellipsoids. One co-crystallized water molecule was omitted for clarity. CCDC 2368436 contains the supporting crystallographic data for this paper.

We initiated our study by extending the functionalization of BBX reagents. (Benzylamino)benziodoxolones, BBXs **2**, were prepared according to our reported procedure, via reaction with previously silylated benzylamines (Scheme 2) [4]. Using this methodology, a total of four benziodoxolones (including the new *p*-fluorobenzylamine benziodoxolone, **2d**) were obtained with quantitative yields, as white solids on the gram-scale, easy to manipulate and long-term stable below 0 °C.

Crystals of compound **2d** were successfully obtained and its molecular structure was confirmed through single-crystal X-ray diffraction. The X-ray analysis revealed a distorted T-shaped geometry, consistent with previously reported N–bound hypervalent iodine reagents. Additionally, the N–I bond distance is 2.0454(5) Å, which aligns with our previously reported values [4]. The two aromatic rings are nearly coplanar, exhibiting a dihedral angle of 0.7(3)°. All other bond lengths and angles fall within the expected range for similar compounds [31].

Later, the β-sulfinyl esters **4** were prepared by Michael addition reaction of thiols and α,β-unsaturated esters [32], followed by oxidation of the corresponding sulfides **3** using two different oxidizing agents (oxone and *m*-CPBA) [32–33]. To investigate the reactivity of the BBXs in this electrophilic amination reaction, the generated compound **4** was subjected to a retro-Michael addition to produce the sulfenate anion intermediate, followed by the addition of BBX **2**. Based on our experience with HIRs, the reaction of **2** with nucleophiles is more effective when a pre-formed nucleophile is used [4]. Thus, HIR **2** was added to the reaction mixture after the in situ formation of the sulfenate anion (by retro-Michael addition).

First experiments were carried out under the previously described conditions for BBX electrophilic amination reaction (Table 1, entry 1) [4]. In the presence of potassium carbonate, only starting material **4a** was detected. A stronger base to generate the nucleophilic intermediate was tested, and sulfonamide **5aa** was detected in trace amounts (Table 1, entry 2).

**Table 1:** Optimization of the electrophilic amination of *tert*-butyl 3-(*p*-tolylsulfinyl)propanoate (**4a**) with BBX **2a** (0.23 mmol of limitant in 2 mL of solvent).

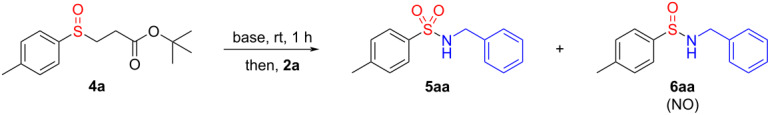							

Entry	**4a** (equiv)	**2a** (equiv)	Base (equiv)	Solvent	*T* (°C)	Time (h)	**5aa** (yield %)^a^

1	1	1.5	K_2_CO_3_ 1	DMF	50	3	NO
2	1	2	NaH 1	DMF	50	20	trace
3	1	2	NaH 1.2	PhMe	50	20	NO
4	1	2	NaH 1.2	DMF^b^	50	3	29
5	1	2	NaH 1.2	DMF^b^	rt	20	9
6	2	1	NaH 2.4	DMF^b^	50	6	38
**7**	**2**	**1**	**NaH** **2.4**	**DMF** ^b^	**50**	**20**	**52**
8	2	1	NaH 2.4	DMF^b^	50	72	48

^a^Isolated yields; ^b^degassed solvent; rt – room temperature; NO – not observed.

Considering the low solubility of the hypervalent reagent **2a** in most organic solvents, an alternative solvent was tested; nevertheless, BBX **2a** showed to be insoluble when using toluene (Table 1, entry 3).

To have further insights on the formation of sulfonamide **5aa**, an experiment was conducted under the same stoichiometric conditions that yielded product **5aa** (Table 1, entry 2) but with prior degassed solvent (DMF), to prevent potential oxidation of the sulfenate to sulfinate anions. Indeed, the oxidation of the unstable sulfenate intermediates has been previously reported by Waser when using EBX – an HIR applied in the transfer of alkynes to sulfenate salts [34]. Under these conditions, *N*-benzyl-4-methylbenzenesulfinamide (**6aa**) was not observed, and *N*-benzyl-*p*-toluenesulfonamide (**5aa**) was isolated in 29% yield (Table 1, entry 4).

To prevent the regeneration of *tert*-butyl 3-(*p*-tolylsulfinyl)propanoate (**4a**) and employ milder conditions, a study was conducted at room temperature for 20 hours, which resulted in a reduction of the yield for **5aa** to 9% (Table 1, entry 5). This result might be due to the reactivity of this hypervalent iodine reagent. Indeed, we have previously observed that the transfer of the benzylamine moiety to carbon-based nucleophiles is more favorable at higher temperatures [4].

Due to the reversibility of the retro-Michael addition, an experiment was carried out using an excess of *tert*-butyl 3-(*p*-tolylsulfinyl)propanoate (**4a**) leading to an increase in the yield of the corresponding sulfonamide **5aa** by 38% (Table 1, entry 6). A longer reaction time was tested, with the reaction running overnight, which led to an increase of 52% in the reaction yield (Table 1, entry 7), consisting of the best conditions achieved for this electrophilic amination. The reaction time was extended to 72 hours in an attempt to promote the transfer reaction; however, the corresponding sulfonamide **5aa** was obtained in 48% yield (Table 1, entry 8), thus proving that beyond 20 hours, the reaction does not develop any further.

The experiment carried out with degassed solvent (Table 1, entry 4) eliminated the hypothesis of sulfonamide **5aa** formation via oxidation of sulfinamide **6aa** by dissolved oxygen molecules in the media. Therefore, an additional experiment was conducted using sodium benzenesulfinate to simulate the potential in situ oxidation of the sulfenate anion before the addition of BBX **2a**, but only trace amounts of sulfonamide **5ea** were observed.

A deeper analysis of the composition of the crude mixture revealed the presence of sulfide **3a** and disulfide **7a**, which formation might probably result from an oxidative reaction involving species generated from 2 sulfenate molecules. A further experiment was carried out in the absence of light. Under these conditions, no amine-transfer products **5aa** (sulfonamide) or **6aa** (sulfinamide) were observed.

**Scheme 3:**
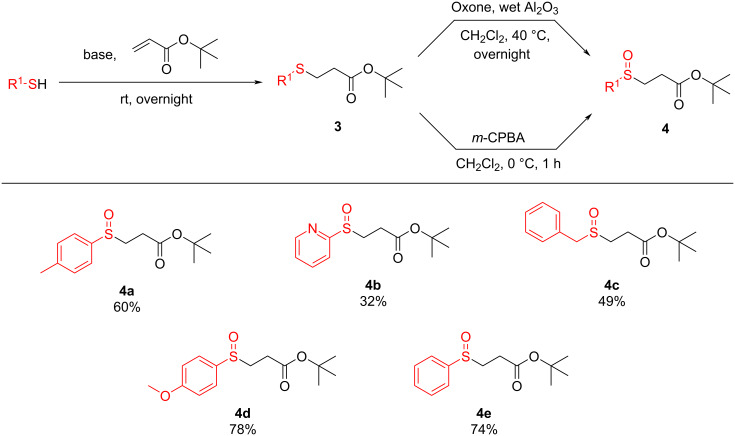
Scope of the different β-sulfinyl esters **4** [32–33]. Isolated yields. rt – room temperature.

Next, and with the optimized conditions in hand (Table 1, entry 7), we studied the scope of the reaction by varying both the β-sulfinyl esters **4** and the electrophilic amines **2**. Thus, a variety of functionalized thiols (aromatic, aliphatic, and heterocyclic) were chosen to produce the β-sulfinyl esters **4** (Scheme 3).

**Scheme 4:**
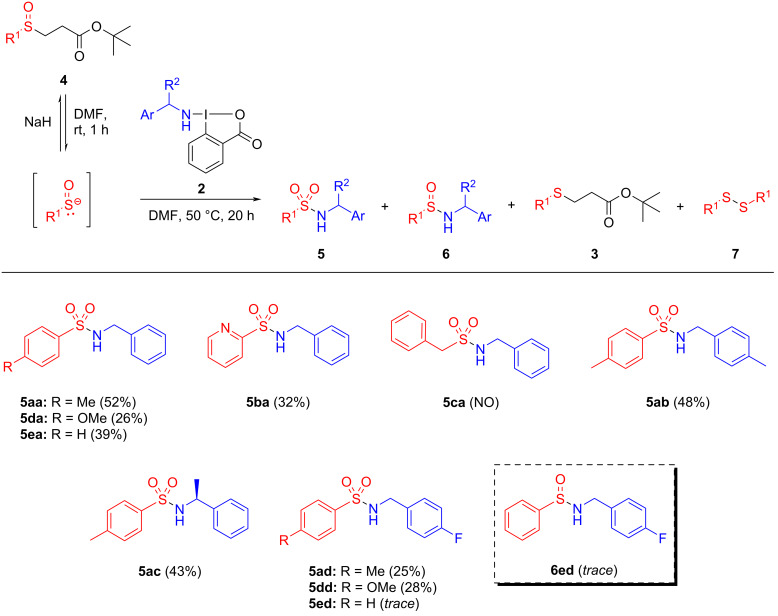
Scope of the primary amine electrophilic reaction of sulfenate salts. Reaction conditions: **4** (2 equiv), NaH (2.4 equiv), **2**, degassed DMF (0.055 M). Isolated yields. rt – room temperature; NO – not observed.

We next investigated the electrophilic amination reaction (Scheme 4). First, different functionalized β-sulfinyl esters were reacted with BBX **2a**. For the aromatic and pyridine moieties, the electrophilic amination reaction afforded sulfonamides **5aa**, **5ba**, **5da**, and **5ea** with moderate yields. However, no amination product was detected with *tert*-butyl 3-(benzylsulfinyl)propanoate (**4c**). This result suggests that aliphatic β-sulfinyl esters may not possess sufficient nucleophilicity to react with the primary amine-containing HIRs, or it might be due to the inability of the benzyl moiety to stabilize the sulfenate ion during the reaction.

For *tert*-butyl 3-(*p*-tolylsulfinyl)propanoate (**4a**), and similarly to the outcome obtained for BBX **2a**, sulfonamides **5ab** and **5ac** were obtained with moderate yields. The slight decrease observed for **5ac**, with chiral (*R*)-1-((1-phenylethyl)amino)-1,2-benziodoxol-3-(1*H*)-one (**2c**), can be attributed to potential steric hindrance induced by the methyl group attached to the benzylic carbon, which may hinder the nucleophile’s access to the electrophilic center of the HIR.

The new hypervalent reagent 1-(4-fluorobenzyl)amino-1,2-benziodoxol-3-(1*H*)-one (**2d**) was also tested in the amination reaction, leading to the formation of sulfonamides **5ad**, **5dd**, and **5ed** (25%, 28%, and trace amount, respectively). In the last example, the low amount of sulfonamide **5ed** obtained may result from the simultaneous formation of sulfinamide **6ea** also isolated in this reaction for the first time (both in trace amounts).

### Reaction mechanism

The inability to detect sulfinamide **6** and the isolation of sulfonamide **5**, along with other byproducts (**3**, **4**, and **7**), stimulated us to propose a plausible reaction mechanism that would support both the obtained yields and the formation of unexpected species.

**Scheme 5:**
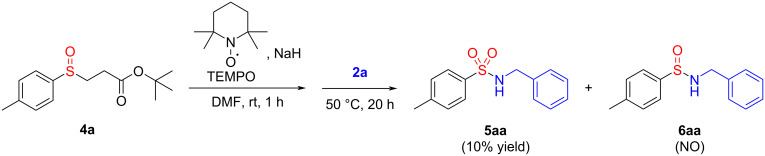
Electrophilic amination reaction in the presence of TEMPO. Reaction conditions: **4a** (2 equiv), NaH (2.4 equiv), **2a**, TEMPO (10 mol %), degassed DMF (0.055 M). NO – not observed.

As mentioned above, the presence of light influences the reaction outcome. When the reaction was carried out in the absence of light, only **4a**, **3a**, and **7a** were isolated (under these conditions, there was a high decrease in the isolated amount of **3a**, compared to the same experiment carried out in the presence of light, from 20% to 2% yield). Considering the potential occurrence of a radical pathway, additional experiments were conducted in the presence of galvinoxyl and TEMPO, powerful radical scavengers capable of abstracting the radical species that could emerge in the reaction media. The use of galvinoxyl proved to be insufficient to conclude since a control experiment showed that HIRs **2** decompose in the presence of galvinoxyl. When using TEMPO (Scheme 5), sulfinamide **6aa** was not detected, but a drastic decrease in the yield of sulfonamide **5aa** was observed, suggesting that the benzylamine-transfer reaction might occur via a radical mechanism. This finding supports the hypothesis that, beyond the ionic mechanism previously explored for our electrophilic primary amine transfer reagent [4], HIR **2a** might also be engaged in a radical mechanism contingent upon the medium or the species present.

**Scheme 6:**
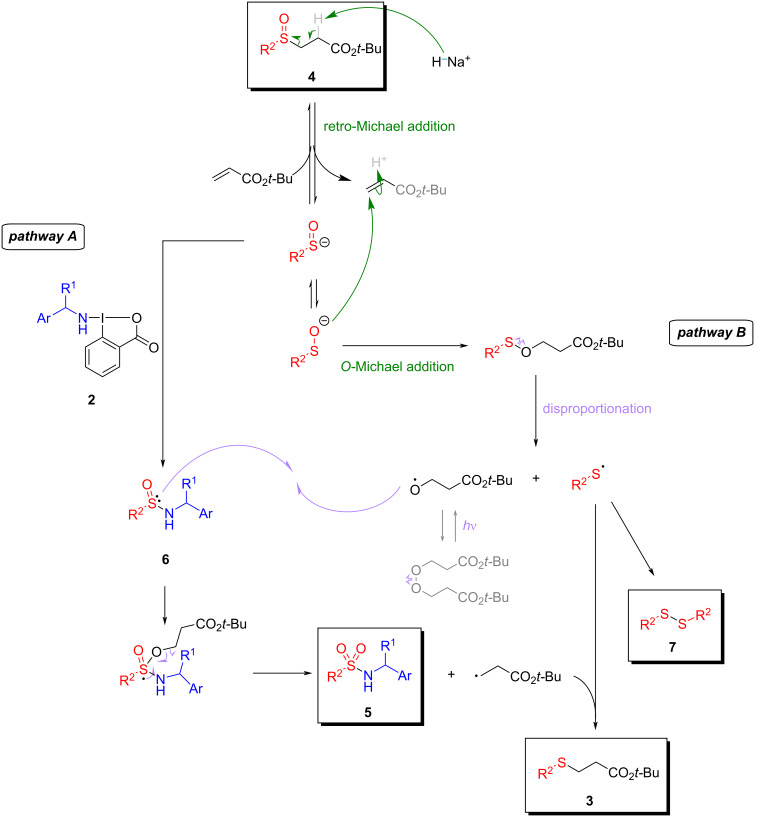
Mechanism proposed for sulfonamide **5**, β-sulfinyl ester **4**, disulfide **7**, and sulfide **3** formations. The ionic steps are illustrated in green, whereas the radical steps appear in purple [35].

Analysis of data from optimization and scope experiments provided valuable insights into the reaction, leading to a plausible mechanistic proposal (Scheme 6). This suggestion is based on the recognized ionic character of the sulfenate ion generated in the retro-Michael addition, on the results obtained with TEMPO (Scheme 5), and also on the results obtained when the reaction was carried out in the absence and presence of light, as well as the control experiments in the absence of BBX (see Supporting Information File 1).

We propose a mechanism pathway involving the retro-Michael addition of **4**, releasing acrylate and hydrogen (H_2_). The charge of the sulfenate anion may shift between sulfur and oxygen atoms, possibly leading to an *O*-Michael addition (pathway B) [35]. The intermediate of these reactions could undergo disproportionation, a radical process resulting in the homolytic cleavage of the S–O bond [35]. The formation of disulfide **7** isolated in the experiments can be explained by combining two radical sulfur species. Furthermore, the oxygen species generated in this radical reaction may dimerize to yield peroxide. However, the instability of peroxide favors the predominance of radical oxygen species, which can react with the sulfur atom from sulfinamide **6** previously formed in the reaction medium (pathway A). Following the establishment of the new S–O bond, a radical reaction akin to retro-Michael is expected, yielding sulfonamide **5** and a *C*-radical derivative from acrylate. This derivative may then combine with sulfur radical molecules to produce sulfide **3**, the final byproduct of this reaction.

## Conclusion

As mentioned above, HIRs have emerged as alternative reagents for conducting various transformations. The umpolung reactivity provided by these iodine reagents enables chemical transformations that would typically demand less environmentally friendly conditions. The investigations conducted in this work confirmed the ability of the novel hypervalent iodine(III) reagents **2** to transfer their amine moieties to various β-sulfinyl esters via an umpolung mechanism, generating the corresponding sulfonamides.

## Supporting Information

File 1 Experimental procedures, characterization data, NMR spectra, and X-ray diffraction data.

## Data Availability

All data that supports the findings of this study is available in the published article and/or the supporting information to this article.

## References

[1] Kiyokawa K, Minakata S (2020). Synlett.

[2] Yoshimura A, Zhdankin V V (2016). Chem Rev.

[3] Li Y, Hari D P, Vita M V, Waser J (2016). Angew Chem, Int Ed.

[4] Poeira D L, Negrão A C R, Faustino H, Coelho J A S, Gomes C S B, Gois P M P, Marques M M B (2022). Org Lett.

[5] Makitalo C L, Yoshimura A, Rohde G T, Mironova I A, Yusubova R Y, Yusubov M S, Zhdankin V V, Saito A (2020). Eur J Org Chem.

[6] Ishihara K, Muñiz K (2022). Iodine Catalysis in Organic Synthesis.

[7] Olofsson B, Marek I, Rappoport Z (2019). Patai’s Chemistry of Functional Groups: The Chemistry of Hypervalent Halogen Compounds.

[8] Wirth T (2016). Hypervalent Iodine Chemistry: Modern Developments in Organic Synthesis.

[9] Sajith P K, Suresh C H (2013). Inorg Chem.

[10] Hari D P, Caramenti P, Waser J (2018). Acc Chem Res.

[11] Kiprof P (2005). ARKIVOC.

[12] Macara J, Caldeira C, Poeira D L, Marques M M B (2023). Eur J Org Chem.

[13] Zhdankin V V, Kuehl C J, Bolz J T, Formaneck M S, Simonsen A J (1994). Tetrahedron Lett.

[14] Zhdankin V V, McSherry M, Mismash B, Bolz J T, Woodward J K, Arbit R M, Erickson S (1997). Tetrahedron Lett.

[15] Zhang Y, Lu J, Lan T, Cheng S, Liu W, Chen C (2021). Eur J Org Chem.

[16] Kiyokawa K, Kosaka T, Kojima T, Minakata S (2015). Angew Chem, Int Ed.

[17] Okumatsu D, Kawanaka K, Kainuma S, Kiyokawa K, Minakata S (2023). Chem – Eur J.

[18] Wang H, Zhang D, Sheng H, Bolm C (2017). J Org Chem.

[19] Lan T, Qin H, Chen W, Liu W, Chen C (2020). Chin Chem Lett.

[20] Kiyokawa K, Kawanaka K, Minakata S (2024). Angew Chem, Int Ed.

[21] Zhdankin V V, Kuehl C J, Krasutsky A P, Formaneck M S, Bolz J T (1994). Tetrahedron Lett.

[22] Poeira D L, Macara J, Faustino H, Coelho J A S, Gois P M P, Marques M M B (2019). Eur J Org Chem.

[23] Macara J, Caldeira C, Cunha J, Coelho J A S, Silva M J S A, Krämer K, Grathwol C W, Bräse S, Marques M M B (2023). Org Biomol Chem.

[24] Drews J (2000). Science.

[25] Mondal S, Malakar S (2020). Tetrahedron.

[26] Ovung A, Bhattacharyya J (2021). Biophys Rev.

[27] Wan Y, Fang G, Chen H, Deng X, Tang Z (2021). Eur J Med Chem.

[28] De Luca L, Giacomelli G (2008). J Org Chem.

[29] Wright S W, Hallstrom K N (2006). J Org Chem.

[30] Bahrami K, Khodaei M M, Soheilizad M (2009). J Org Chem.

[31] Groom C R, Bruno I J, Lightfoot M P, Ward S C (2016). Acta Crystallogr, Sect B: Struct Sci, Cryst Eng Mater.

[32] Maitro G, Prestat G, Madec D, Poli G (2006). J Org Chem.

[33] Wang L, Chen M, Zhang P, Li W, Zhang J (2018). J Am Chem Soc.

[34] Amos S G E, Nicolai S, Gagnebin A, Le Vaillant F, Waser J (2019). J Org Chem.

[35] Zhang Q, Feng H, Chen F, He Z, Zeng Q (2021). J Org Chem.

