# Supplementary material for: Reactivity of hypervalent iodine(III) reagents bearing a benzylamine with sulfenate salts
Source: Beilstein J Org Chem. 2024 Dec 19;20:3281–9. doi: 10.3762/bjoc.20.272 (PMC11665445; doi:10.3762/bjoc.20.272)
Supplement: File 1 — Experimental procedures, characterization data, NMR spectra, and X-ray diffraction data. [file Beilstein_J_Org_Chem-20-3281-s001.pdf]

BEILSTEIN JOURNAL OF ORGANIC CHEMISTRY

Supporting Information

for

Reactivity of hypervalent iodine(III) reagents bearing a benzylamine with sulfenate salts

Beatriz Dedeiras, Catarina S. Caldeira, José C. Cunha, Clara S. B. Gomes and M. Manuel B. Marques

Beilstein J. Org. Chem. **2024,** *20,* 3281–3289. doi:10.3762/bjoc.20.272

Experimental procedures, characterization data, NMR spectra, and X-ray diffraction data

License and Terms: This is a supporting information file under the terms of the Creative Commons Attribution License (https://creativecommons.org/licenses/by/4.0). Please note that the reuse, redistribution and reproduction in particular requires that the author(s) and source are credited and that individual graphics may be subject to special legal provisions.
The license is subject to the *Beilstein Journal of Organic Chemistry* terms and conditions: (https://www.beilstein-journals.org/bjoc/terms)

Table of contents

1. General information S2

2. Synthesis of primary amine-containing hypervalent iodine reagents...................S3

3. Preparation of starting materials S7

4. General procedure of electrophilic amination of sulfenate salts S17

5. NMR Studies to Investigate the formation of **3** and **7**..S23

6. Single crystal X-ray crystallography study S30

7. References S32

8. NMR spectra S34

1. General information

Various commercially available purchased reagents were employed and used without further purification, except when indicated. Solvents, when referenced, were pre-dried using conventional methods. 3 Å molecular sieves were activated by heating at 300 °C in a muffle furnace for 3 h.

Analytical TLC was performed on Macherey-NaGel-0.20 mm silica gel 60 with fluorescent indicator UV_{254} supported on aluminium. Preparative TLC was conducted on Merck silica gel 60 with fluorescent indicator UV_{254} supported on a 0.5 mm glass surface, using the described eluent system for each case. Normal phase silica gel flash chromatography was carried out using Carlo Erba silica gel 60 Å and the described eluent system for each case.

IR spectra were acquired using a Perkin-Elmer Spectrum Two FT-IR spectrometer equipped with a UATR module. Transmittance of the samples were acquired between 4000 and 400 cm^{-1}, with resulting IR bands reported in cm^{-1} and categorized as weak (w), medium (m) or strong (s).

NMR spectra were measured with a Bruker ARX 400 spectrometer. ^{1}H NMR, ^{13}C NMR and ^{19}F NMR were acquired at 400, 101 and 376 MHz, respectively. Samples were prepared in 0.5 mm NMR tubes using either $CDCl_3$ or DMSO-d_6 as solvents. NMR data were recorded in terms of chemical shift in ppm (with the corresponding trace $CHCl_3$ or DMSO-d_6 used as reference signals), signal multiplicity, coupling constants (J) in Hz, and integration. Signal multiplicity was denoted as singlet (s), broad singlet (br. s), doublet (d), dd (doublet of doublets), dt (doublet of triplets), ddd (doublet of doublets of doublets), t (triplet), td (triplet of doublets), q (quartet), p (quintuple) and multiple (m).

High-resolution mass spectrometry (HRMS) analyses were conducted by liquid chromatography–mass spectrometry (LC–MS) runs using a Dionex Ultimate 3000 UHPLC+ system equipped with a Multiple-Wavelength detector, an imChem Surf C18 TriF 100A 3 µm 100 × 2, 1 mm column connected to Thermo Scientific Q Exactive hybrid quadrupole-Orbitrap mass spectrometer (Thermo Scientific™ Q Exactive™ Plus).

2. Synthesis of primary amine-containing hypervalent iodine reagents

2.1. Synthesis of 1-hydroxy-1,2-benziodoxol-3-(1*H*)-one (1) [1]

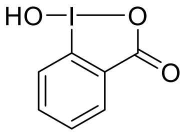

A round-bottom flask was charged with 2-iodobenzoic acid (4.00 g, 16.12 mmol) and sodium periodate (3.62 g, 16.94 mmol, 1.05 equiv). Subsequently, a solution of 30% acetic acid in water (24 mL) was added, and the resulting mixture was refluxed at 120 °C for 4 hours. Afterward, 90 mL of ice-cold water was poured in, followed by filtration of the suspension and successive washes with water (3 × 10 mL) and acetone (3 × 10 mL). The obtained solid was then subjected to vacuum desiccation to remove excess solvents, affording 1-hydroxy-1,2-benziodoxol-3-(1*H*)-one (**1**) as a crystalline white solid in 97% yield.

The product is already reported and spectral data are in accordance with literature values[1].

m.p.: 223 ºC.

IR(ATR): 3083 (w), 3060 (w), 2868 (b), 1601 (m), 1584 (m), 1558 (m), 1439 (m), 579 (m).

^{1}H NMR (400 MHz, DMSO-*d₆*): δ_H 8.01 (d, *J* = 7.5 Hz, 1H), 7.99 – 7.92 (m, 1H), 7.84 (d, *J* = 8.1 Hz, 1H), 7.70 (t, *J* = 7.3 Hz, 1H).

^{13}C NMR (101 MHz, DMSO-d_6): δ_C 167.7, 134.5, 131.5, 131.1, 130.4, 126.3, 120.4.

2.2. Preparation of primary amine-containing hypervalent iodine reagents 2 [2]

1-Benzylamino-1,2-benziodoxol-3-(1*H*)-one (2a)

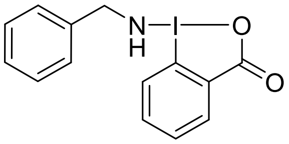

A round-bottom flask was charged with hydroxybenziodoxolone **1** and dry acetonitrile (0.45 M), followed by the addition of the solution of 1,1,1-trimethyl-*N*-benzylsilanamine (**S1**, 1.50 equiv). The mixture was allowed to stir overnight in darkness. The next day, additional 10 mL of acetonitrile were added to the solution and the flask's content was transferred into Falcon tubes, which were subjected to centrifugation (10 min, RCF = 283.5 (G-force)). The

supernatant was discharged, and the resulting pellets underwent 3 additional washes with acetonitrile. Finally, the combined pellets were dried thoroughly under vacuum, to yield the desired product **2a** as a white solid in 96% yield.

The product is already reported and spectral data are in accordance with literature values[2].

m.p.: 115-118 ºC.

IR(ATR): 3194 (m), 3057 (w), 2930 (w), 1595 (m), 1496 (w), 1466 (w), 1455 (m), 1435 (m), 1191 (w), 520 (m).

^{1}H NMR (400 MHz, DMSO-*d*$_6$): δ_H 8.09 – 8.02 (m, 1H), 8.00 (dd, *J* = 7.5, 1.6 Hz, 1H), 7.83 (ddd, *J* = 8.4, 7.2, 1.6 Hz, 1H), 7.63 (td, *J* = 7.3, 1.0 Hz, 1H), 7.45 – 7.26 (m, 6H), 5.86 (t, *J* = 6.2 Hz, 1H), 4.48 (d, *J* = 5.4 Hz, 2H).

^{13}C NMR (101 MHz, DMSO-*d*$_6$): δ_C 166.8, 140.7, 133.4, 133.2, 131.2, 130.0, 128.5, 128.4, 127.5, 125.7, 116.9, 54.5.

1-((4-Methylbenzyl)amino)-1,2-benziodoxol-3-(1*H*)-one (2b)

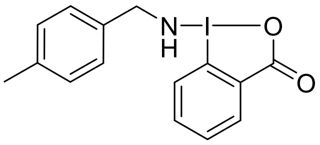

A round-bottom flask was charged with hydroxybenziodoxolone **1** (0.931 g, 3.53 mmol) and 7.7 mL dry acetonitrile, followed by the addition of the solution of 1,1,1-trimethyl-*N*-(4-methylbenzyl)silanamine (**S1**, 5.25 mmol, 1.5 equiv). The mixture was allowed to stir overnight in darkness. The next day, additional 10 mL of acetonitrile were added to the solution and the flask's content was transferred into Falcon tubes, which were subjected to centrifugation (10 min, RCF = 283.5 (G-force)). The supernatant was discharged, and the resulting pellets underwent 3 additional washes with acetonitrile. Finally, the combined pellets were dried thoroughly under vacuum, to yield the desired product **2b** as a white solid (1.27 g, 3.47 mmol, 99% yield).

The product is already reported and spectral data are in accordance with literature values[2].

m.p.: 115-116 ºC.

IR(ATR): 3210 (m), 3080 (w), 3059 (w), 2916 (w), 1593 (m), 1512 (m), 1461 (m), 1437 (m), 1147 (w), 592 (m).

^{1}H NMR (400 MHz, DMSO-*d*$_6$): δ$_H$ 8.05 (dd, *J* = 8.2, 1.0 Hz, 1H), 8.00 (dd, *J* = 7.5, 1.6 Hz, 1H), 7.83 (ddd, *J* = 8.4, 7.1, 1.6 Hz, 1H), 7.63 (td, *J* = 7.3, 1.0 Hz, 1H), 7.30 (d, *J* = 8.0 Hz, 2H), 7.15 (d, *J* = 7.8 Hz, 2H), 5.82 (t, *J* = 6.1 Hz, 1H), 4.42 (d, *J* = 5.7 Hz, 2H), 2.26 (s, 3H).

^{13}C NMR (101 MHz, DMSO-*d*$_6$): δ$_C$ 166.8, 137.7, 136.7, 133.4, 133.2, 131.2, 130.0, 129.0, 128.4, 125.7, 116.8, 54.2, 20.8.

(*R*)-1-((1-Phenylethyl)amino)-1,2-benziodoxol-3-(1*H*)-one (2c)

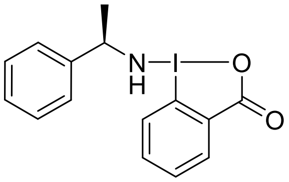

A round-bottom flask was charged with hydroxybenziodoxolone **1** (561.44 mg, 2.13 mmol) and 4.7 mL dry acetonitrile, followed by the addition of the solution of (*R*)-1,1,1-trimethyl-*N*-(1-phenylethyl)silanamine (**S3**, 3.19 mmol, 1.5 equiv). The mixture was allowed to stir overnight in darkness. The next day, additional 10 mL of acetonitrile were added to the solution and the flask's content was transferred into Falcon tubes, which were subjected to centrifugation (10 min, RCF = 283.5 (G-force)). The supernatant was discharged, and the resulting pellets underwent 3 additional washes with acetonitrile. Finally, the combined pellets were dried thoroughly under vacuum, to yield the desired product **2c** as a white solid (732.55 mg, 2.00 mmol, 94% yield).

The product is already reported and spectral data are in accordance with literature values [2].

m.p.: 114-115 ºC.

IR(ATR): 3209 (m), 3073 (w), 3053 (w), 3969 (w), 2922 (w), 1598 (m), 1581 (m), 1558 (m), 1434 (m), 1341 (m), 750 (s), 570 (m).

^{1}H NMR (400 MHz, DMSO-*d*$_6$): δ$_H$ 8.13 (dd, *J* = 8.2, 1.0 Hz, 1H), 7.98 (dd, *J* = 7.5, 1.6 Hz, 1H), 7.84 (ddd, *J* = 8.4, 7.1, 1.6 Hz, 1H), 7.62 (td, *J* = 7.3, 1.0 Hz, 1H), 7.46 – 7.39 (m, 2H),

7.36 – 7.27 (m, 2H), 7.29 – 7.20 (m, 1H), 5.97 (d, *J* = 3.8 Hz, 1H), 4.56 (qd, *J* = 6.5, 3.6 Hz, 1H), 1.46 (d, *J* = 6.6 Hz, 3H).

^{13}C NMR (101 MHz, DMSO-*d₆*): δ_C 166.8, 144.5, 133.4, 133.1, 131.12, 130.0, 128.5, 127.7, 126.8, 125.8, 117.0, 58.5, 24.3.

1-((4-Fluorobenzyl)amino)-1,2-benziodoxol-3-(1*H*)-one (2d)

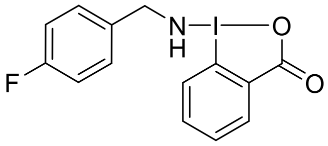

A round-bottom flask was charged with hydroxybenziodoxolone **1** (2.18 g, 8.26 mmol) and 18 mL dry acetonitrile, followed by the addition of the solution of 1,1,1-trimethyl-*N*-(4-fluorobenzyl)silanamine (**S4**, 12.21 mmol, 1.5 equiv). The mixture was allowed to stir overnight in darkness. The next day, additional 10 mL of acetonitrile were added to the solution and the flask's content was transferred into Falcon tubes, which were subjected to centrifugation (10 min, RCF = 283.5 (G-force)). The supernatant was discharged, and the resulting pellets underwent 3 additional washes with acetonitrile. Finally, the combined pellets were dried thoroughly under vacuum, to yield the desired product **2d** as a white solid (3.06 g, 8.24 mmol, 100% yield).

m.p.: 128-130 °C.

IR(ATR): 3193 (m), 3069 (w), 3056 (w), 2927 (w), 1594 (m), 1562 (m), 1507 (m), 1464 (m), 1436 (m), 1221 (m), 1160 (m), 582 (m).

^{1}H NMR (400 MHz, DMSO-*d₆*): δ_H 8.03 (dd, *J* = 8.2, 1.0 Hz, 1H), 8.00 (dd, *J* = 7.5, 1.5 Hz, 1H), 7.83 (ddd, *J* = 8.2, 7.1, 1.6 Hz, 1H), 7.63 (td, *J* = 7.3, 1.0 Hz, 1H), 7.46 (dd, *J* = 8.5, 5.8 Hz, 2H), 7.16 (t, *J* = 8.9 Hz, 2H), 5.84 (t, *J* = 6.1 Hz, 1H), 4.46 (d, *J* = 6.1 Hz, 2H).

^{13}C NMR (101 MHz, DMSO-*d₆*): δ_C 166.9, 161.3 (d, *J* = 243.3 Hz), 137.0 (d, *J* = 3.2 Hz), 133.4, 133.2, 131.2, 130.50 (d, *J* = 8.1 Hz), 130.1, 125.7, 116.9, 115.2 (d, *J* = 21.3 Hz), 53.6.

^{19}F NMR (376 MHz, DMSO-*d₆*): δ_F -110.19.

HRMS (ESI$^+$) Calculated for $C_{14}H_{11}FINNaO_2$ $[M+H]^+$: 393.9711, found 393.9694.

3. Preparation of starting materials

3.1. Synthesis of 1,1,1-trimethyl-*N*-benzylsilanamine (S1)

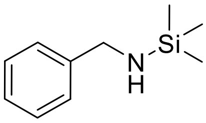

Adapted from the literature [3–5], a flame-dried Schlenk tube was charged with molecular sieves (3 Å), benzylamine (1.50 mL, 13.73 mmol) and 15 mL dry dichloromethane. The solution was placed in an ice bath, and dry triethylamine (1.90 mL, 13.65 mmol, 1 equiv) was added dropwise. The solution was stirred for 30 minutes at 0 °C. After that time, *N*,*O*-bis(trimethylsilyl)acetamide (BSA, 3.36 mL, 13.73 mmol, 1 equiv) was added dropwise, and the mixture was allowed to warm to room temperature. After 2 hours, the resulting suspension was diluted with hexane (5 mL) and diethyl ether (5 mL), filtrated under a N_2 atmosphere, and evaporated under reduced pressure. 4.63 g of a 1.19:1 mixture of acetamide (resulting from BSA) and 1,1,1-trimethyl-*N*-benzylsilanamine (**S1**) was obtained as a light-yellow liquid. The desired compound was obtained in 86% yield (calculated by NMR). The mixture was used in the next reaction without additional purification.

The product is already reported and spectral data are in accordance with literature values[2].

^{1}H NMR (400 MHz, $CDCl_3$): δ_H 7.36 – 7.28 (m, 4H), 7.25 – 7.19 (m, 1H), 4.98 (br. s, 1H), 3.86 (s, 2H), 0.23 (s, 8H); signal corresponding to acetamide[6]: 2.00 (s, 4H).

3.2. Synthesis of 1,1,1-trimethyl-*N*-(4-methylbenzyl)silanamine (S2)

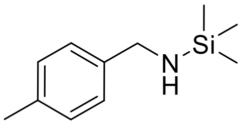

Adapted from the literature [3–5], a flame-dried Schlenk tube was charged with molecular sieves (3 Å), 4-methylbenzylamine (2.00 mL, 15.53 mmol) and 17 mL dry dichloromethane. The solution was placed in an ice bath, and dry triethylamine (2.20 mL, 15.81 mmol, 1 equiv) was added dropwise. The solution was stirred for 30 minutes at 0 °C. After that time, *N*,*O*-bis(trimethylsilyl)acetamide (BSA, 3.80 mL, 15.54 mmol, 1 equiv) was added dropwise, and

the mixture was allowed to warm to room temperature. After 2 hours, the resulting suspension was diluted with hexane (5 mL) and diethyl ether (5 mL), filtrated under a N_2 atmosphere, and evaporated under reduced pressure. 4.80 g of a 1.47:0.53:1 mixture of acetamine (resulting from BSA) [6], 4-methylbenzylamine and 1,1,1-trimethyl-*N*-(4-methylbenzyl)silanamine (**S2**) was obtained as a light-yellow liquid. The desired compound was obtained in 53% yield (calculated by NMR). The mixture was used in the next reaction without additional purification.

The product is already reported and spectral data are in accordance with literature values.[2]

^{1}H NMR (400 MHz, $CDCl_3$): δ_H 7.23 – 7.07 (m, 7H), 4.96 (br. s, 1H), 3.82 (s, 2H), 2.33 (s, 3H), 0.23 (s, 9H); signal corresponding to acetamide: 2.01 (s, 4H); signals corresponding to 4-methylbenzylamine: 7.23 – 7.07 (m, 7H), 3.91 – 3.84 (m, 1H), 2.32 (s, 2H), 1.36 (br. s, 1H).

3.3. Synthesis of (*R*)-1,1,1-trimethyl-*N*-(1-phenylethyl)silanamine (S3)

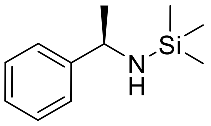

Adapted from the literature [3–5], a flame-dried Schlenk tube was charged with molecular sieves (3 Å), (*R*)-(+)-α-methylbenzylamine (1.20 mL, 9.33 mmol) and 10 mL dry dichloromethane. The solution was placed in an ice bath, and dry triethylamine (1.30 mL, 9.33 mmol, 1 equiv) was added dropwise. The solution was stirred for 30 minutes at 0 °C. After that time, *N*,*O*-bis(trimethylsilyl)acetamide (BSA, 2.30 mL, 9.41 mmol, 1 equiv) was added dropwise, and the mixture was allowed to warm to room temperature. After 2 hours, the resulting suspension was diluted with hexane (5 mL) and diethyl ether (5 mL), filtrated under a N_2 atmosphere, and evaporated under reduced pressure. 2.90 g of a 1.18:1 mixture of acetamide (resulted from BSA) [6] and (*R*)-1,1,1-trimethyl-*N*-(1-phenylethyl)silanamine (**S3**) was obtained as a light-yellow liquid. The desired compound was obtained in 74% yield (calculated by NMR). The mixture was used in the next reaction without additional purification.

The product is already reported and spectral data are in accordance with literature values [2].

^{1}H NMR (400 MHz, CDCl$_3$): δ$_H$ 7.35 – 7.28 (m, 5H), 4.95 (br. s, 1H), 4.11 (q, *J* = 6.6 Hz, 1H), 1.39 (d, *J* = 6.6 Hz, 3H), 0.23 (s, 9H); signal corresponding to acetamide: 2.01 (s, 4H).

3.4. Synthesis of 1,1,1-trimethyl-*N*-(4-fluorobenzyl)silanamine (S4)

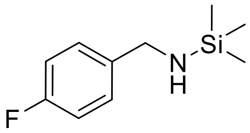

Adapted from the literature [3–5], a flame-dried Schlenk tube was charged with molecular sieves (3 Å), 4-fluorobenzylamine (2.00 mL, 17.32 mmol) and 19 mL dry dichloromethane. The solution was placed in an ice bath, and dry triethylamine (2.41 mL, 17.32 mmol, 1 equiv) was added dropwise. The solution was stirred for 30 minutes at 0 °C. After that time, *N*,*O*-bis(trimethylsilyl)acetamide (BSA, 4.23 mL, 17.32 mmol, 1 equiv) was added dropwise, and the mixture was allowed to warm to room temperature. After 2 hours, the resulting suspension was diluted with hexane (5 mL) and diethyl ether (5 mL), filtrated under a N_2 atmosphere, and evaporated under reduced pressure. 8.02 g of a 2.33:1 mixture of *N*-trimethylsilylacetamide (resulted from BSA) [6] and 1,1,1-trimethyl-*N*-(4-fluorobenzyl)silanamine (**S4**) was obtained as a yellow liquid. The desired compound was obtained in 70% yield (calculated by NMR). The mixture was used in the next reaction without additional purification.

The product is already reported and spectral data are in accordance with literature values[7].

^{1}H NMR (400 MHz, CDCl$_3$): δ$_H$ 7.29 – 7.26 (m, 2H), 7.01 (t, *J* = 8.6 Hz, 2H), 4.95 (br. s, 1H), 3.84 (s, 2H), 0.15 (s, 9H); signals corresponding to *N*-trimethylsilylacetamide[6]: 2.01 (s, 7H), 0.23 (s, 13H).

3.5. Synthesis of *tert*-butyl 3-(*p*-tolylthio)propanoate (3a) [8]

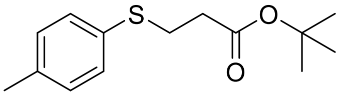

A round-bottom flask was charged with 4-methylthiophenol (0.50 mL, 4.00 mmol), potassium carbonate (27.64 mg, 0.2 mmol, 5 mol %) and 5 mL of dry dichloromethane. Subsequently, *tert*-butyl acrylate (0.64 mL, 4.40 mmol, 1.1 equiv) was rapidly added to the mixture, which was then stirred for 18 hours at room temperature. Afterward, the mixture was washed with water and the recovered organic phase was dried over anhydrous Na_2SO_4 and evaporated under reduced pressure. 765.30 mg of a 1:1 mixture of 4-methylthiophenol and *tert*-butyl 3-(*p*-tolylthio)propanoate (**3a**) was obtained as a yellow oil. The desired compound was obtained in 38% yield (calculated by NMR). The mixture was used in the next reaction without additional purification.

The product is already reported and spectral data are in accordance with literature values[8].

^{1}H NMR (400 MHz, CDCl$_3$): δ_H 7.31 – 7.27 (m, 2H), 7.21 – 7.16 (m, 2H), 3.38 (s, 1H), 2.51 (t, *J* = 7.5 Hz, 2H), 2.33 (s, 3H), 1.45 (s, 9H); signals corresponding to 4-methylthiophenol: 7.11 (d, *J* = 7.8 Hz, 2H), 7.05 (d, *J* = 7.9 Hz, 2H), 3.08 (t, *J* = 7.5 Hz, 2H), 2.30 (s, 3H).

3.6. Synthesis of *tert*-butyl 3-(pyridine-2-ylthio)propanoate (3b) [8]

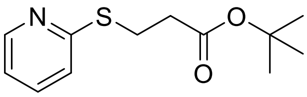

A round-bottom flask was charged with 2-mercaptopyridine (447.98 mg, 4.03 mmol), potassium carbonate (27.88 mg, 0.20 mmol, 5 mol %) and 5 mL of dry dichloromethane. Subsequently, *tert*-butyl acrylate (0.70 mL, 4.78 mmol, 1.2 equiv) was rapidly added to the mixture, which was then stirred for 16 hours at room temperature. Afterward, the mixture was washed with water and the recovered organic phase was dried over anhydrous Na_2SO_4 and evaporated under reduced pressure. Purification by flash chromatography (100% pentane to pentane/ethyl acetate 7:3) gave *tert*-butyl 3-(pyridine-2-ylthio)propanoate (**3b**) as a yellow oil in 52% yield.

The product is already reported and spectral data are in accordance with literature values[9].

IR(ATR): 2980 (w), 1727 (m), 1579 (m), 1368 (m), 1317 (m), 1153 (m), 1112 (m).

^{1}H NMR (400 MHz, CDCl$_3$): δ_H 8.41 (ddd, *J* = 5.1, 1.9, 1.0 Hz, 1H), 7.46 (ddd, *J* = 8.1, 7.3, 1.9 Hz, 1H), 7.15 (dt, *J* = 8.2, 1.0 Hz, 1H), 6.96 (ddd, *J* = 7.3, 4.9, 1.1 Hz, 1H), 3.38 (t, *J* = 7.1 Hz, 2H), 2.68 (t, *J* = 7.1 Hz, 2H), 1.45 (s, 9H).

^{13}C NMR (101 MHz, CDCl$_3$): δ_C 171.5, 158.7, 149.6, 136.0, 122.5, 119.5, 80.9, 53.6, 36.0, 28.3, 25.3.

3.7. *tert*-Butyl 3-(benzylthio)propanoate (3c) [8]

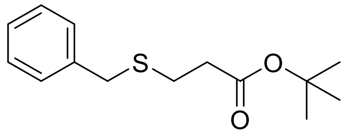

A round-bottom flask was charged with benzylmercaptan (1.24 mL, 10.00 mmol), potassium carbonate (69.10 mg, 0.5 mmol, 5 mol %) and 10 mL of dry dichloromethane. Subsquently, *tert*-butyl acrylate (1.60 mL, 11.00 mmol, 1.1 equiv) was rapidly added to the mixture, which was then stirred for 18 hours at room temperature. Afterward, the mixture was washed with water and the recovered organic phase was dried over anhydrous Na_2SO_4 and evaporated under reduced pressure. 1.58 g of a 1:1 mixture of benzylmercaptan and *tert*-butyl 3-(benzylthio)propanoate (**3c**) was obtained as a yellow oil. The desired compound was obtained in 31% yield (calculated by NMR). The mixture was used in the next reaction without additional purification.

The product is already reported and spectral data are in accordance with literature values[8].

^{1}H NMR (400 MHz, CDCl$_3$): δ_H 7.32 (t, *J* = 4.1 Hz, 10H), 3.60 (s, 2H), 2.65 (t, *J* = 7.4 Hz, 1H), 2.47 (t, *J* = 7.4 Hz, 1H), 1.76 (t, *J* = 7.6 Hz, 1H), 1.54 (s, 9H); signals corresponding to benzyl mercaptan: 7.32 (t, *J* = 4.1 Hz, 10H), 3.75 (d, *J* = 7.6 Hz, 2H), 3.73 (s, 1H).

***tert*-Butyl 3-(4-methoxyphenylthio)propanoate (3d)** [8]

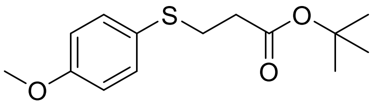

A round-bottom flask was charged with 4-methoxythiophenol (0.50 mL, 4.07 mmol), potassium carbonate (28.09 mg, 0.20 mmol, 5 mol %) and 5 mL of dry dichloromethane. Subsequently, *tert*-butyl acrylate (0.44 mL, 4.84 mmol, 1.2 equiv) was rapidly added to the mixture, which was then stirred for 24 hours at room temperature. Afterward, the mixture was washed with water and the recovered organic phase was dried over anhydrous Na_2SO_4 and evaporated under reduced pressure. 931.70 mg of a 0.79:1 mixture of 4-methoxythiophenol and *tert*-butyl 3-(4-methoxyphenylthio)propanoate (**3d**) was obtained as a yellow oil. The desired compound was obtained in 50% yield (calculated by NMR). The mixture was used in the next reaction without additional purification.

The product is already reported and spectral data are in accordance with literature values[8].

^{1}H NMR (400 MHz, $CDCl_3$): δ_H 7.82 (dd, *J* = 8.8, 1.9 Hz, 2H), 7.32 – 7.28 (m, 2H), 4.25 (d, *J* = 1.8 Hz, 3H), 3.46 (td, *J* = 7.5, 1.8 Hz, 2H), 2.92 (td, *J* = 7.5, 1.9 Hz, 2H), 1.90 (s, 9H); signals corresponding to 4-methoxythiophenol: 7.71 (d, *J* = 6.9 Hz, 2H), 7.27 – 7.22 (m, 2H), 4.23 (d, *J* = 1.8 Hz, 2H), 3.81 (s, 1H).

3.8. *tert*-Butyl 3-(phenylsulfanyl)propanoate (3e) [8]

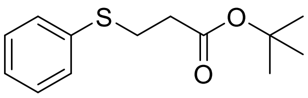

A round-bottom flask was charged with thiophenol (0.30 mL, 3.0 mmol, 1.2 equiv), sodium hydride (71.32 mg, 3.0 mmol, 1.2 equiv) and 5 mL of dry DMF. After stirring for 30 minutes at 0 °C, *tert*-butyl acrylate (0.37 mL, 2.48 mmol) was added to the mixture dropwise, and the mixture was then stirred for additional 66 hours at room temperature. Afterward, the obtained solution was washed with water and the recovered organic phase was dried over anhydrous Na_2SO_4 and evaporated under reduced pressure. 558.20 mg of a 0.35:1 mixture of thiophenol and *tert*-butyl 3-(phenylsulfanyl)propanoate (**3e**) was obtained as a yellow oil. The

desired compound was obtained in 70% yield (calculated by NMR). The mixture was used in the next reaction without additional purification.

The product is already reported and spectral data are in accordance with literature values[8].

^{1}H NMR (500 MHz, CDCl$_3$): δ$_H$ 7.52 – 7.47 (m, 1H), 7.40 – 7.34 (m, 2H), 7.32 – 7.27 (m, 3H), 7.21 (d, *J* = 7.3 Hz, 1H), 3.13 (t, *J* = 7.5 Hz, 2H), 2.54 (t, *J* = 7.5 Hz, 2H), 1.45 (s, 9H).

3.9. Synthesis of *tert*-butyl 3-(*p*-tolylsulfinyl)propanoate (4a) [10]

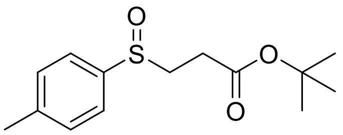

A solution of *tert*-butyl 3-(*p*-tolylthio)propanoate (**3a**, 832.65 mg, 1.52 mmol) in 5 mL of dry dichloromethane, at 0 °C, was treated dropwise with a previously prepared solution of 75% *m*-CPBA (340.65 mg, 1.52 mmol) in 5.3 mL of dry dichloromethane. After sitting for 1 hour at 0 °C, the mixture was washed with a saturated aqueous solution of Na_2SO_3 (2 mL), triethylamine (2 mL) and water (8 mL). The recovered organic phase was dried over anhydrous Na_2SO_4 and evaporated under reduced pressure. Purification by flash chromatography (hexane/diethyl ether (95% to 75% hexane)) gave *tert*-butyl 3-(*p*-tolylsulfinyl)propanoate (**4a**) as a yellow oil in 60% yield.

The product is already reported and spectral data are in accordance with literature values[8].

IR(ATR): 2985 (w), 1727 (s), 1349 (m), 1244 (m), 1152 (s), 1037 (s), 812 (m), 773 (s), 529 (m).

^{1}H NMR (400 MHz, CDCl$_3$): δ$_H$ 7.50 (d, *J* = 8.3 Hz, 2H), 7.32 (d, *J* = 7.9 Hz, 2H), 3.13 (ddd, *J* = 13.5, 8.6, 6.7 Hz, 1H), 2.91 (ddd, *J* = 13.4, 8.5, 5.8 Hz, 1H), 2.72 (ddd, *J* = 17.2, 8.5, 6.7 Hz, 1H), 2.47 – 2.42 (m, 1H), 2.41 (s, 3H), 1.41 (s, 9H).

^{13}C NMR (101 MHz, CDCl$_3$): δ$_C$ 170.5, 141.8, 139.9, 130.1, 124.2, 81.6, 77.5, 77.2, 76.8, 51.6, 28.1, 27.6, 21.5.

3.10. Synthesis of *tert*-butyl 3-(pyridine-2-ylsulfinyl)propanoate (4b) [8]

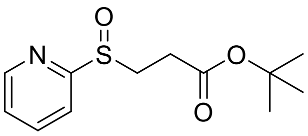

A solution of *tert*-butyl 3-(pyridine-2-ylthio)propanoate (**3b**, 818.52 mg, 3.42 mmol) in 5 mL of dry dichloromethane was combined with wet neutral alumina oxide (4 g, ratio alumina oxide/water = 5 g for 1 mL). Afterward, a solution of Oxone in dichloromethane was added to the mixture at room temperature, and the reaction was refluxed at 40 °C overnight. The resulting solution was then cooled to room temperature, filtered, and evaporated under reduced pressure. The crude residue was purified by flash chromatography (100% hexane to hexane/ethyl acetate 7:3) to give *tert*-butyl 3-(pyridine-2-ylsulfinyl)propanoate (**4b**) as a yellow oil in 32% yield.

The product is already reported and spectral data are in accordance with literature values[9].

IR(ATR): 2981 (w), 1727 (m), 1579 (w), 1368 (m), 1317 (m), 1153 (m), 1112 (m), 812 (m).

^{1}H NMR (400 MHz, $CDCl_3$): δ_H 8.63 (dt, *J* = 4.9, 1.3 Hz, 1H), 7.97 (dt, *J* = 7.8, 1.4 Hz, 1H), 7.93 (td, *J* = 7.5, 1.6 Hz, 1H), 7.38 (ddd, *J* = 7.2, 4.8, 1.6 Hz, 1H), 3.45 (ddd, *J* = 13.6, 9.6, 6.0 Hz, 1H), 3.13 (ddd, *J* = 13.6, 9.4, 5.7 Hz, 1H), 2.79 (ddd, *J* = 16.9, 9.4, 6.0 Hz, 1H), 2.34 (ddd, *J* = 16.9, 9.6, 5.8 Hz, 1H), 1.41 (s, 9H).

^{13}C NMR (101 MHz, $CDCl_3$): δ_C 170.4, 164.1, 149.9, 138.0, 124.8, 120.3, 81.6, 48.5, 28.2, 27.0.

3.11. Synthesis of *tert*-butyl 3-(benzylsulfinyl)propanoate (4c) [10]

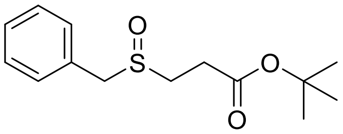

A solution of *tert*-butyl 3-(benzylthio)propanoate (**3c**, 790.00 mg, 3.13 mmol) in 5 mL of dry dichloromethane, at 0 °C, was treated dropwise with a previously prepared solution of 75% *m*-CPBA (710.41 mg, 3.17 mmol) in 11 mL of dry dichloromethane. After sitting for 1 hour at 0 °C, the mixture was washed with a saturated aqueous solution of Na_2SO_3 (2 mL),

triethylamine (2 mL) and water (8 mL). The recovered organic phase was dried over anhydrous Na_2SO_4 and evaporated under reduced pressure. Purification by silica gel filtration (diethyl ether/hexane 10%) gave *tert*-butyl 3-(benzylsulfinyl)propanoate (**4c**) as a yellow oil in 49% yield.

The product is already reported and spectral data are in accordance with literature values[8].

IR(ATR): 2925 (w), 1727 (m), 1495 (m), 1364 (m), 1236 (m), 1156 (m), 1037 (m), 1027 (m), 774 (m).

^{1}H NMR (400 MHz, $CDCl_3$): δ_H 7.41 – 7.28 (m, 5H), 4.07 – 3.92 (m, 2H), 2.97 – 2.86 (m, 1H), 2.79 – 2.66 (m, 3H), 1.43 (s, 9H).

^{13}C NMR (101 MHz, $CDCl_3$): δ_C 170.6, 130.2, 129.8, 129.2, 128.6, 81.7, 58.8, 45.9, 28.2, 28.2.

3.12. Synthesis of *tert*-butyl 3-((4-methoxyphenyl)sulfinyl)propanoate (4d) [10]

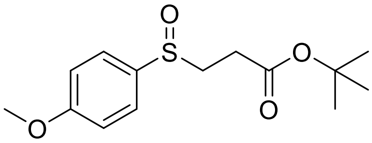

A solution of *tert*-butyl 3-(4-methoxyphenylthio)propanoate (**3d**, 543.46 mg, 2.03 mmol) in 5.1 mL of dry dichloromethane, at 0 °C, was treated dropwise with a previously prepared solution of 75% *m*-CPBA (465.93 mg, 2.03 mmol) in 7.1 mL of dry dichloromethane. After sitting for 1 hour at 0 °C, the mixture was washed with a saturated aqueous solution of Na_2SO_3 (2 mL), triethylamine (2 mL) and water (8 mL). The recovered organic phase was dried over anhydrous Na_2SO_4 and evaporated under reduced pressure. Purification by flash chromatography (100% hexane to ethyl acetate/hexane 2:1) gave *tert*-butyl 3-((4-methoxyphenyl)sulfinyl)propanoate (**4d**) as a yellow oil in 78% yield.

The product is already reported and spectral data are in accordance with literature values[10].

IR(ATR): 3004 (w), 2978 (w), 2933 (w), 1724 (m), 1578 (m), 1497 (m), 1459 (m), 1410 (w), 1248 (s), 1153 (s), 1088 (m), 1026 (s).

^{1}H NMR (400 MHz, CDCl$_3$): δ$_H$ 7.59 – 7.50 (m, 2H), 7.06 – 6.99 (m, 2H), 3.85 (s, 3H), 3.10 (dt, *J* = 14.5, 7.6 Hz, 1H), 2.93 (p, *J* = 7.6 Hz, 1H), 2.69 (q, *J* = 8.8 Hz, 1H), 2.44 (dt, *J* = 15.7, 8.0 Hz, 1H), 1.42 (s, 9H).

^{13}C NMR (101 MHz, CDCl$_3$): δ$_C$ 170.5, 162.2, 134.1, 126.1, 115.0, 81.6, 55.7, 51.8, 28.2, 27.7.

3.13. Synthesis of *tert*-butyl 3-(phenylsulfinyl)propanoate (4e) [10]

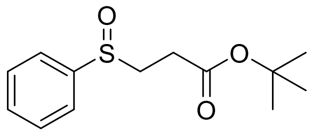

A solution of *tert*-butyl 3-(phenylsulfanyl)propanoate (**3e**, 558.20 mg, 2.34 mmol) in 5 mL of dry dichloromethane, at 0 °C, was treated dropwise with a previously prepared solution of 75% *m*-CPBA (541.60 mg, 2.03 mmol) in 7 mL of dry dichloromethane. After sitting for 1 hour at 0 °C, the mixture was washed with a saturated aqueous solution of Na_2SO_3 (2 mL), triethylamine (2 mL) and water (8 mL). The recovered organic phase was dried over anhydrous Na_2SO_4 and evaporated under reduced pressure. Purification by flash chromatography (100% hexane to ethyl acetate/hexane 7:3) gave *tert*-butyl 3-(phenylsulfinyl)propanoate (**4e**) as a yellow oil in 74% yield.

The product is already reported and spectral data are in accordance with literature values[9].

IR(ATR): 3450 (b), 3057 (w), 2979 (m), 2933 (w), 1727 (s), 1476 (w), 1444 (w), 1366 (m), 1249 (m), 1158 (m), 1047 (m), 751 (m), 693 (m).

^{1}H NMR (400 MHz, CDCl$_3$): δ$_H$ 7.65 – 7.60 (m, 2H), 7.56 – 7.47 (m, 3H), 3.17 (ddd, *J* = 13.4, 8.6, 6.7 Hz, 1H), 2.93 (ddd, *J* = 13.4, 8.5, 5.7 Hz, 1H), 2.76 (ddd, *J* = 17.2, 8.5, 6.7 Hz, 1H), 2.44 (ddd, *J* = 17.1, 8.6, 5.7 Hz, 1H), 1.42 (s, 9H).

^{13}C NMR (101 MHz, CDCl$_3$): δ$_C$ 170.5, 143.3, 131.2, 129.4, 124.2, 81.7, 51.7, 28.2, 27.5.

4. General procedure of electrophilic amination of sulfenate salts

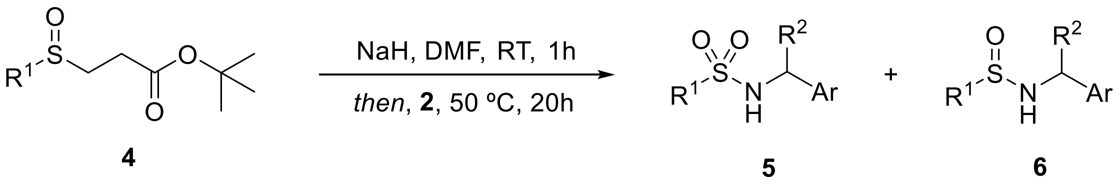

A round-bottom flask was charged with the β-sulfinyl ester **4** (2 equiv) and sodium hydride (2.4 equiv) dissolved in degassed dimethylformamide (0.06 M). The mixture was stirred at room temperature until full conversion of the ester (monitored by TLC). Next, the hypervalent iodine reagent bearing the amine moiety **2** (1 equiv) was added and the resulting mixture was stirred at 50 °C for 20 hours. Afterward, the resulting solution was washed with water, and the recovered organic phase was dried over anhydrous Na_2SO_4 and evaporated under reduced pressure.

4.1. Synthesis of *N*-benzyl-*p*-toluenesulfonamide (5aa)

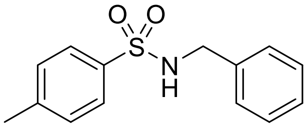

Prepared according to the general procedure using **4a** (61.73 mg, 0.23 mmol), sodium hydride (6.34 mg, 2.4 equiv), hypervalent iodine reagent **2a** (38.85 mg, 0.11 mmol, 1 equiv) and 2 mL of degassed DMF, at 50 °C. Purification by flash chromatography (100% hexane to hexane/ethyl acetate 4:1) gave *N*-benzyl-*p*-toluenesulfonamide (**5aa**) in 52% yield.

The product is already reported and spectral data are in accordance with literature values[11].

m.p.: 111 °C.

IR(ATR): 3271 (w), 1730 (m), 1598 (m), 1455 (m), 1320 (m), 1157 (s), 1089 (m), 814 (m), 698 (m), 662 (m), 552 (m).

^{1}H NMR (400 MHz, CDCl$_3$): δ_H 7.79 – 7.71 (m, 2H), 7.29 (d, *J* = 8.1 Hz, 2H), 7.25 – 7.15 (m, 5H), 4.62 (br. t, *J* = 6.4 Hz, 1H), 4.10 (d, *J* = 6.2 Hz, 2H), 2.42 (s, 3H).

^{13}C NMR (101 MHz, CDCl$_3$): δ$_C$ 170.6, 141.7, 140.0, 130.1, 128.4, 124.2, 82.0, 81.6, 51.8, 51.7, 29.2, 28.2, 28.1, 27.6, 21.6.

4.2. Synthesis of *N*-(4-methylbenzyl)-*p*-toluenesulfonamide (5ab)

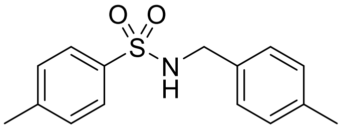

Prepared according to the general procedure using **4a** (61.76 mg, 0.23 mmol), sodium hydride (6.30 mg, 2.4 equiv), hypervalent iodine reagent **2b** (40.39 mg, 0.11 mmol, 1 equiv) and 2 mL of degassed DMF, at 50 °C. Purification by flash chromatography (100% hexane to hexane/ethyl acetate 4:1) gave *N*-(4-methylbenzyl)-*p*-toluenesulfonamide (**5ab**) in 48% yield. The product is already reported and spectral data are in accordance with literature values[12].

m.p.: 89-91 ºC.

IR(ATR): 3267 (m), 2920 (m), 1598 (m), 1426 (m), 1324 (s), 1310 (s), 1153 (w), 1093 (m), 1065 (m), 813 (s), 663 (s), 550 (s).

^{1}H NMR (400 MHz, CDCl$_3$): δ$_H$ 7.76 (d, *J* = 8.2 Hz, 2H), 7.30 (d, *J* = 7.9 Hz, 2H), 7.08 (s, 4H), 4.68 (br. t, *J* = 6.2 Hz, 1H), 4.07 (d, *J* = 6.1 Hz, 2H), 2.44 (s, 3H), 2.31 (s, 3H).

^{13}C NMR (101 MHz, CDCl$_3$): δ$_C$ 143.6, 137.8, 137.0, 133.3, 129.9, 129.5, 128.0, 127.3, 47.2, 21.7, 21.2.

4.3. Synthesis of (*S*)-4-methyl-*N*-(1-phenylethyl)benzenesulfonamide (5ac)

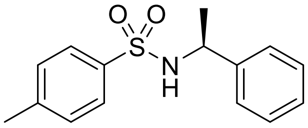

Prepared according to the general procedure using **4a** (61.82 mg, 0.23 mmol), sodium hydride (6.41 mg, 2.4 equiv), hypervalent iodine reagent **2c** (40.47 mg, 0.11 mmol, 1 equiv) and 2 mL of degassed DMF, at 50 °C. Purification by flash chromatography (100% hexane to

hexane/ethyl acetate 4:1) gave (*S*)-4-methyl-*N*-(1-phenylethyl)benzenesulfonamide (**5ac**) in 43% yield.

The product is already reported and spectral data are in accordance with literature values[13].

m.p.: 96-98 ºC.

IR(ATR): 3250 (m), 1435 (m), 1318 (m), 1159 (s), 1088 (m), 1013 (m), 810 (m), 676 (m), 537 (m).

^{1}H NMR (400 MHz, CDCl$_3$): δ_H 7.62 (d, *J* = 8.3 Hz, 2H), 7.23 – 7.07 (m, 7H), 4.95 (br. d, *J* = 6.9 Hz, 1H), 4.46 (p, *J* = 6.9 Hz, 1H), 2.38 (s, 3H), 1.42 (d, *J* = 6.8 Hz, 3H).

^{13}C NMR (101 MHz, CDCl$_3$): δ_C 143.3, 142.2, 137.8, 129.6, 128.7, 127.6, 127.2, 126.2, 53.8, 23.7, 21.6.

4.4. Synthesis of *N*-(4-fluorobenzyl)-*p*-toluenesulfonamide (5ad)

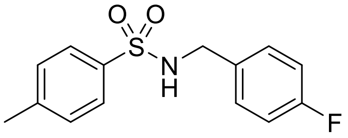

Prepared according to the general procedure using **4a** (58.90 mg, 0.22 mmol), sodium hydride (6.40 mg, 2.4 equiv), hypervalent iodine reagent **2d** (41.30 mg, 0.11 mmol, 1 equiv) and 2 mL of degassed DMF, at 50 °C. Purification by flash chromatography (100% hexane to hexane/ethyl acetate 2:1) gave *N*-(4-fluorobenzyl)-*p*-toluenesulfonamide (**5ad**) in 25% yield.

The product is already reported and spectral data are in accordance with literature values[14].

IR(ATR): 3276 (m), 3072 (w), 2925 (w), 1509 (m), 1438 (m), 1326 (m), 1222 (m), 1156 (s), 863 (w).

^{1}H NMR (400 MHz, CDCl$_3$): δ_H 7.74 (d, *J* = 8.3 Hz, 2H), 7.31 (d, *J* = 8.0 Hz, 2H), 7.17 (dd, *J* = 8.5, 5.5 Hz, 2H), 6.96 (t, *J* = 8.6 Hz, 2H), 4.70 (br. t, *J* = 6.3 Hz, 1H), 4.09 (d, *J* = 6.2 Hz, 2H), 2.44 (s, 3H).

^{13}C NMR (101 MHz, CDCl$_3$): δ_C 162.5 (d, *J* = 246.7 Hz), 143.8, 137.0, 132.2 (d, *J* = 3.3 Hz), 129.9, 129.8 (d, *J* = 8.2 Hz), 127.3, 115.7 (d, *J* = 21.6 Hz), 46.7, 21.7.

^{19}F NMR (376 MHz, CDCl$_3$): δ_F -114.2.

4.5. Synthesis of *N*-benzylpyridine-2-sulfonamide (5ba)

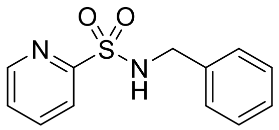

Prepared according to the general procedure using **4b** (58.73 mg, 0.23 mmol), sodium hydride (6.62 mg, 2.4 equiv), hypervalent iodine reagent **2a** (39.16 mg, 0.11 mmol, 1 equiv) and 2 mL of degassed DMF, at 50 °C. Purification by flash chromatography (100% hexane to hexane/ethyl acetate 4:1) gave *N*-benzylpyridine-2-sulfonamide (**5ba**) in 32% yield.

The product is already reported and spectral data are in accordance with literature values[15].

m.p.: 103 ºC.

IR(ATR): 2923 (m), 1731 (s), 1642 (m), 1533 (m), 1454 (m), 1301 (m), 1079 (m), 1016 (m), 697 (m).

^{1}H NMR (400 MHz, CDCl$_3$): δ_H 7.90 – 7.83 (m, 1H), 7.45 – 7.27 (m, 7H), 7.14 – 7.05 (m, 1H), 6.01 (br. s, 1H), 4.66 (d, *J* = 5.7 Hz, 2H).

^{13}C NMR (101 MHz, CDCl$_3$): δ_C 142.2, 140.1, 137.7, 131.3, 128.9, 128.5, 128.3, 127.9, 92.6, 44.4.

4.6. Synthesis of *N*-benzyl-4-methoxybenzenesulfonamide (5da)

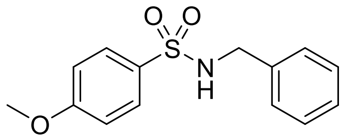

Prepared according to the general procedure using **4d** (62.70 mg, 0.22 mmol), sodium hydride (6.30 mg, 2.4 equiv), hypervalent iodine reagent **2a** (38.80 mg, 0.11 mmol, 1 equiv) and 2 mL of degassed DMF, at 50 °C. Purification by preparative thin layer chromatography

(pTLC) (hexane/ethyl acetate 4:1), followed by another pTLC (cyclohexane/ethyl acetate 4:1) gave *N*-benzyl-4-methoxybenzenesulfonamide (**5da**) in 26% yield.

The product is already reported and spectral data are in accordance with literature values[9].

IR(ATR): 3275 (b), 3067 (w), 3031 (w), 3008 (w), 2940 (w), 1597 (m), 1581 (m), 1496 (m), 1457 (m), 1324 (m), 1301 (m), 1259 (m), 1151 (s), 1096 (m), 695 (m), 559 (m).

^{1}H NMR (400 MHz, $CDCl_3$): δ_H 7.80 (d, *J* = 8.9 Hz, 2H), 7.31 – 7.22 (m, 3H), 7.19 (dd, *J* = 7.5, 2.0 Hz, 2H), 6.97 (d, *J* = 9.0 Hz, 2H), 4.61 (t, *J* = 6.0 Hz, 1H), 4.11 (d, *J* = 6.2 Hz, 2H), 3.87 (s, 3H).

^{13}C NMR (101 MHz, $CDCl_3$): δ_C 163.1, 136.4, 131.6, 129.5, 128.9, 128.1, 128.0, 114.4, 55.8, 47.4.

4.7. Synthesis of *N*-(4-fluorobenzyl)-4-methoxybenzenesulfonamide (5dd)

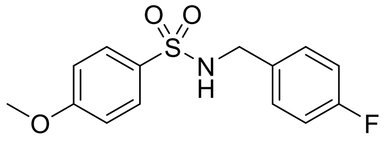

Prepared according to the general procedure using **4d** (62.56 mg, 0.22 mmol), sodium hydride (6.34 mg, 2.4 equiv), hypervalent iodine reagent **2d** (40.80 mg, 0.11 mmol, 1 equiv) and 2 mL of degassed DMF, at 50 °C. Purification by flash chromatography (100% hexane to hexane/ethyl acetate 1:1) gave *N*-(4-fluorobenzyl)-4-methoxybenzenesulfonamide (**5dd**) in 28% yield.

Rf: 0.12 (hexane/ethyl acetate 4:1).

IR(ATR): 3271 (w), 2927 (w), 1510 (m), 1498 (m), 1462 (w), 1442 (w), 1258 (m), 1223 (m), 1182 (w), 1150 (s), 1094 (m), 702 (m).

^{1}H NMR (400 MHz, $CDCl_3$): δ_H 7.79 (d, *J* = 8.9 Hz, 2H), 7.17 (dd, *J* = 8.5, 5.4 Hz, 2H), 6.99 – 6.96 (m, 2H), 6.96 – 6.93 (m, 2H), 4.64 (br. t, *J* = 6.2 Hz, 1H), 4.09 (d, *J* = 6.3 Hz, 2H), 3.88 (s, 3H).

^{13}C NMR (101 MHz, $CDCl_3$): δ_C 163.2, 162.5 (d, *J* = 246.7 Hz), 132.3 (d, *J* = 3.3 Hz), 131.5, 129.8 (d, *J* = 8.3 Hz), 129.4, 115.7 (d, *J* = 21.6 Hz), 114.5, 55.8, 46.7.

^{19}F NMR (376 MHz, CDCl$_3$): δ_F -114.2.

HRMS (ESI$^+$) Calculated for $C_{14}H_{15}FNO_3$ [M+H]$^+$: 296.0751, found 296.0746.

4.8. Synthesis of *N*-benzylbenzenesulfonamide (5ea)

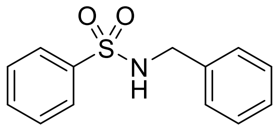

Prepared according to the general procedure using **4e** (58.50, 0.23 mmol), sodium hydride (10.56 mg, 2.4 equiv), hypervalent iodine reagent **2a** (38.85 mg, 0.11 mmol, 1 equiv) and 2 mL of degassed DMF, at 50 °C. Purification by pTLC (cyclohexane/ethyl acetate 4:1) gave *N*-benzylbenzenesulfonamide (**5ea**) in 39% yield.

The product is already reported and spectral data are in accordance with literature values[16].

^{1}H NMR (400 MHz, CDCl$_3$): δ_H 7.88 (d, J = 7.7 Hz, 2H), 7.59 (t, J = 7.1 Hz, 1H), 7.51 (t, J = 7.5 Hz, 2H), 7.32 – 7.22 (m, 3H), 7.20 (d, J = 6.8 Hz, 2H), 5.04 (br. s, 1H), 4.15 (d, J = 6.0 Hz, 2H).

^{13}C NMR (101 MHz, CDCl$_3$): δ_C 140.0, 136.3, 132.8, 129.2, 128.7, 127.9, 127.2, 47.3.

5. NMR Studies to Investigate the formation of 3 and 7

Pathway B (Scheme 6 of the manuscript) was further investigated. Additional experiments were carried out in the absence of BBX **2**, using as starting material the β-sulfinyl ester **4**. These reactions were monitored by ^{1}H NMR of crude residues, in order to verify the formation of the products **3**, **4** and **7**.

5.1. Experiment in the absence of BBX 2

A flame-dried Schlenk flask was charged with the β-sulfinyl ester **4e** (75.10 mg, 0.30 mmol) and sodium hydride (8.70 mg, 0.36 mmol) dissolved in 2.6 mL of degassed dimethylformamide. The reaction mixture was stirred at room and, after 1 h, part of the reactional mixture was removed for analysis (work-up and ^{1}H NMR analysis). Next, the resulting mixture was stirred at 60 °C for 21 h. Afterward, the resulting solution was washed with water, and the recovered organic phase was dried over anhydrous Na_2SO_4 and evaporated under reduced pressure.

The overlap of the ^{1}H NMR spectra of the 1 h and 21 h aliquots with the spectrum of the starting material **4e** (Figure S1) revealed that, after 1 h at room temperature, the regeneration of compound **4e** is observed, but new peaks appeared in the aromatic region (around 7.30 ppm). The crude mixture recovered after 21 h at 60 °C revealed an increase in the peaks around 7.30 ppm, the formation of new signals in the aliphatic region (disappearance of the 4 doublets of doublets of doublets, and appearance of 2 triplets) and a shift in the signal of the *tert*-butyl group protons to downfield (from 1.42 ppm to 1.45 ppm). This information led to the overlap of the spectra with compound **3e** (Figure S2), which led to the conclusion that thioether **3** is formed during the reaction.

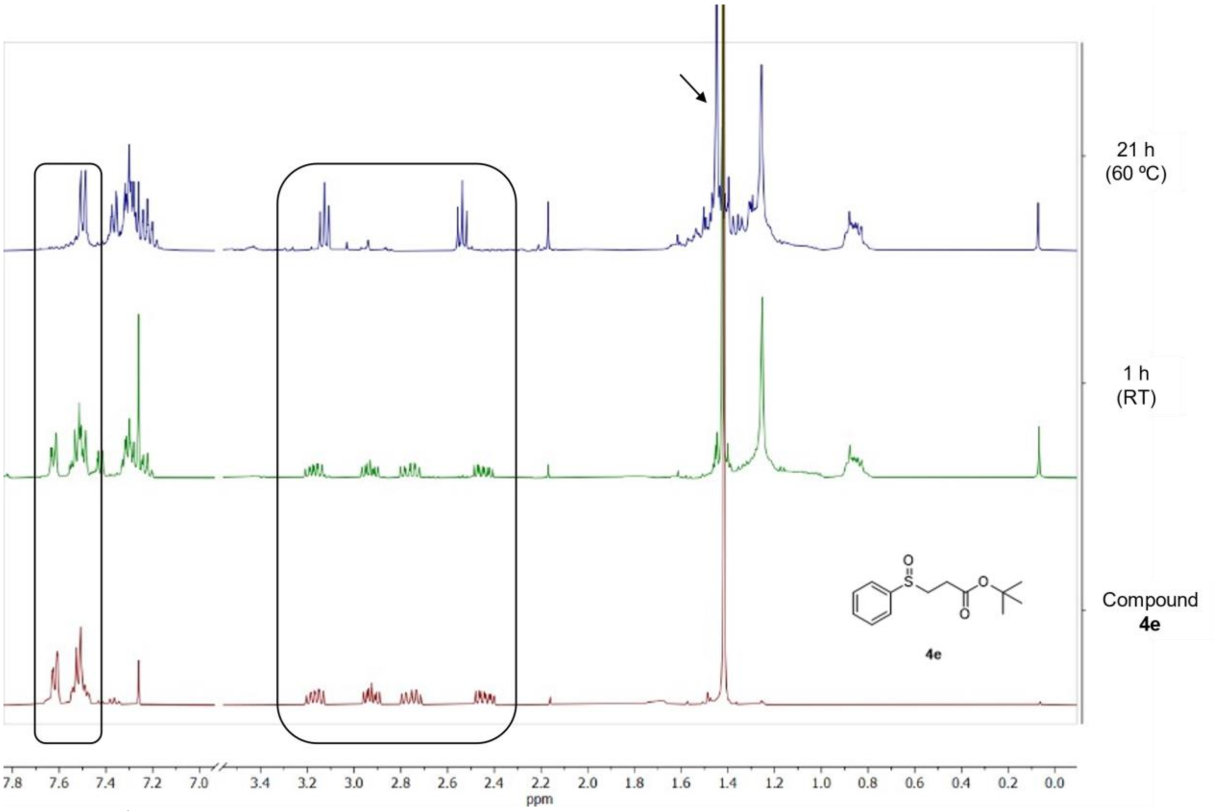

Figure S1. *^{1}H NMR spectrum (CDCl₃, 400 MHz) of crude residues of the reaction without compound **2**, after 1 h (green) and after 21 h (blue), superimposed with starting material **4e** spectrum (red).*

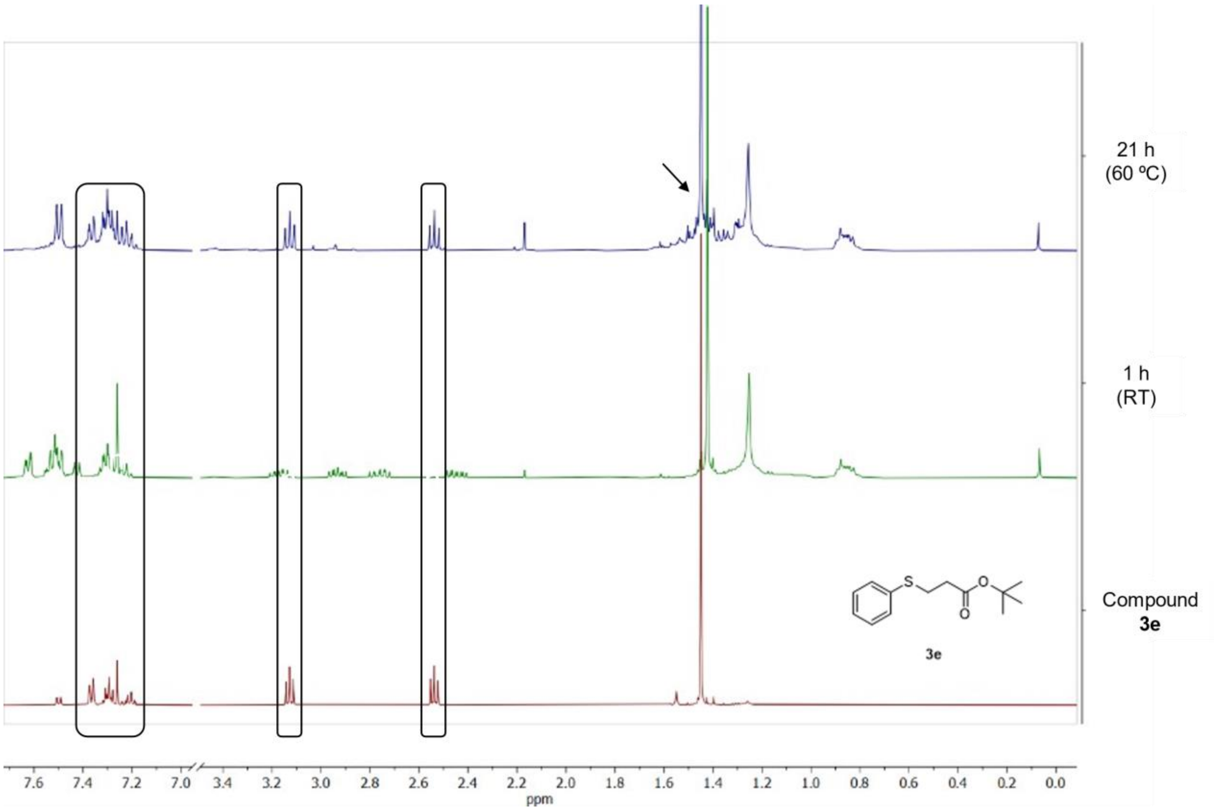

Figure S2. *^{1}H NMR spectrum (CDCl₃, 400 MHz) of crude residues of the reaction without compound **2**, after 1 h (green) and after 21 h (blue), superimposed with compound **3e** spectrum (red).*

5.2. Experiment in the absence of BBX 2 and light

A flame-dried Schlenk flask was charged with the β-sulfinyl ester **4e** (61.20 mg, 0.24 mmol) and sodium hydride (7.20 mg, 0.30 mmol) dissolved in 2.1 mL of degassed dimethylformamide. The mixture was stirred in the dark at room temperature and, after 1 h, part of the reaction mixture was removed for analysis (work-up and ^{1}H NMR analysis). Next, the resulting mixture was stirred at 60 °C for 21 h (still in the dark). Afterward, the resulting solution was washed with water, and the recovered organic phase was dried over anhydrous Na_2SO_4 and evaporated under reduced pressure.

After 1 h at room temperature and in the dark, similar results to the reaction performed with light were obtained: regeneration of compound **4e** and the appearance of new signals in the aromatic region (around 7.30 ppm) (Figure S3). However, although there was still an increase in the intensity of the signals around 7.30 ppm, the crude mixture recovered after 21 h at 60 °C turned out to be different from the experiment carried out with light, with signals in the aliphatic region that could be associated both to starting material **4e** and thioether **3e** (Figure S3 and Figure S4, respectively).

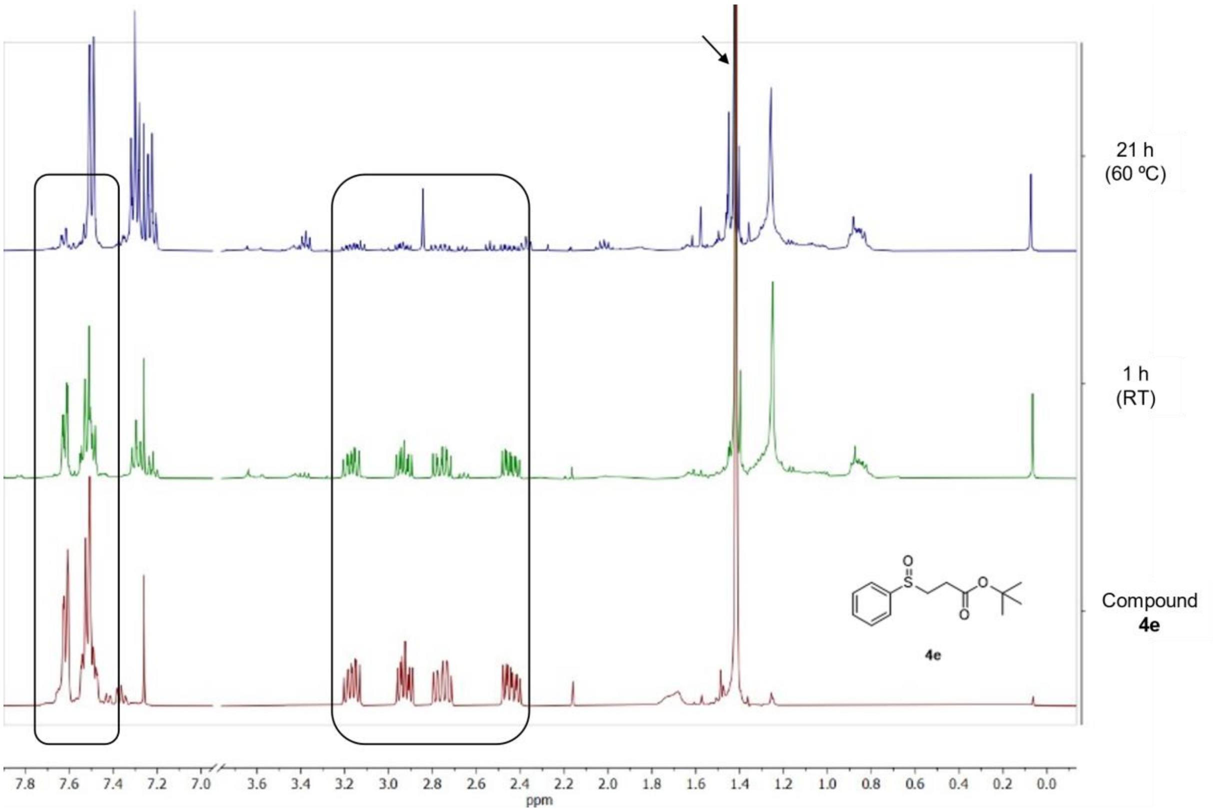

Figure S3. *^{1}H NMR spectrum (CDCl$_3$, 400 MHz) of crude residues of the reaction without compound **2** and in the dark, after 1 h (green) and after 21 h (blue), superimposed with starting material **4e** spectrum (red).*

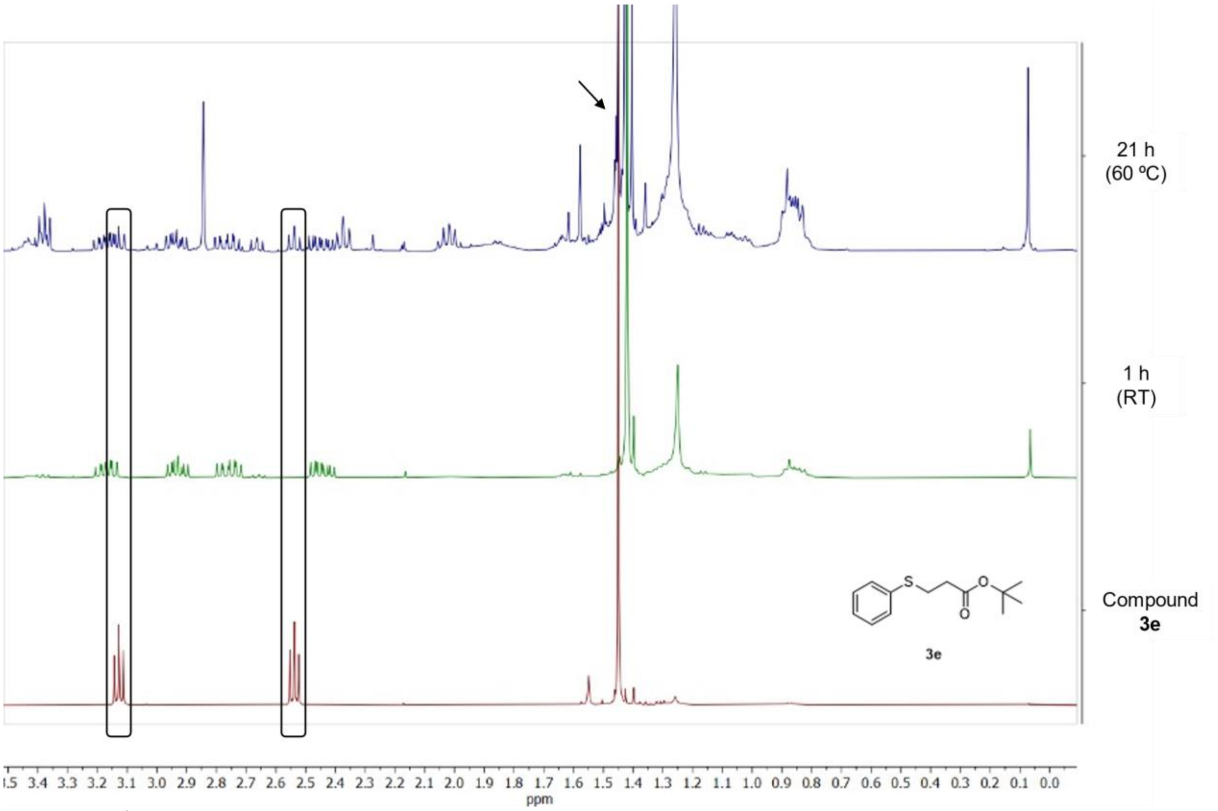

Figure S4. *¹H NMR spectrum expansion ($CDCl_3$, 400 MHz) of crude residues of the reaction without compound* ***2*** *and in the dark, after 1 h (green) and after 21 h (blue), superimposed with compound* ***3e*** *spectrum (red).*

5.3. Experiment in the absence of BBX 2, and with addition of 10 mol % of galvinoxyl

A flame-dried Schlenk flask was charged with the β-sulfinyl ester **4e** (67.10 mg, 0.26 mmol), sodium hydride (7.97 mg, 0.33 mmol) and galvinoxyl (6.00 mg, 0.01 mmol) dissolved in 2.3 mL of degassed dimethylformamide. The mixture was stirred at room temperature and, after 1 h, part of the reaction mixture was removed for analysis (work-up and ^{1}H NMR analysis). Next, the resulting mixture was stirred at 60 °C for 21 h. Afterward, the resulting solution was washed with water, and the recovered organic phase was dried over anhydrous Na_2SO_4 and evaporated under reduced pressure.

After 1 h at room temperature, regeneration of **4e** was once again detected (Figure S5). However, superimposition of the spectrum of the crude mixture at 21 h with that of **3e** (Figure S6) shows that, in the presence of galvinoxyl, **3e** was not detected.

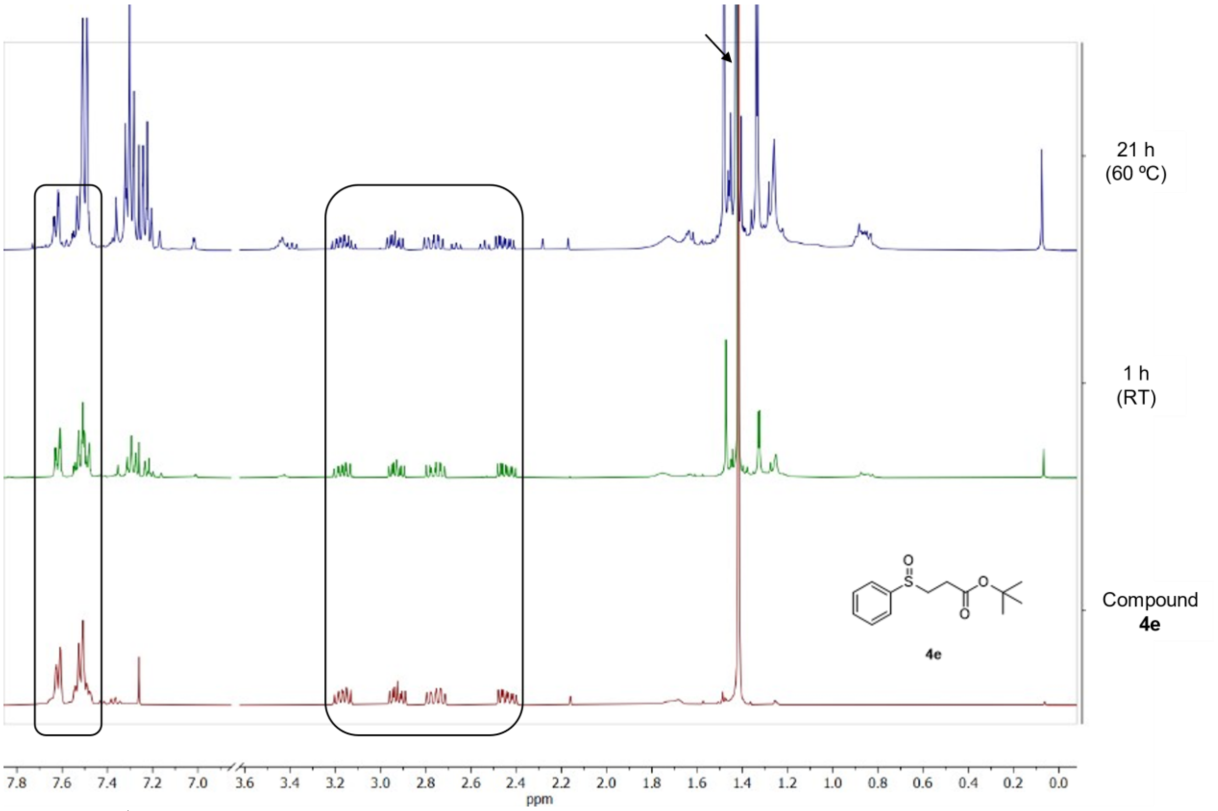

Figure S5. ^{1}H NMR spectrum (CDCl$_3$, 400 MHz) of crude residues of the reaction without compound **2** and with the addition of 10 mol % of galvinoxyl, after 1 h (green) and after 21 h (blue), superimposed with starting material **4e** spectrum (red).

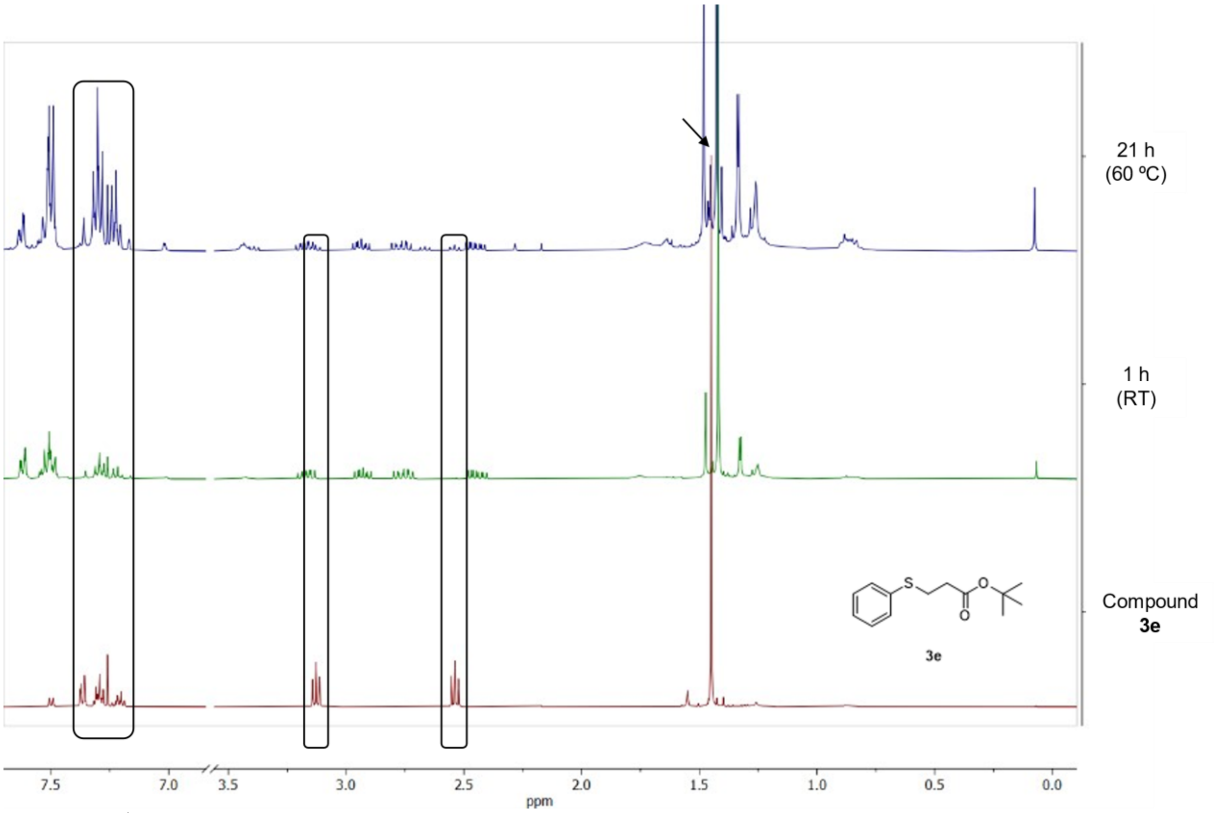

Figure S6. ^{1}H NMR spectrum (CDCl$_3$, 400 MHz) of crude residues of the reaction without compound **2** and with the addition of 10 mol % of galvinoxyl, after 1 h (green) and after 21 h (blue), superimposed with compound **3e** spectrum (red).

5.4. Mechanistic conclusions

No significant changes were found after 1 h in the spectra obtained for the three experiments carried out, confirming that, at room temperature, only the equilibrium of the retro-Michael/Michael reactions occurred (Figure S7).

Analysis of the spectra obtained in the reactions performed in the absence of BBX **2** suggests that a radical mechanism is involved, which is evidenced by the results obtained in the reactions performed in the absence of light and in the presence of a radical scavenger (10 mol % of galvinoxyl), with compound **3e** being detected only in trace amounts (Figure S6). This indicates that light might be the principal initiator of the radical mechanism, although the homolytic cleavage proposed can also be thermo-induced since the reaction is carried out at 60 °C.

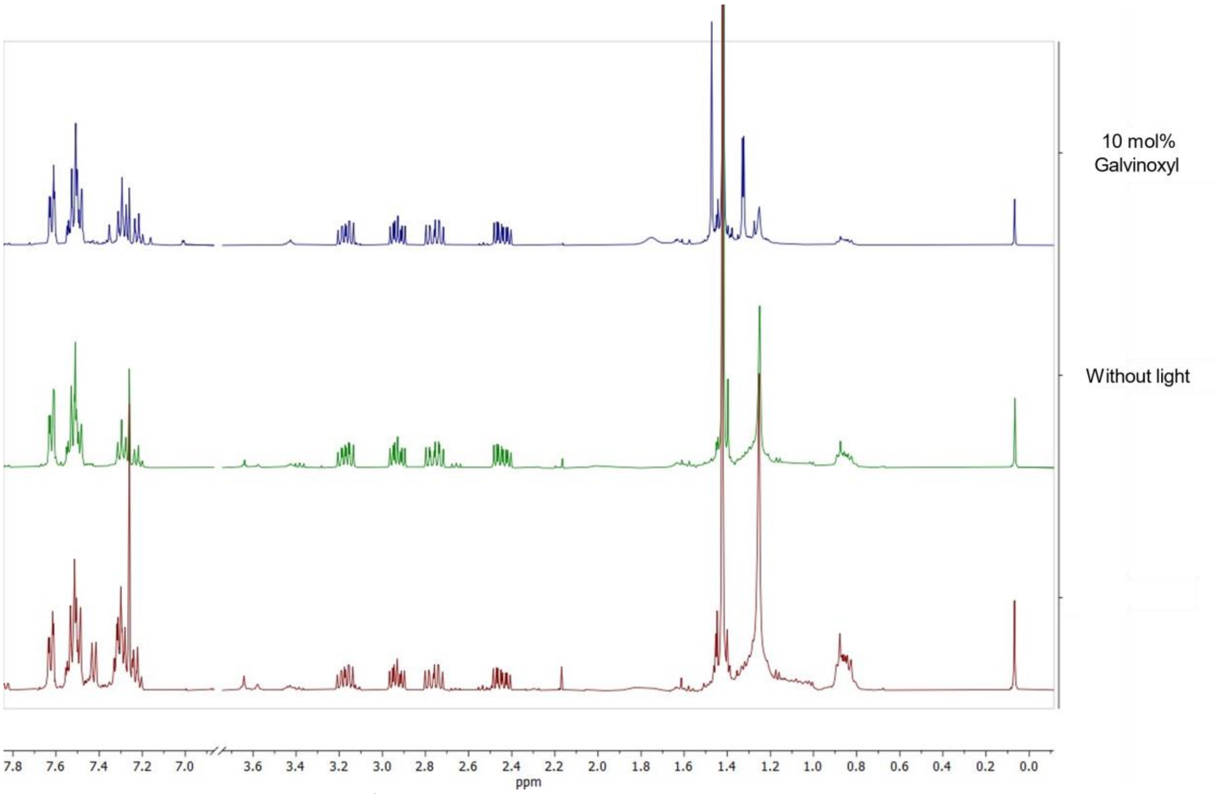

Figure S7. *Comparison between* ^{1}*H NMR spectrum (*$CDCl_3$*, 400 MHz) of crude residues after 1 h. Trial without BBX (**5.1**, red), trial without BBX or light (**5.2**, green), and trial with galvinoxyl (**5.3**, red).*

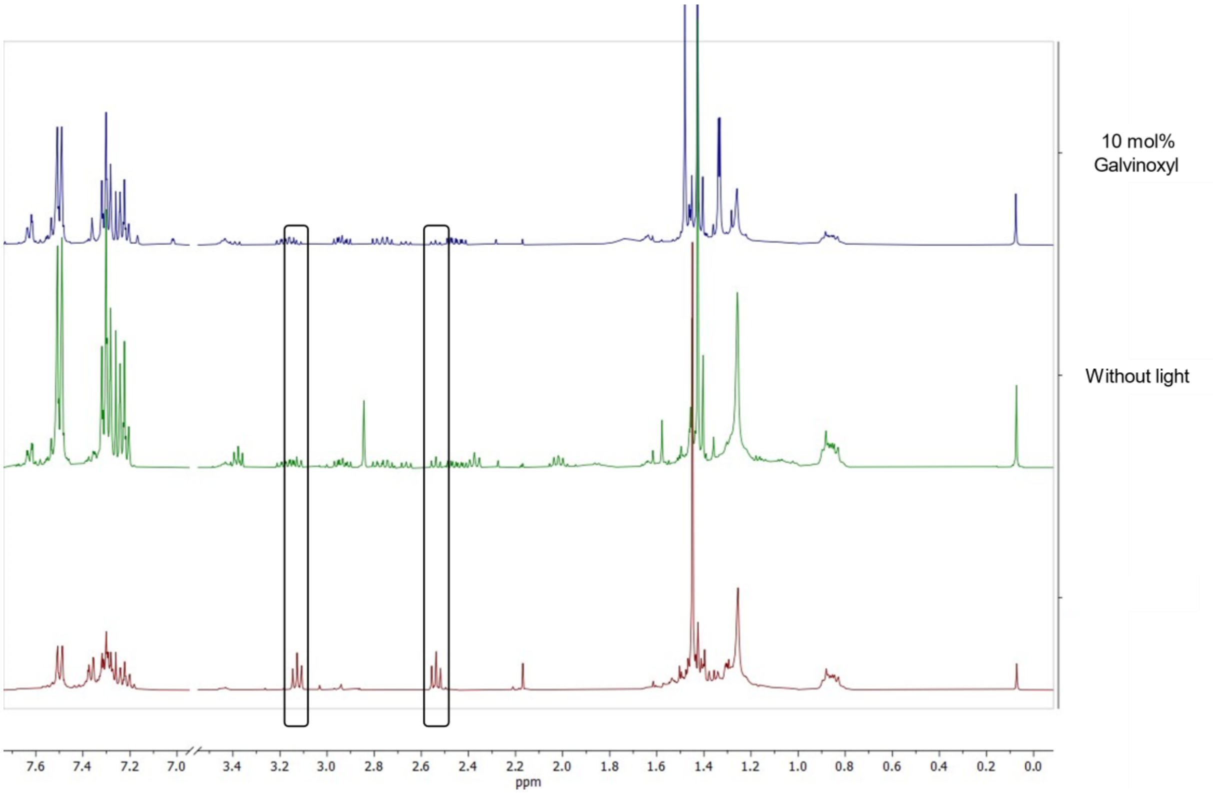

Figure S6. *Comparison between ¹H NMR spectrum (CDCl₃, 400 MHz) of crude residues after 21 h. Trial without BBX (**5.1**, red), trial without BBX or light (**5.2**, green), and trial with galvinoxyl (**5.3**, red).*

6. Single crystal X-ray crystallography study

A crystal of compound **2d** suitable for single-crystal X-ray analysis was selected, covered with Fomblin (polyfluoro ether oil) and mounted on a nylon loop. The data was collected at 293(2) K on a Bruker D8 Venture diffractometer equipped with a Photon II detector and an Oxford CryoSystems Cooler, using graphite monochromated MoKα radiation (λ = 0.71073 Å). The data was processed using the APEX4 suite software package, which includes integration and scaling (SAINT), absorption corrections (SADABS)[17] and space group determination (XPREP). Structure solution and refinement were done using direct methods with the programs SHELXT 2018/2 and SHELXL-2019/2 [18,19] inbuilt in APEX and WinGX-Version 2021.3 [20] software packages. All non-hydrogen atoms were refined anisotropically, and all hydrogen atoms were inserted in idealized positions and allowed to refine riding on the parent carbon atom with C–H distances of 0.97 Å and 0.93 Å for methylene and aromatic H atoms, respectively. The molecular diagram was drawn with ORTEP-3 and Mercury [20,21]. Crystal data for compound **2d**: $C_{28}H_{22}F_2I_2N_2O_5$, FW = 758.27, monoclinic, space group *C2/c* (no.15), 0.26 × 0.12 × 0.08 mm^3 (colorless plate), D_c = 1.823 g cm^{-3}, Z = 4, *a* = 12.4949(6), *b* = 8.1231(6), *c* = 27.3448(16) Å, α = 90°, β = 95.528(3)°, γ = 90°, *V* = 2762.5(3) Å^3, *T* = 293(2) K, Bruker D8 Venture diffractometer with Photon II detector, λ (MoKα) = 0.71073 Å, μ = 2.331 mm^{-1}. Of 24035 reflections measured (R_{int} = 0.1408), 2608 were unique. Refinement on F^2 concluded with the values *R1* = 0.0655 and wR2 = 0.1658 for 175 parameters and 2179 data with $I > 2\sigma I$. The data was deposited in the CCDC under deposit number 2368436.

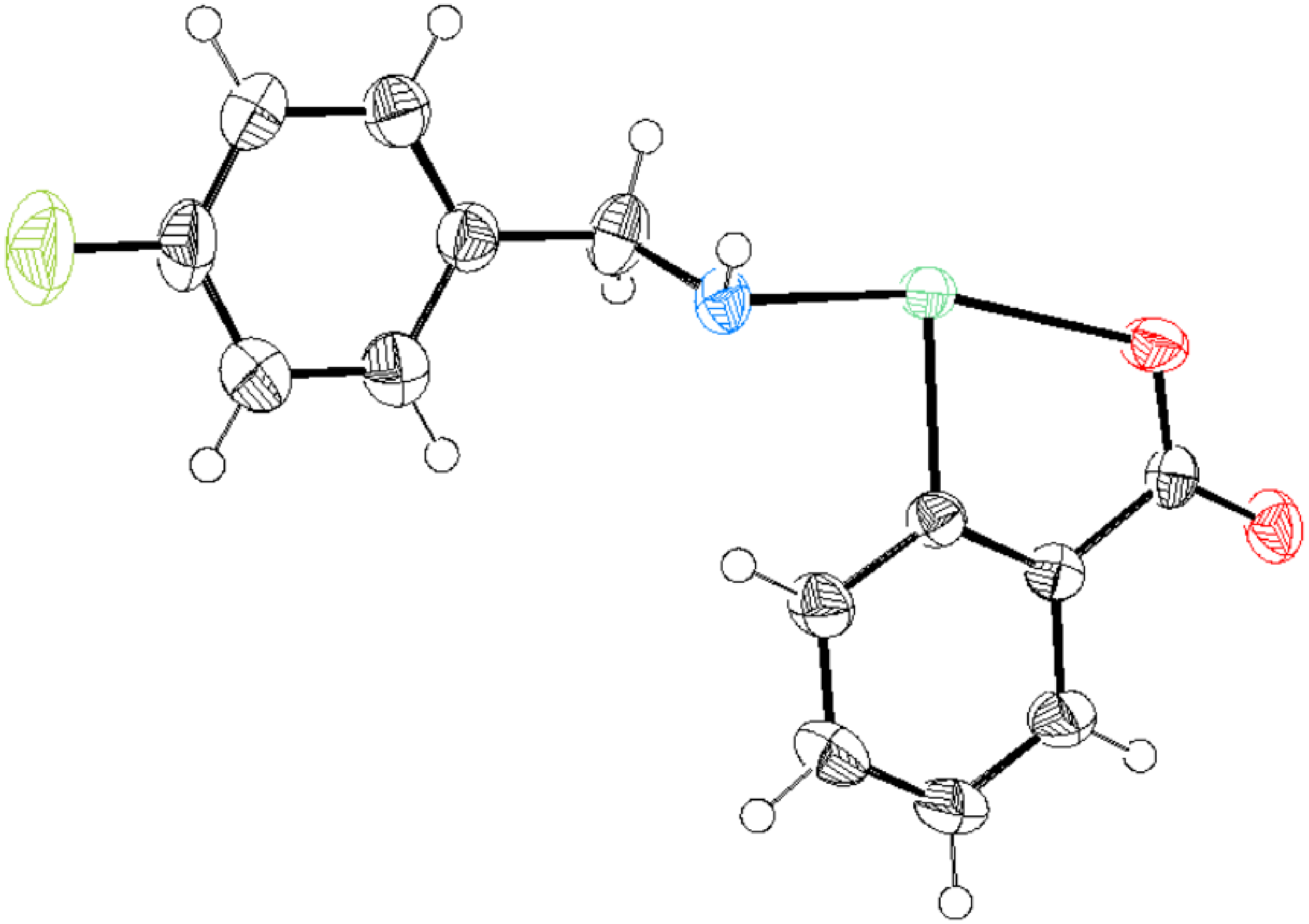

Figure S9. *ORTEP-3 diagram of compound* ***2d*** *(asymmetric unit), using 50% probability level ellipsoids. One co-crystallized water molecule was omitted for clarity.*

7. References

(1) Frei, R.; Courant, T.; Wodrich, M. D.; Waser, J. *Chemistry – A European Journal* **2015**, *21*, 2662–2668. doi:10.1002/CHEM.201406171

(2) Poeira, D. L.; Negrão, A. C. R.; Faustino, H.; Coelho, J. A. S.; Gomes, C. S. B.; Gois, P. M. P.; Marques, M. M. B. *Org Lett* **2022**, *24*, 776–781. doi:10.1021/acs.orglett.1c04312

(3) Zhang, Y.; Lu, J.; Lan, T.; Cheng, S.; Liu, W.; Chen, C. *European J Org Chem* **2021**, *2021*, 436–442. doi:10.1002/ejoc.202001373

(4) Gründler, F.; Scholz, H.; Herbig, M.; Schwarzer, S.; Wagler, J.; Kroke, E. *Eur J Inorg Chem* **2021**, *2021*, 2211–2224. doi:10.1002/ejic.202100118

(5) Hopkins, S. A.; Ritsema, T. A.; Konopelski, J. P. *Journal of Organic Chemistry* **1999**, *64*, 7885–7889. doi:10.1021/jo9909296

(6) Esker, J. L.; Newcomb, M. *Journal of Organic Chemistry* **1994**, *59*, 2779–2786. doi:10.1021/jo00089a023

(7) Xu, M.; Jupp, A. R.; Ong, M. S. E.; Burton, K. I.; Chitnis, S. S.; Stephan, D. W. *Angewandte Chemie* **2019**, *131*, 5763–5767. doi:10.1002/ange.201900058

(8) Maitro, G.; Prestat, G.; Madec, D.; Poli, G. *J Org Chem* **2006**, *71*, 7449–7454. doi:10.1021/jo061359u

(9) Amos, S. G. E.; Nicolai, S.; Gagnebin, A.; Le Vaillant, F.; Waser, J. *J Org Chem* **2019**, *84*, 3687–3701. doi:10.1021/acs.joc.9b00050

(10) Wang, L.; Chen, M.; Zhang, P.; Li, W.; Zhang, J. *J Am Chem Soc* **2018**, *140*, 3467–3473. doi:10.1021/jacs.8b00178

(11) Bhuyan, R.; Nicholas, K. M. *Org Lett* **2007**, *9*, 3957–3959. doi:10.1021/ol701544z

(12) Caddick, S.; Wilden, J. D.; Judd, D. B. *Chemical Communications* **2005**, No. 21, 2727–2728. doi:10.1039/b501212k

(13) Takeuchi, Y.; Satoh, A.; Suzuki, T.; Kameda, A.; Dohrin, M.; Satoh, T.; Koizumi, T.; Kirk, K. L. *Chem Pharm Bull (Tokyo)* **1997**, *45*, 1085–1088. doi:10.1248/cpb.45.1085

(14) Pan, X.; Gao, J.; Liu, J.; Lai, J.; Jiang, H.; Yuan, G. *Green Chemistry* **2015**, *17*, 1400–1403. doi:10.1039/c4gc02115k

(15) Iakovenko, R. O.; Chrenko, D.; Kristek, J.; Desmedt, E.; Zálešák, F.; De Vleeschouwer, F.; Pospíšil, J. *Org Biomol Chem* **2022**, *20*, 3154–3159. doi:10.1039/d2ob00345g

(16) Poeira, D. L.; Macara, J.; Faustino, H.; Coelho, J. A. S.; Gois, P. M. P.; Marques, M. M. B. *European J Org Chem* **2019**, *2019*, 2695–2701. doi:10.1002/ejoc.201900259

(17) Krause, L.; Herbst-Irmer, R.; Sheldrick, G. M.; Stalke, D. *J Appl Crystallogr* **2015**, *48*, 3–10. doi:10.1107/S1600576714022985

(18) Sheldrick, G. M. *Acta Crystallogr C Struct Chem* **2015**, *71*, 3–8. doi:10.1107/S2053229614024218

(19) Hübschle, C. B.; Sheldrick, G. M.; Dittrich, B. *J Appl Crystallogr* **2011**, *44*, 1281–1284. doi:10.1107/S0021889811043202

(20) Farrugia, L. J. *J Appl Crystallogr* **2012**, *45*, 849–854. doi:10.1107/S0021889812029111

(21) Macrae, C. F.; Sovago, I.; Cottrell, S. J.; Galek, P. T. A.; McCabe, P.; Pidcock, E.; Platings, M.; Shields, G. P.; Stevens, J. S.; Towler, M.; Wood, P. A. *J Appl Crystallogr* **2020**, *53*, 226–235. doi:10.1107/S1600576719014092

8. NMR spectra

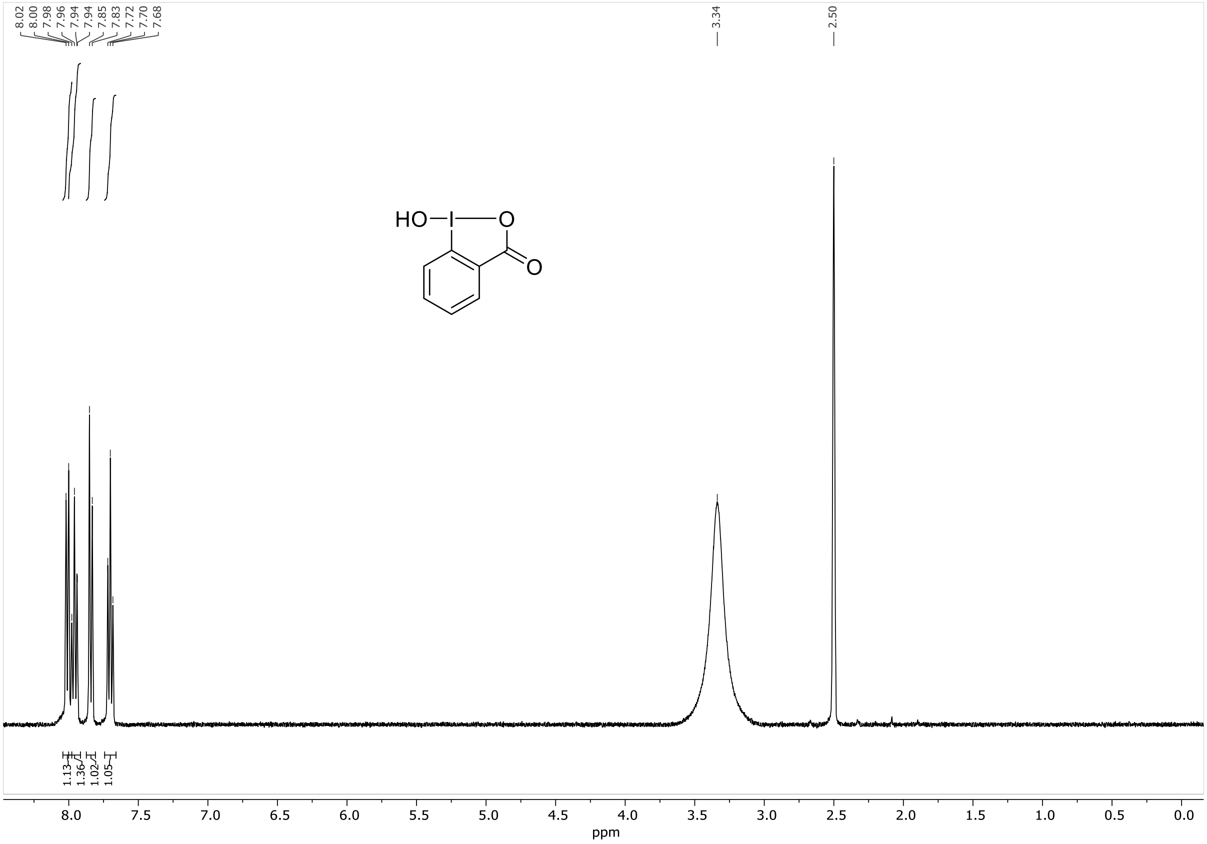

Figure S10. *^{1}H NMR spectrum (DMSO-$_{d6}$, 400 MHz) of 1-hydroxy-1,2-benziodoxol-3-(1H)-one (**1**).*

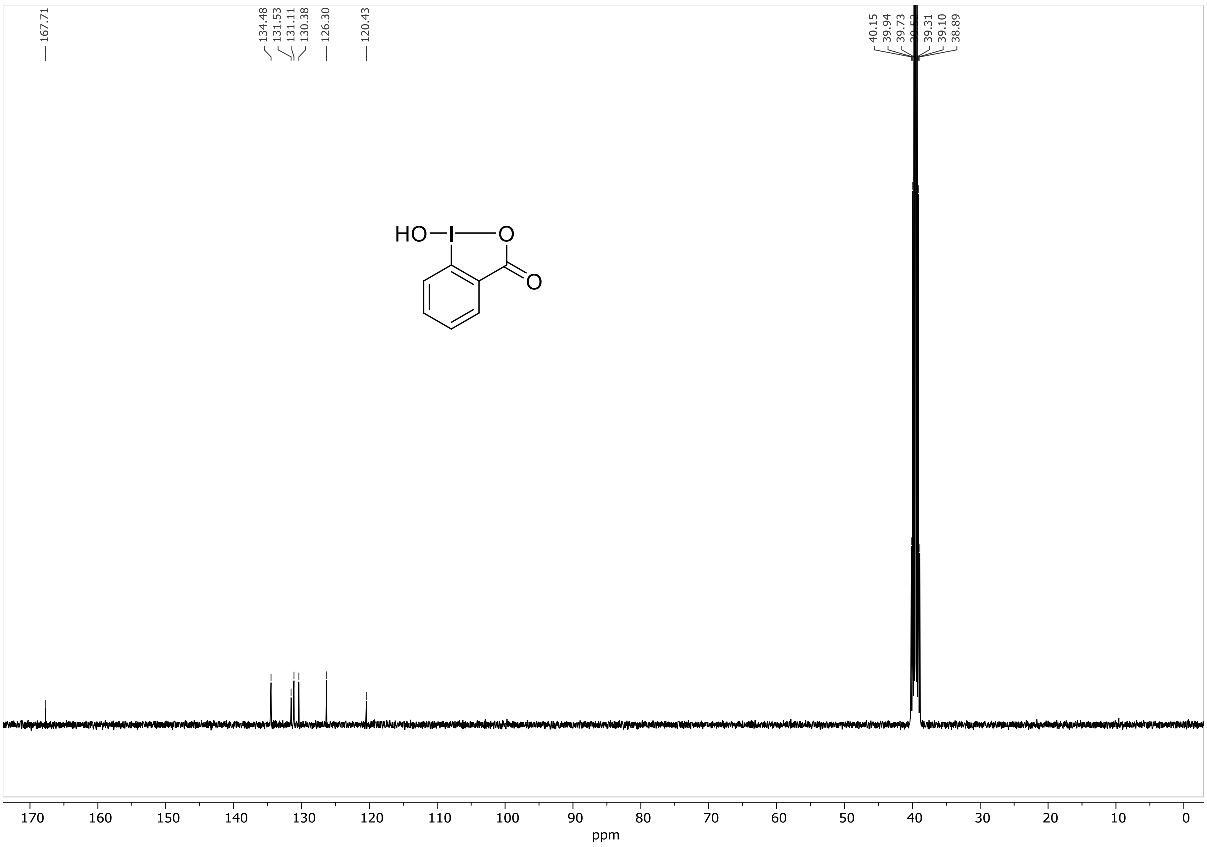

Figure S11. *^{13}C NMR spectrum (DMSO-$_{d6}$, 101 MHz) of 1-hydroxy-1,2-benziodoxol-3-(1H)-one (**1**).*

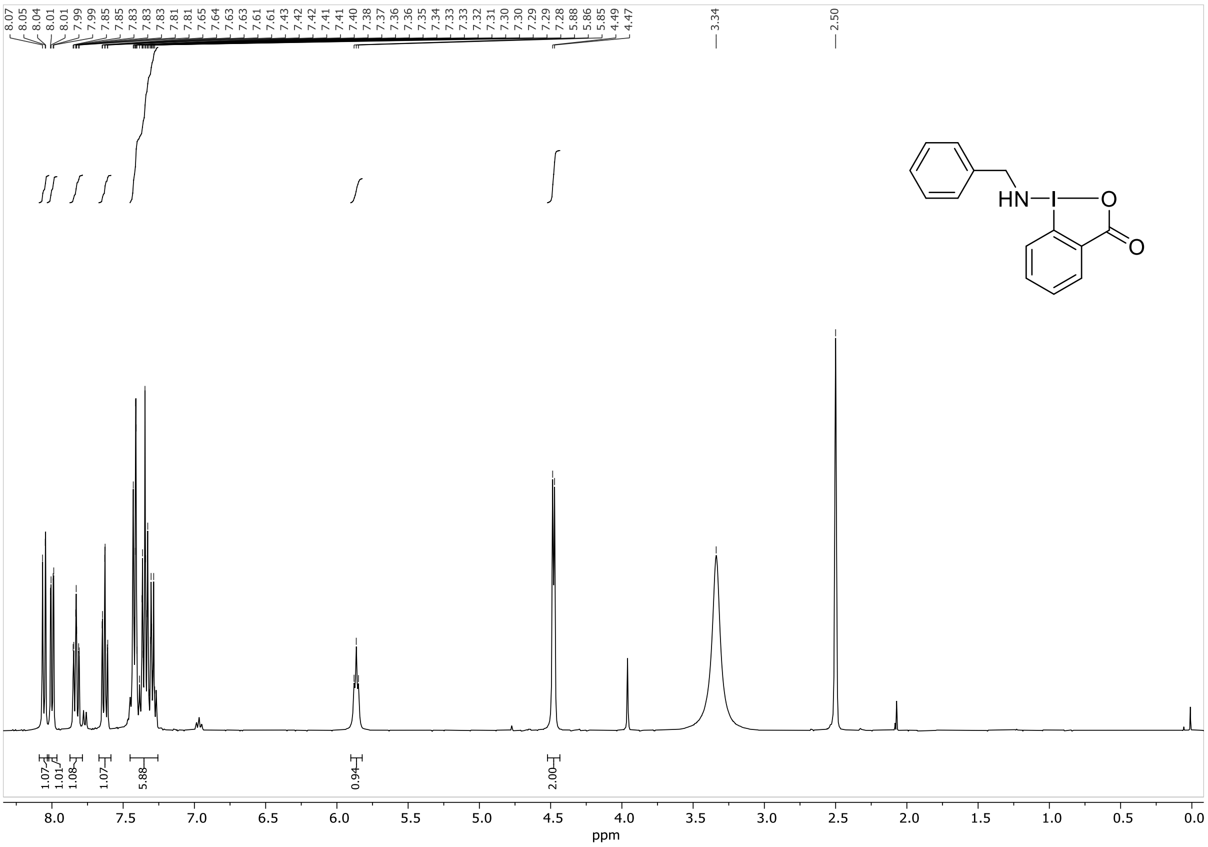

Figure S12. *^{1}H NMR spectrum (DMSO-d6, 400 MHz) of 1-benzylamino-1,2-benziodoxol-3-(1H)-one (**2a**).*

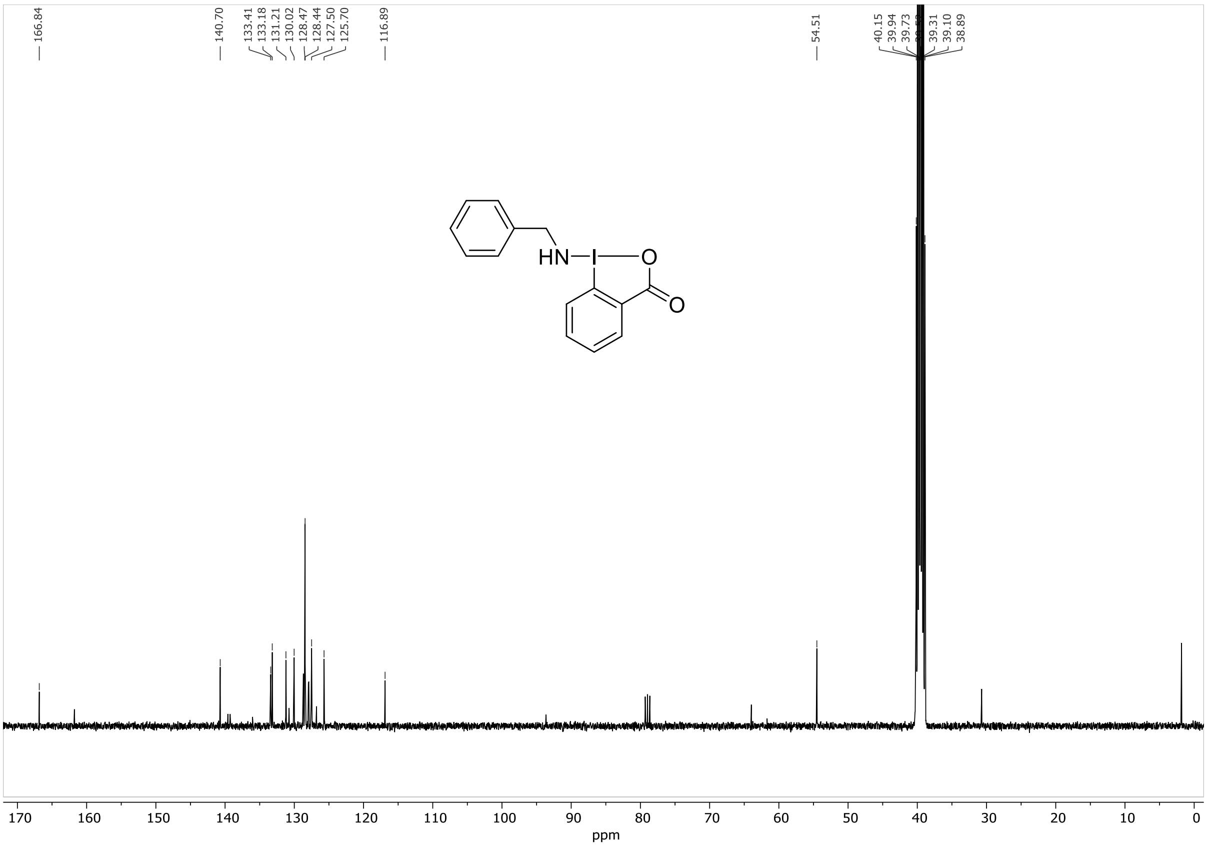

Figure S13. *^{13}C NMR spectrum (DMSO-d6, 101 MHz) of 1-benzylamino-1,2-benziodoxol-3-(1H)-one (**2a**).*

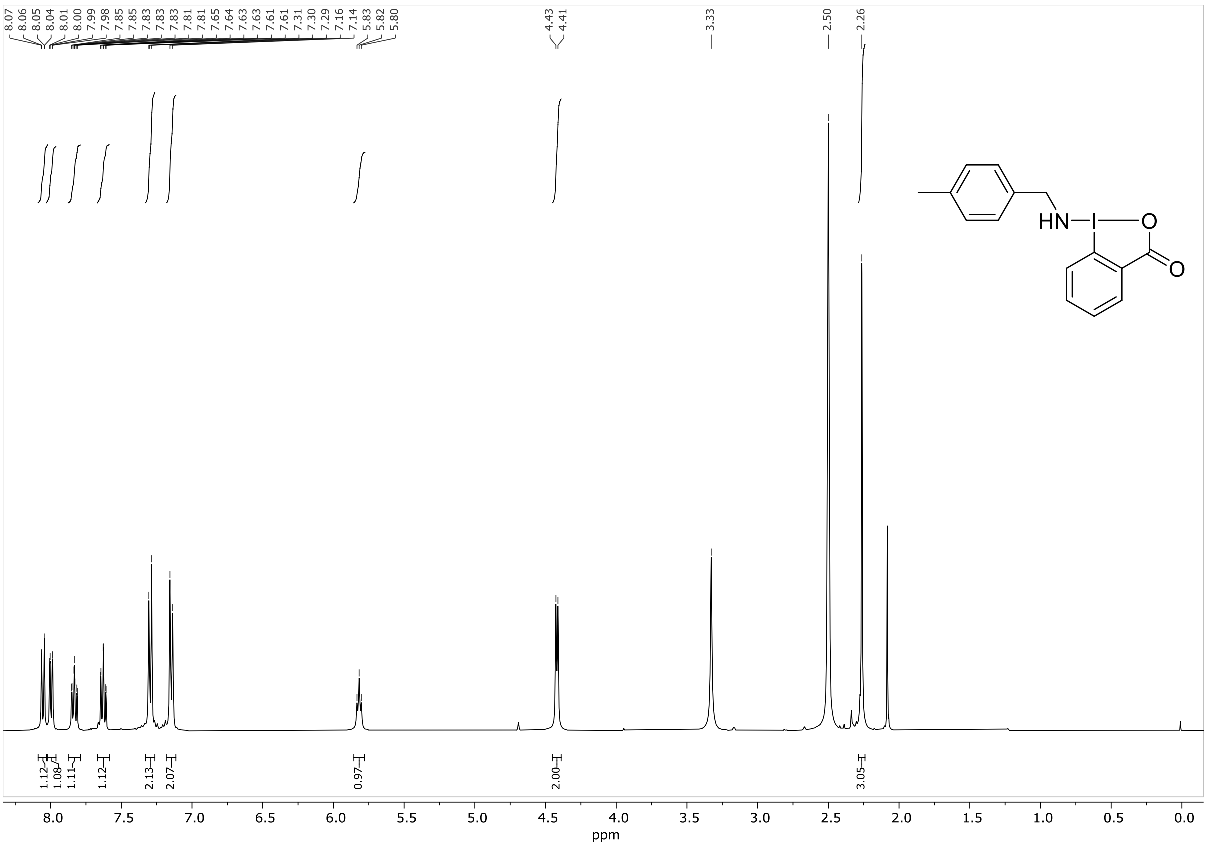

Figure S14.[1]*H NMR spectrum (DMSO-d6, 400 MHz) of 1-((4-methylbenzyl)amino)-1,2-benziodoxol-3-(1H)-one (**2b**).*

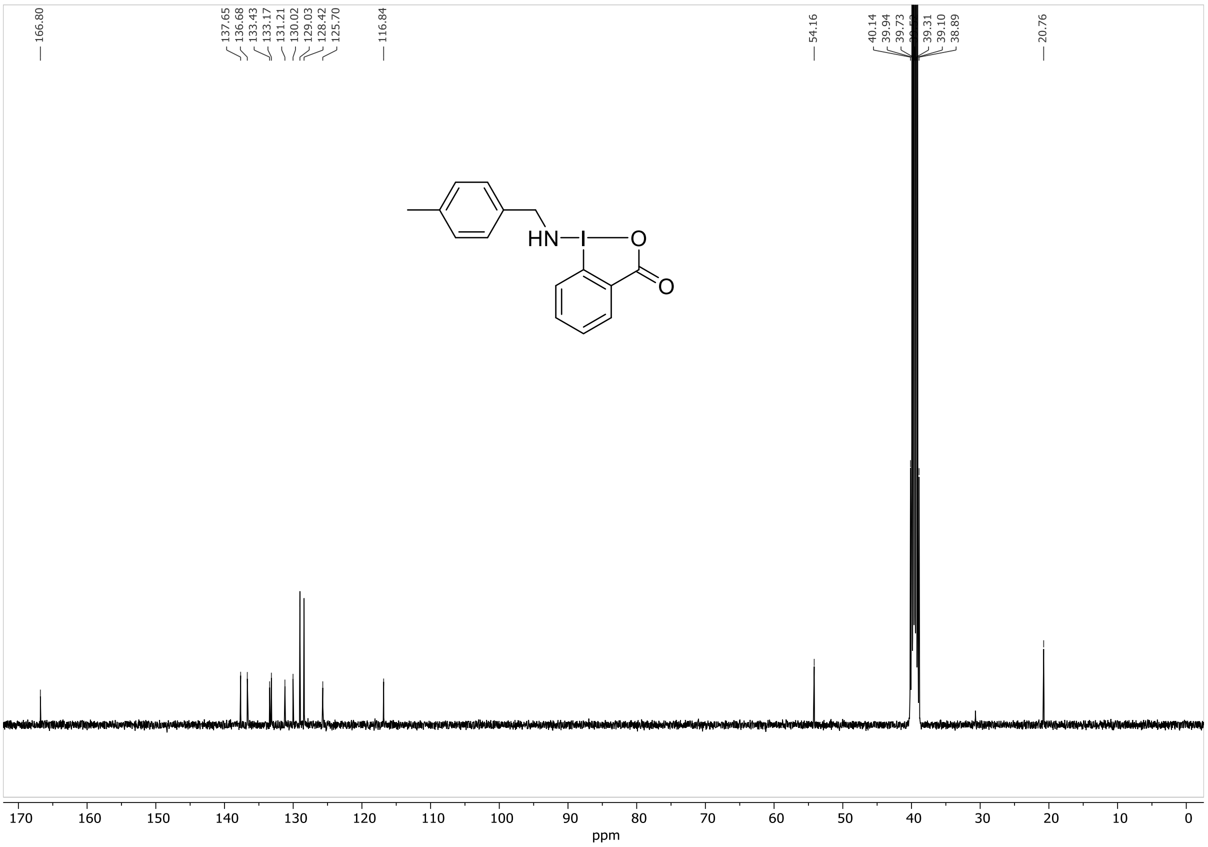

Figure S15.[13]*C NMR spectrum (DMSO-d6, 101 MHz) of 1-((4-methylbenzyl)amino)-1,2-benziodoxol-3-(1H)-one (**2b**).*

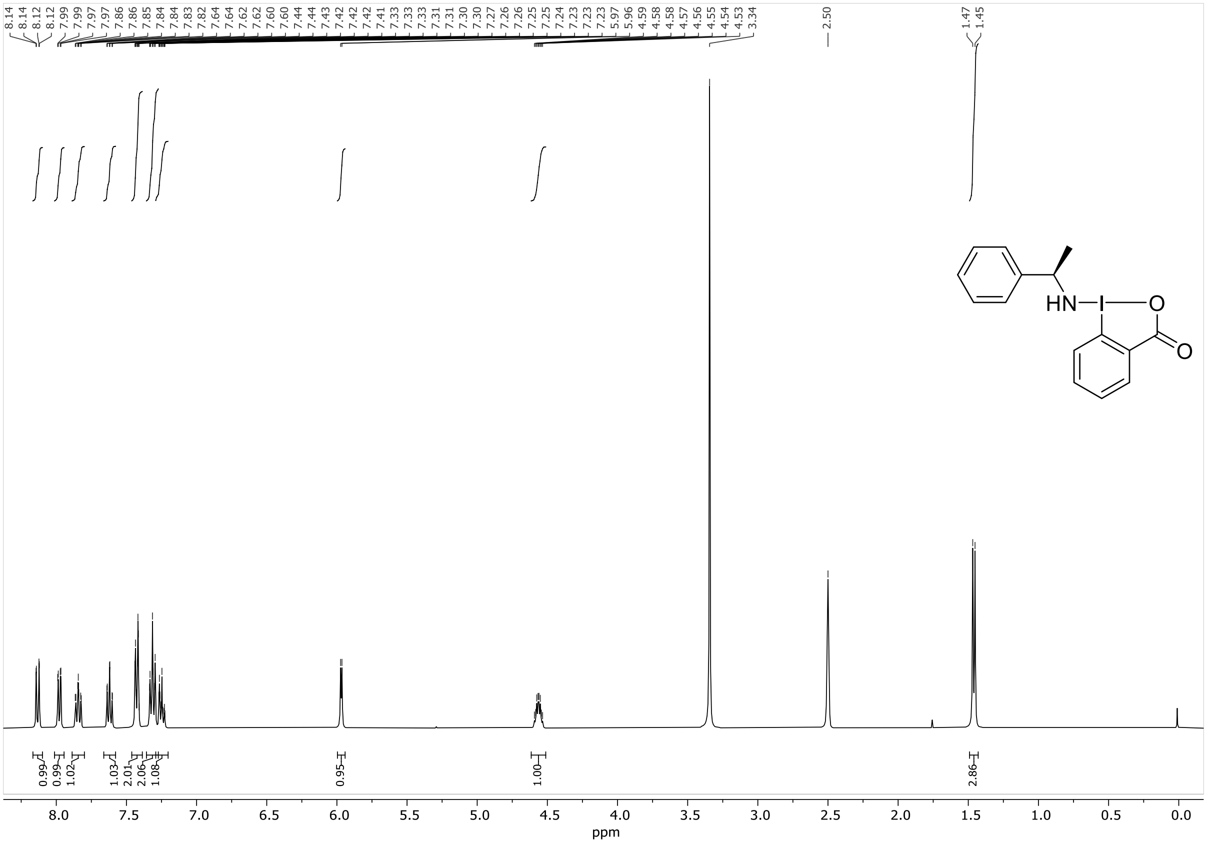

Figure S16. *^{1}H NMR spectrum (DMSO-d6, 400 MHz) of (R)-1-((1-phenylethyl)amino)-1,2-benziodoxol-3-(1H)-one (**2c**).*

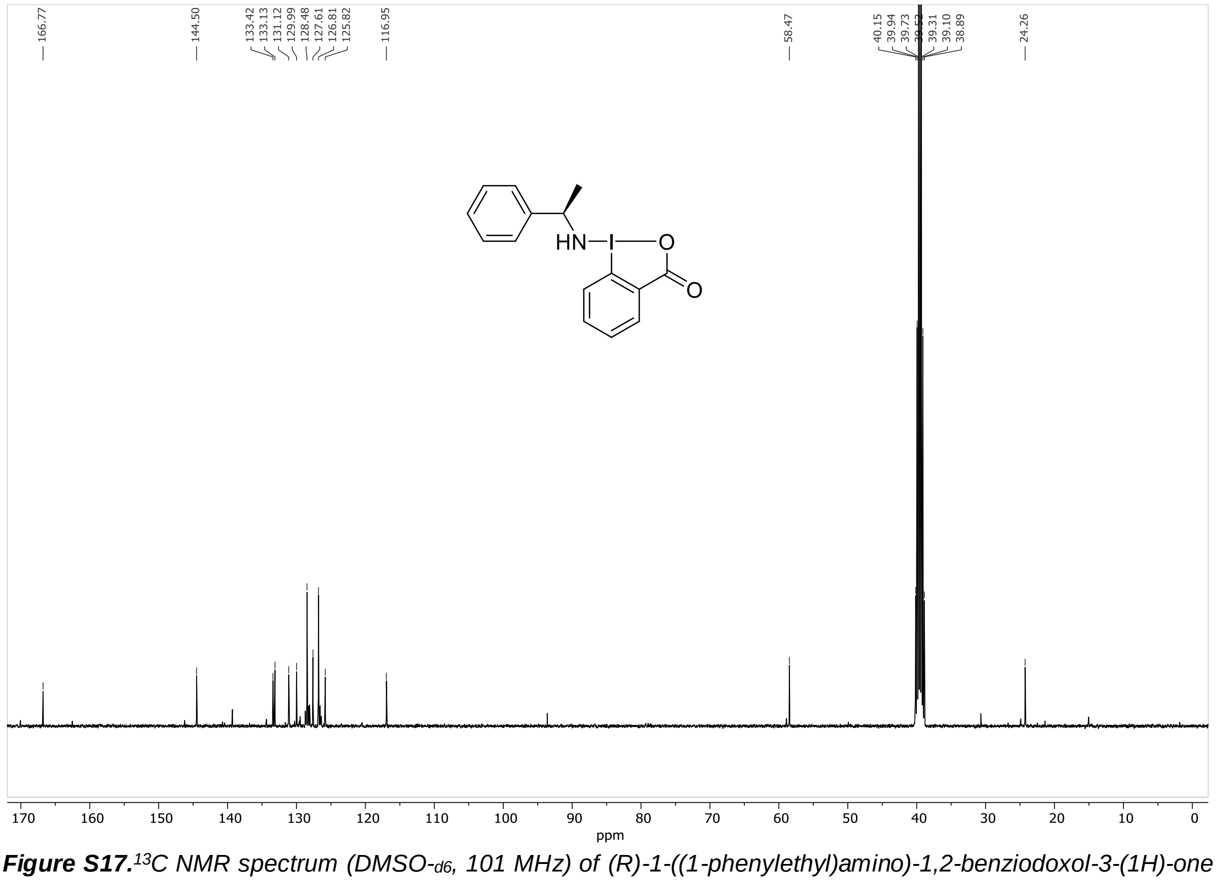

Figure S17. *^{13}C NMR spectrum (DMSO-d6, 101 MHz) of (R)-1-((1-phenylethyl)amino)-1,2-benziodoxol-3-(1H)-one (**2c**).*

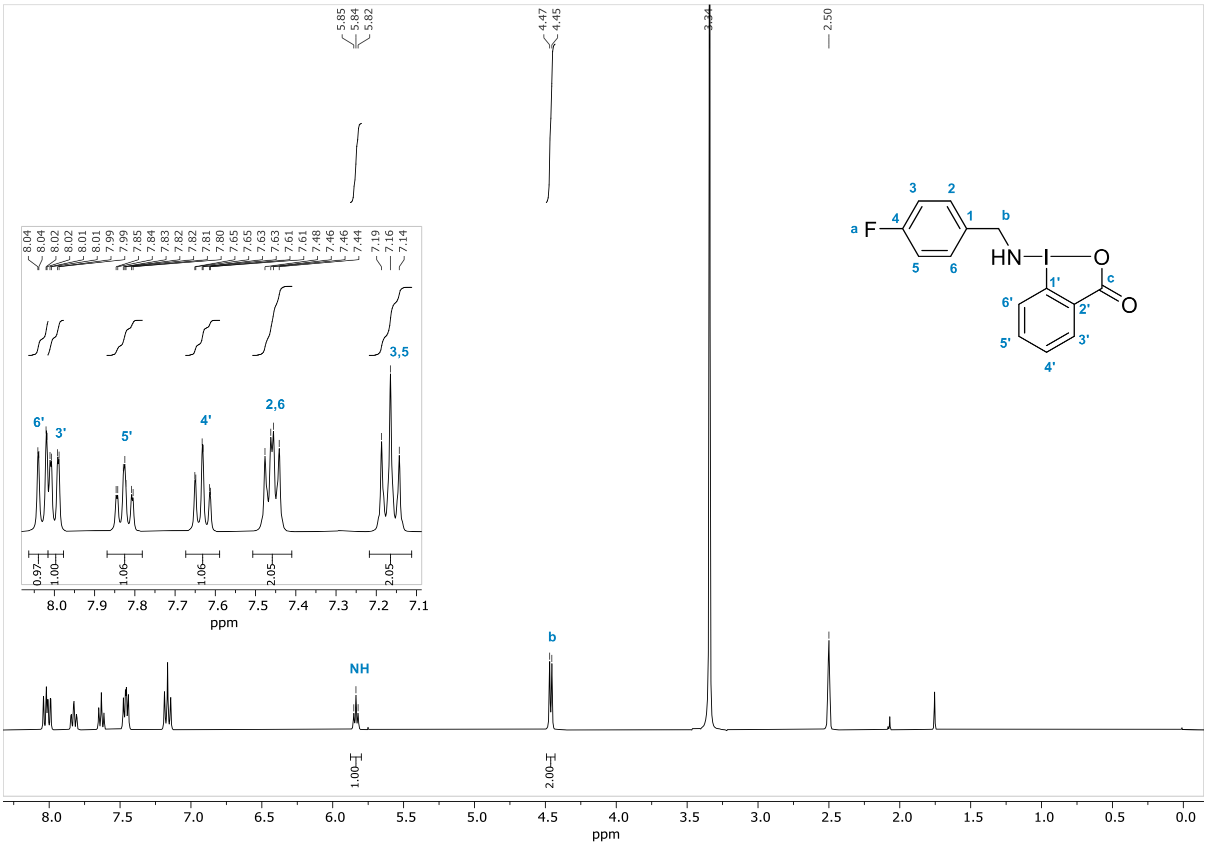

Figure S18.[1]*H NMR spectrum (DMSO-d6, 400 MHz) of 1-((4-fluorobenzyl)amino)-1,2-benziodoxol-3-(1H)-one* ***(2d)***.

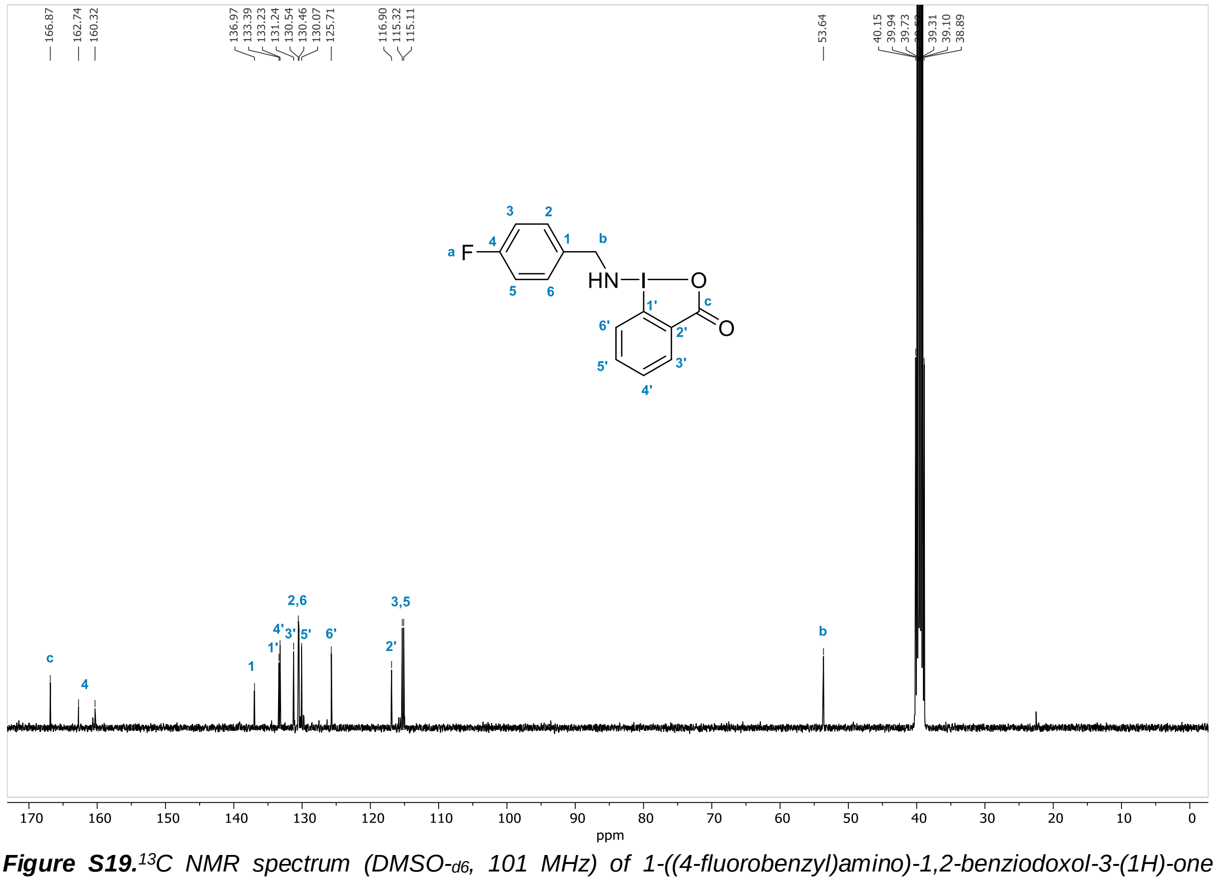

Figure S19.[13]*C NMR spectrum (DMSO-d6, 101 MHz) of 1-((4-fluorobenzyl)amino)-1,2-benziodoxol-3-(1H)-one* ***(2d)***.

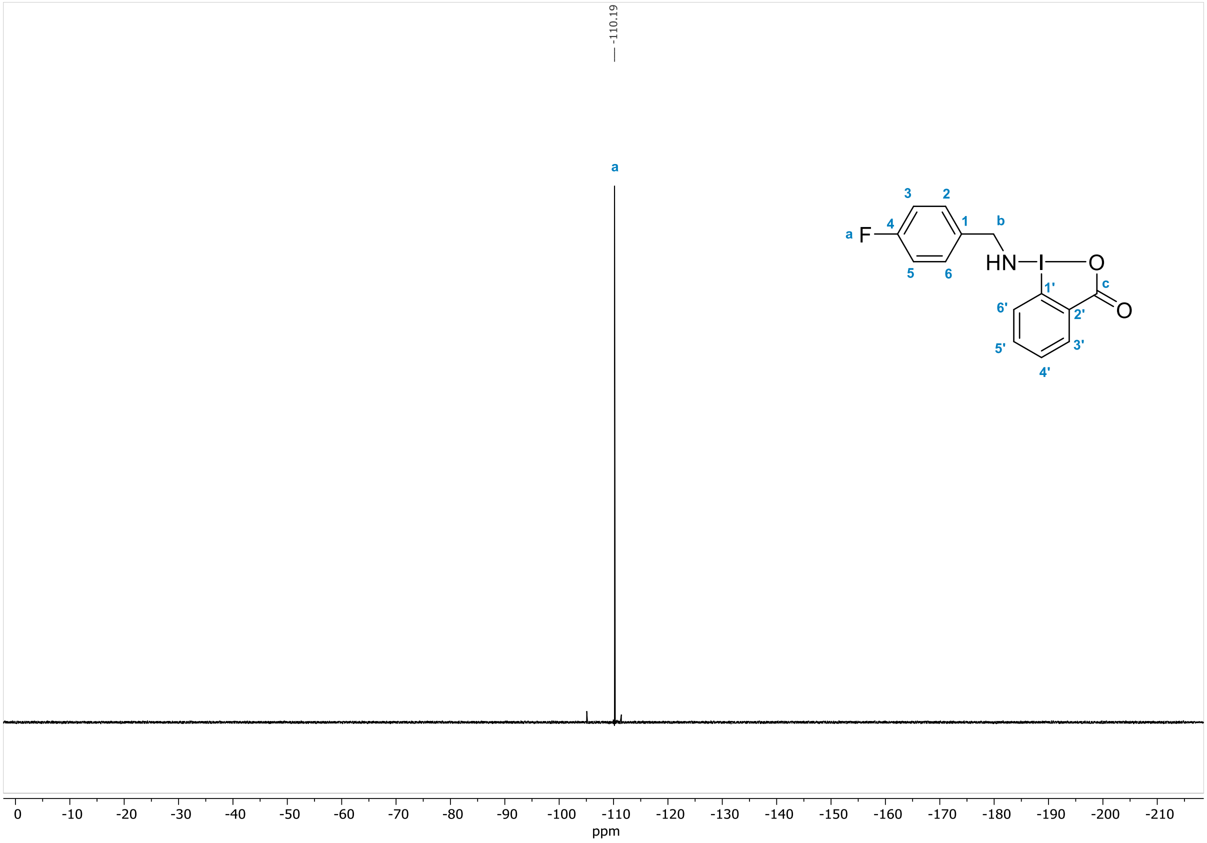

Figure S20. *^{19}F NMR spectrum (DMSO-$_{d6}$, 376 MHz) of 1-((4-fluorobenzyl)amino)-1,2-benziodoxol-3-(1H)-one (**2d**).*

Table S1. *^{1}H, ^{13}C and ^{19}F NMRs assignment of 1-((4-fluorobenzyl)amino)-1,2-benziodoxol-3-(1H)-one (**2d**).*

	^{1}H NMR (ppm)	^{13}C NMR (ppm)	^{19}F NMR (ppm)
1		136.99 (d, *J* = 3.2 Hz)	
2, 6	7.46 (dd, *J* = 8.5, 5.8 Hz)	130.50 (d, *J* = 8.1 Hz)	
3, 5	7.16 (t, *J* = 8.9 Hz)	115.22 (d, *J* = 21.3 Hz)	
4		161.53 (d, *J* = 243.3 Hz)	
1’		133.39	
2’		116.90	
3’	8.00 (dd, *J* = 7.5, 1.5 Hz)	131.24	
4’	7.83 (ddd, *J* = 8.2, 7.1, 1.6 Hz)	133.23	
5’	7.63 (td, *J* = 7.3, 1.0 Hz)	130.07	
6’	8.03 (dd, *J* = 8.2, 1.0 Hz, 1H)	125.71	
a			-100.19
b	4.46 (d, *J* = 6.1 Hz)	53.64	
c		166.87	
NH	5.84 (t, *J* = 6.1 Hz)		

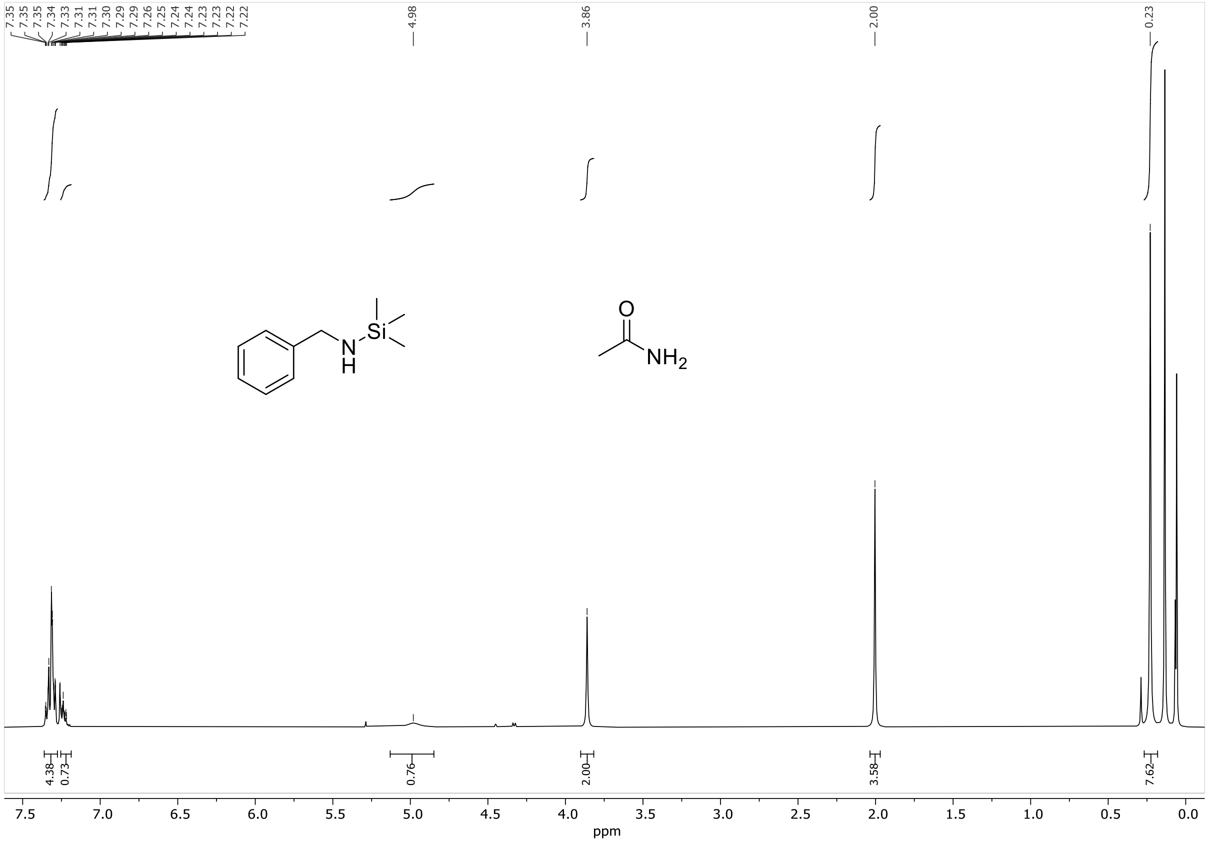

Figure S21. *^{1}H NMR spectrum (CDCl$_3$, 400 MHz) of 1,1,1-trimethyl-N-benzyl-silanamine (**S1**).*

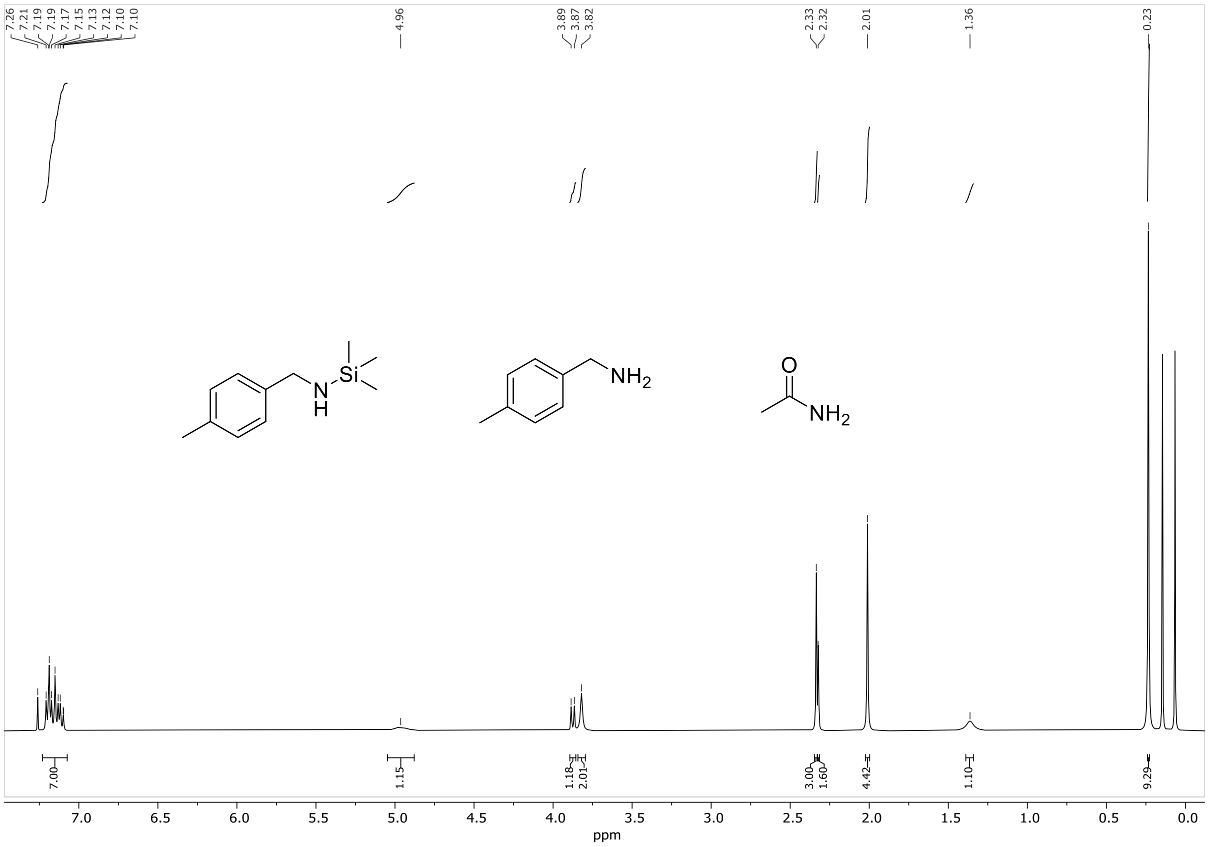

Figure S22. *^{1}H NMR spectrum (CDCl$_3$, 400 MHz) of 1,1,1-trimethyl-N-(4-methylbenzyl)silanamine (**S2**).*

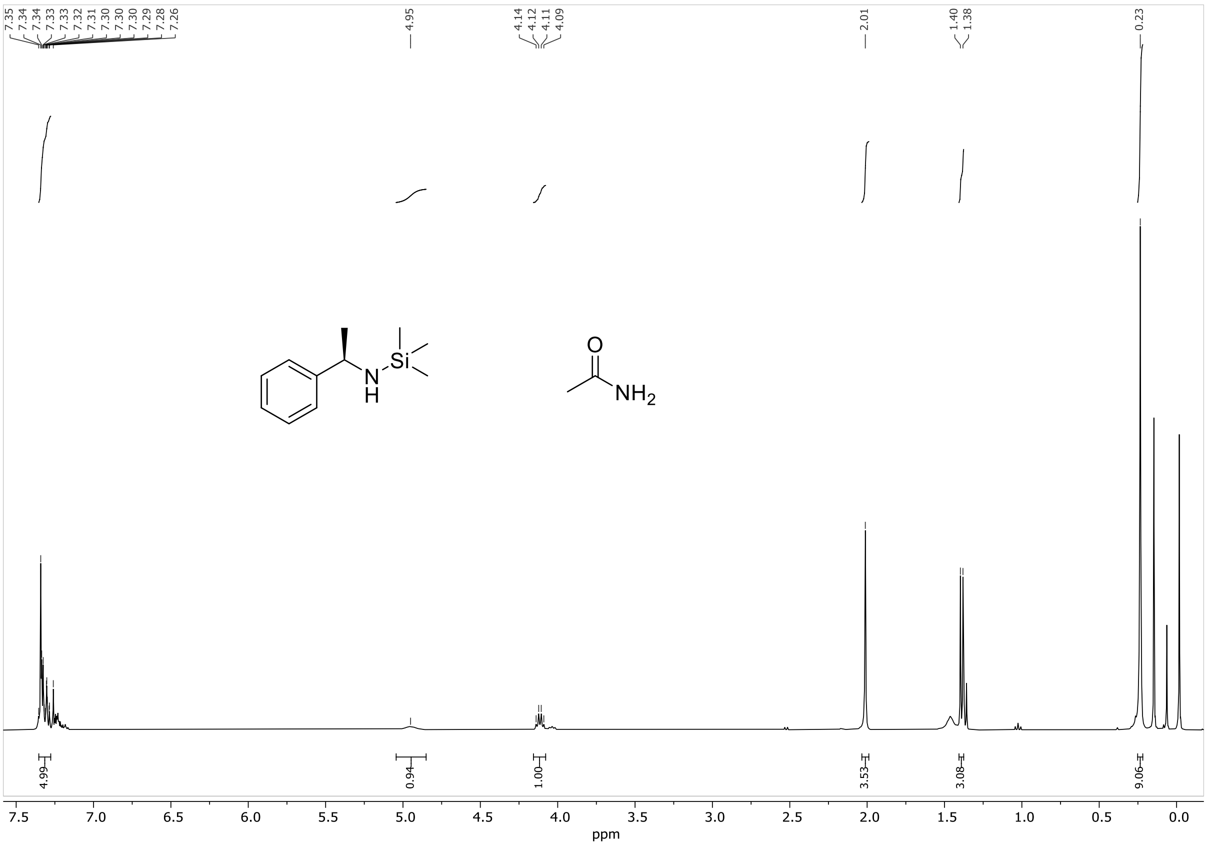

Figure S23.[1]*H NMR spectrum (CDCl*$_3$*, 400 MHz) of (R)-1,1,1-trimethyl-N-(1-phenylethyl)silanamine (**S3**).*

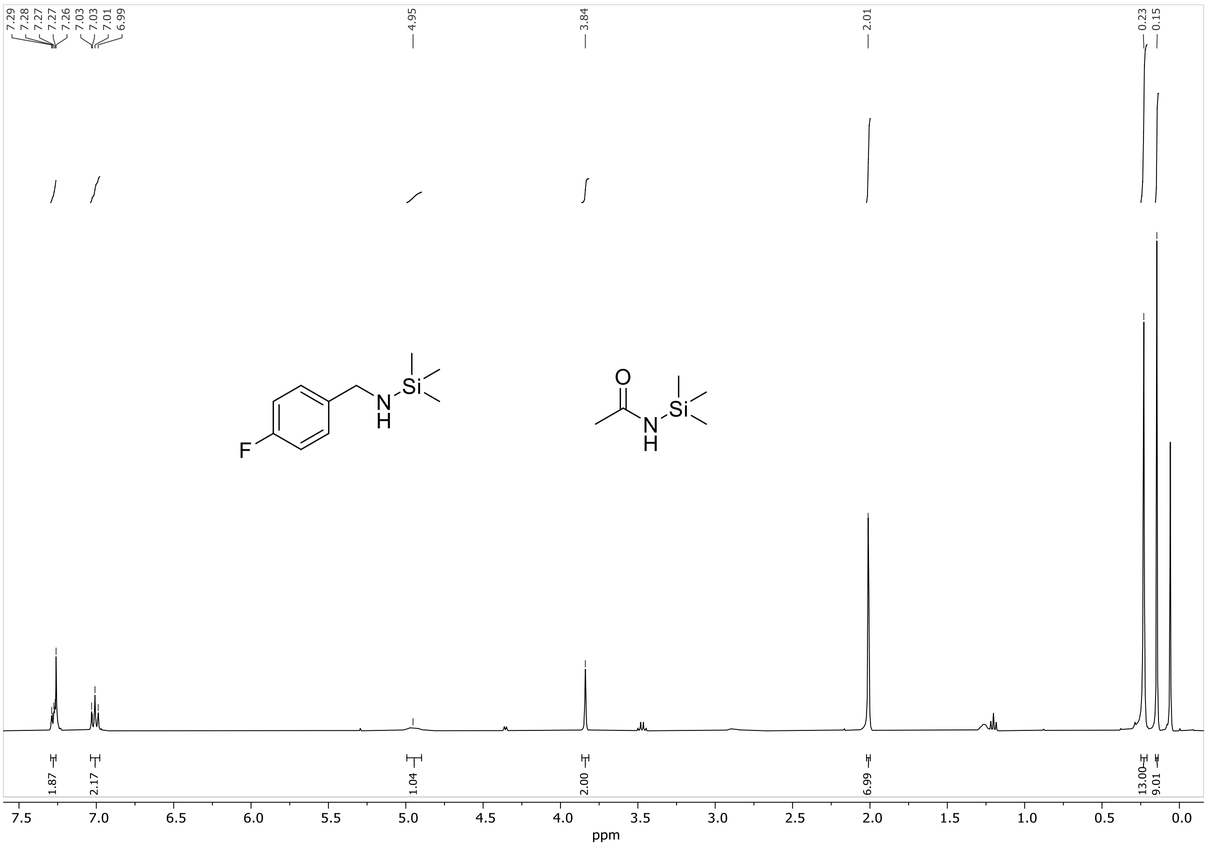

Figure S24.[1]*H NMR spectrum (CDCl*$_3$*, 400 MHz) of 1,1,1-trimethyl-N-(4-fluorobenzyl)silanamine (**S4**).*

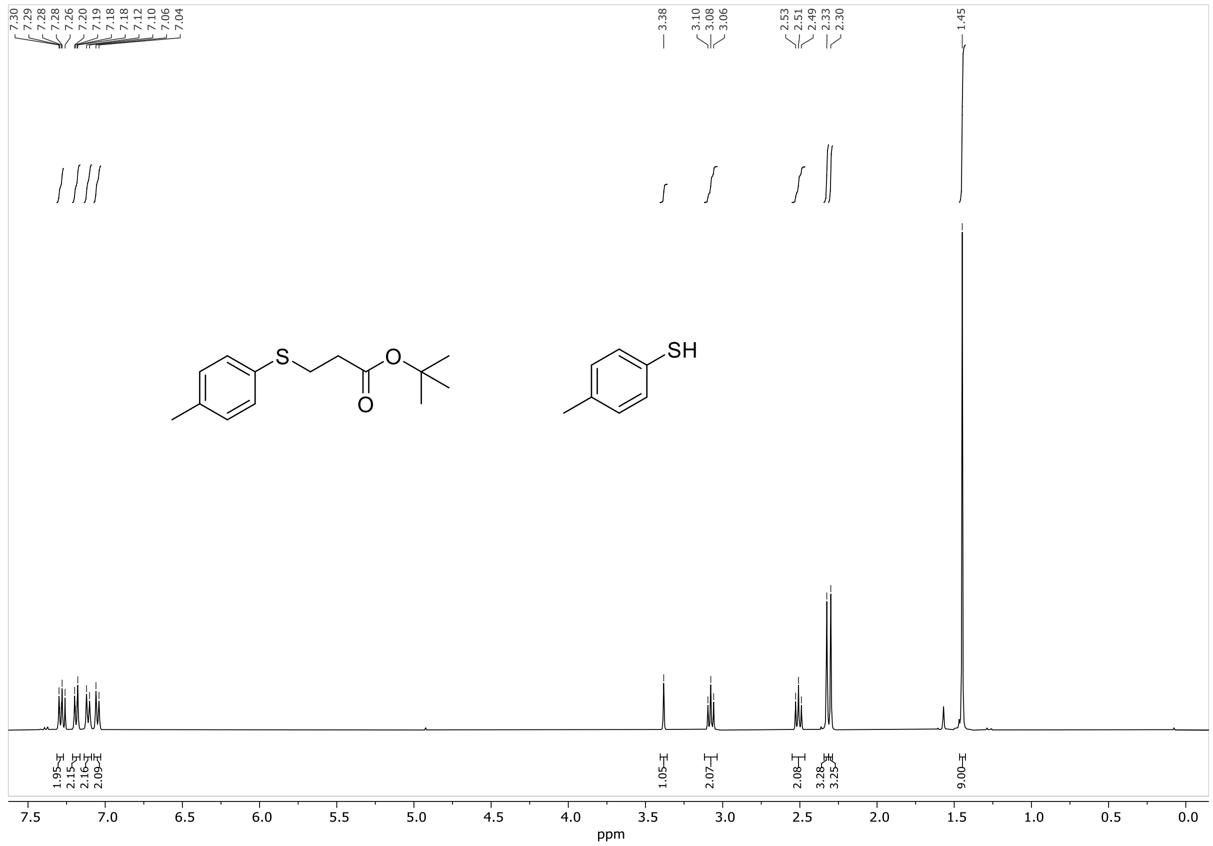

Figure S25. *^{1}H NMR spectrum (CDCl$_3$, 400 MHz) of tert-butyl 3-(p-tolylthio)propanoate (**3a**).*

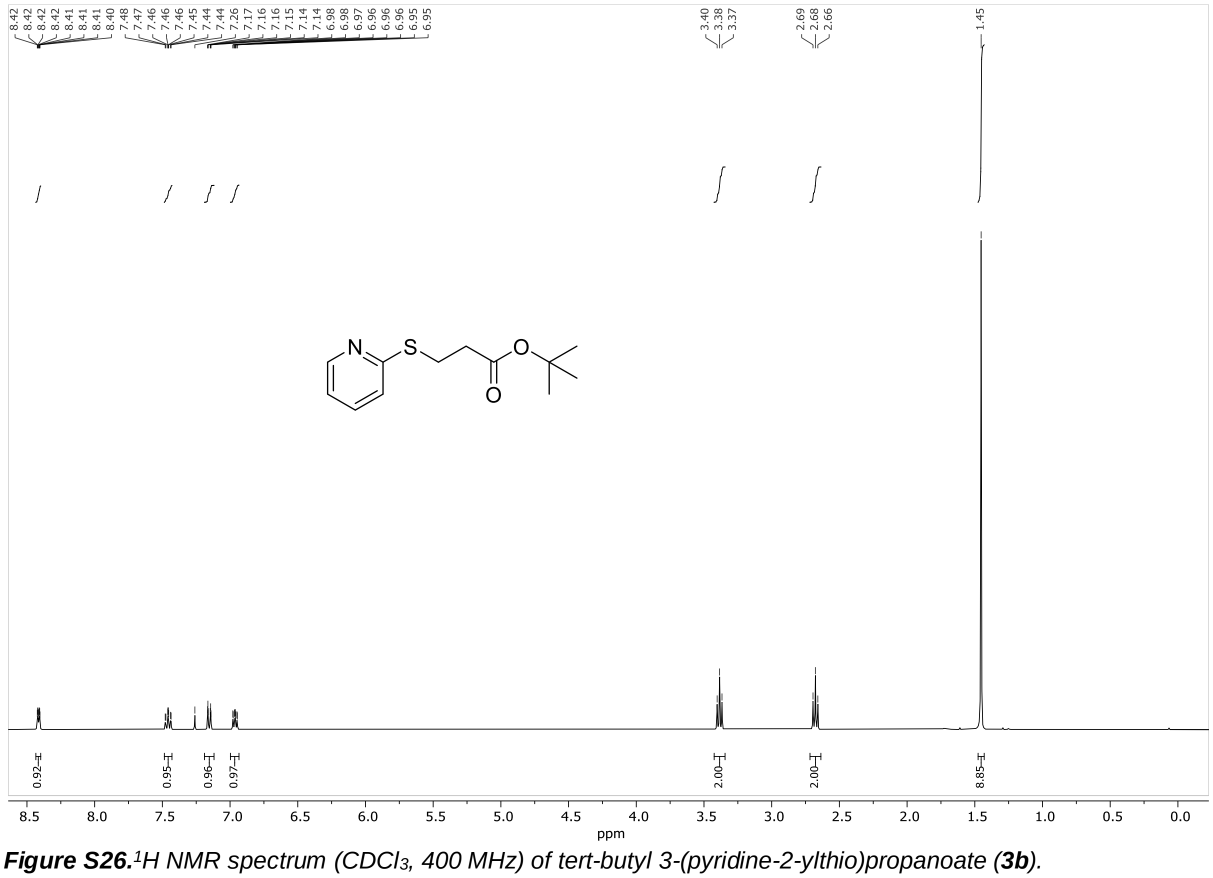

Figure S26. *^{1}H NMR spectrum (CDCl$_3$, 400 MHz) of tert-butyl 3-(pyridine-2-ylthio)propanoate (**3b**).*

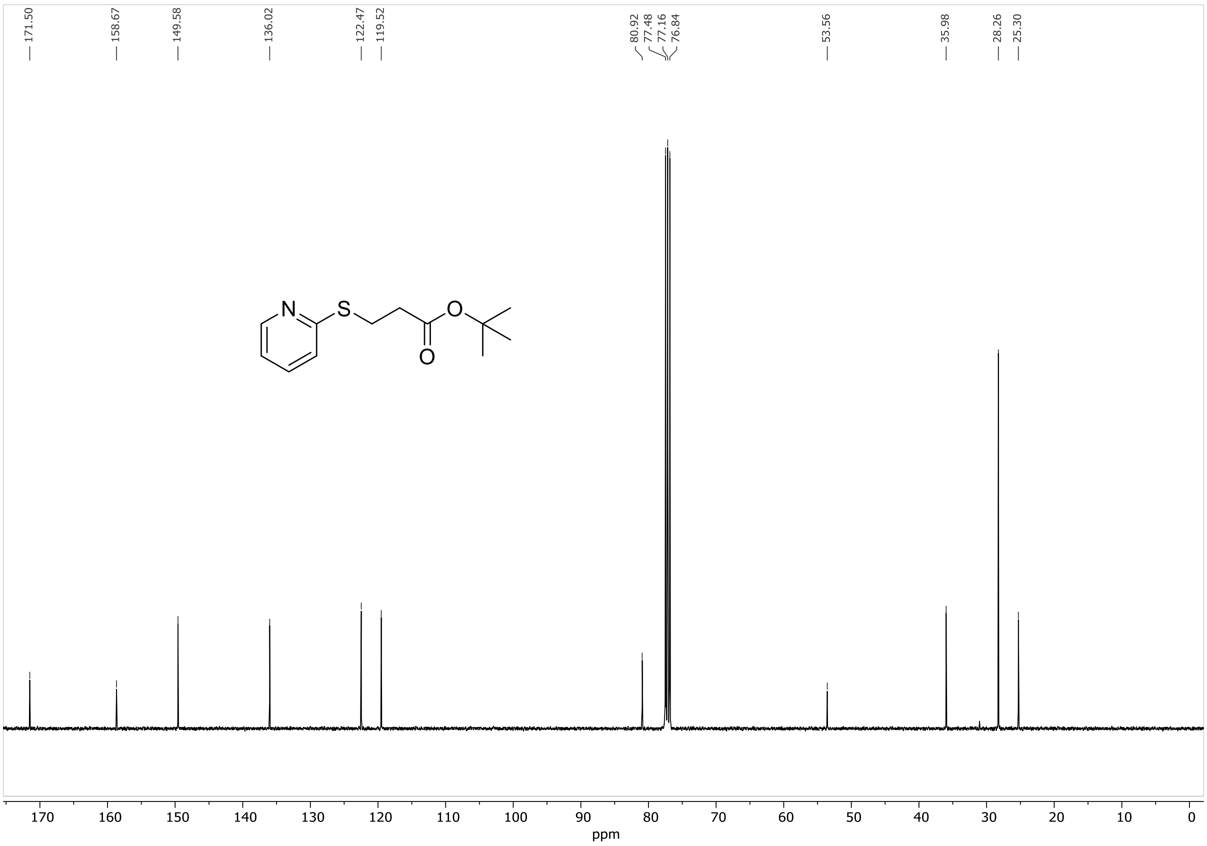

Figure S27. *^{13}C NMR spectrum (CDCl$_3$, 101 MHz) of tert-butyl 3-(pyridine-2-ylthio)propanoate (**3b**).*

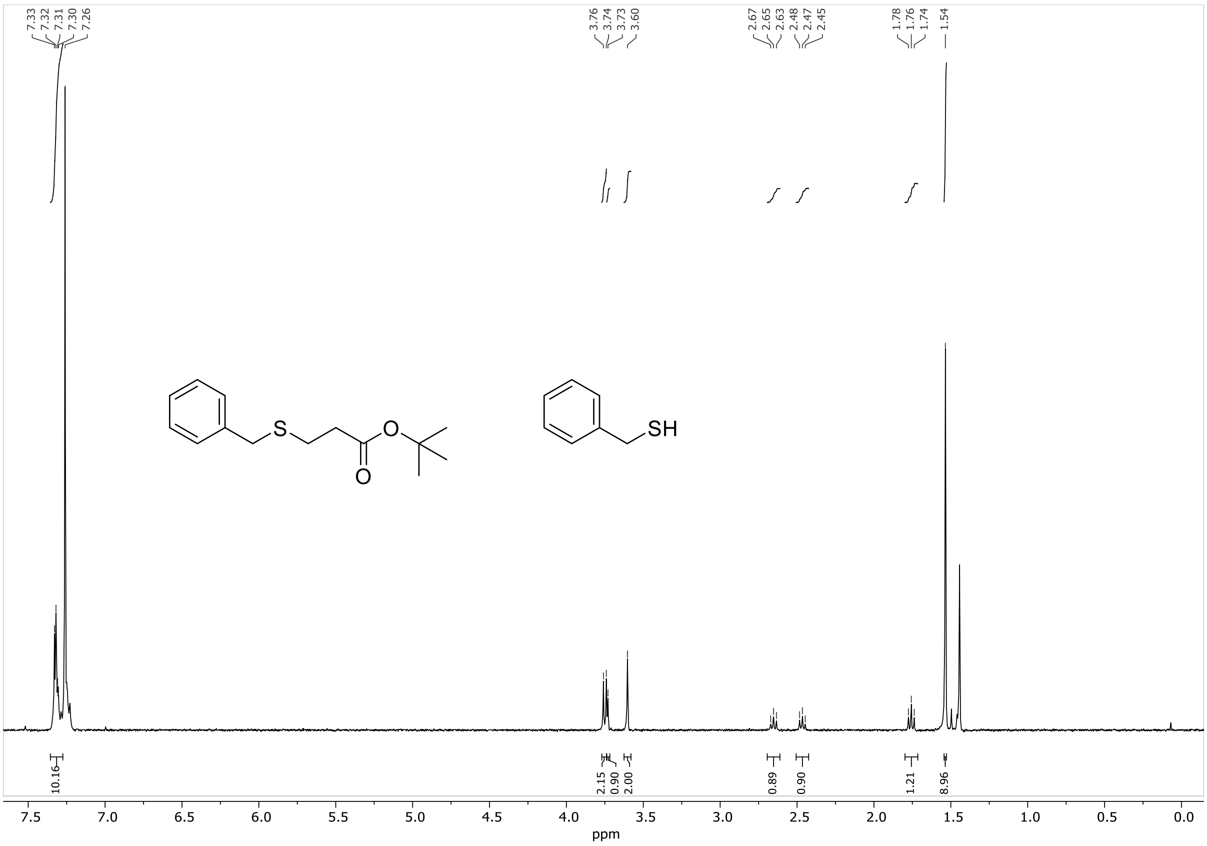

Figure S28. *^{1}H NMR spectrum (CDCl$_3$, 400 MHz) of tert-butyl 3-(benzylthio)propanoate (**3c**).*

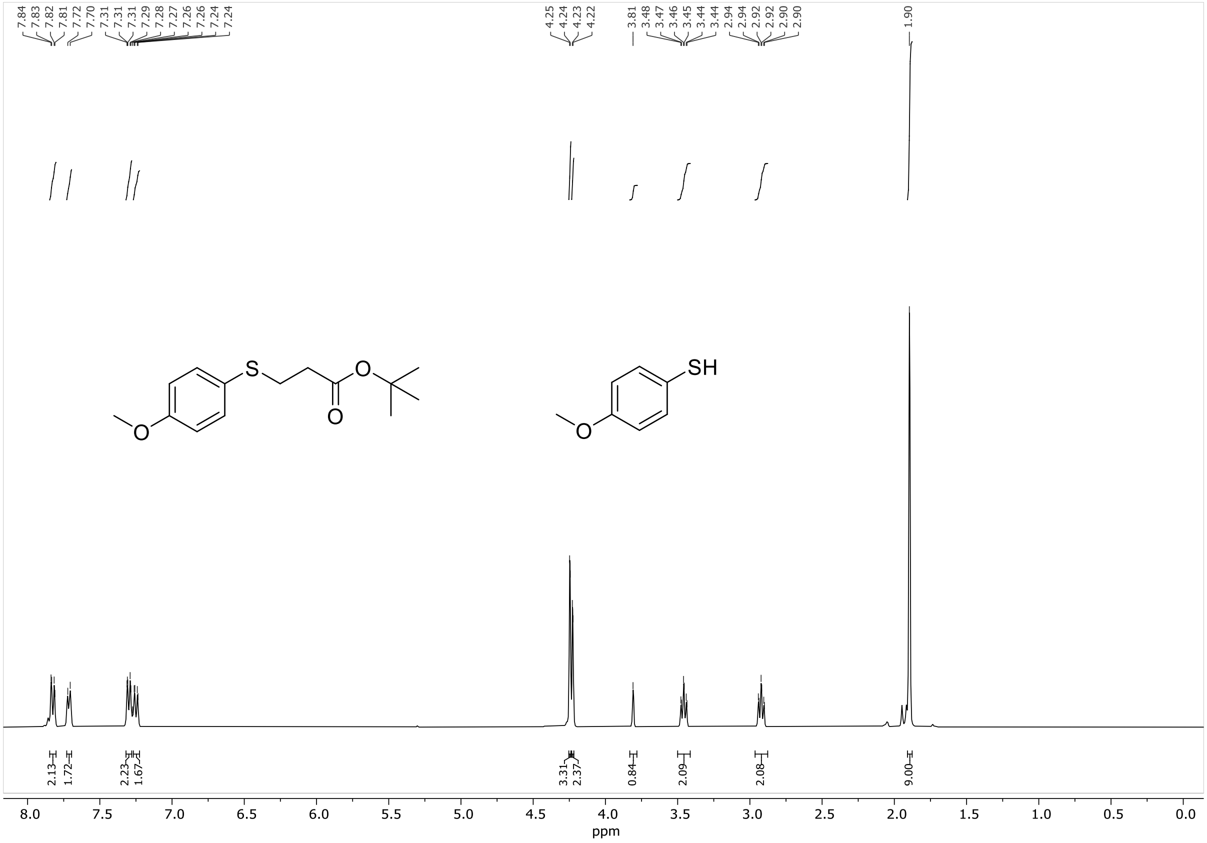

Figure S29.^{1}*H NMR spectrum (CDCl$_3$, 400 MHz) of tert-butyl 3-(4-methoxyphenylthio)propanoate (**3d**).*

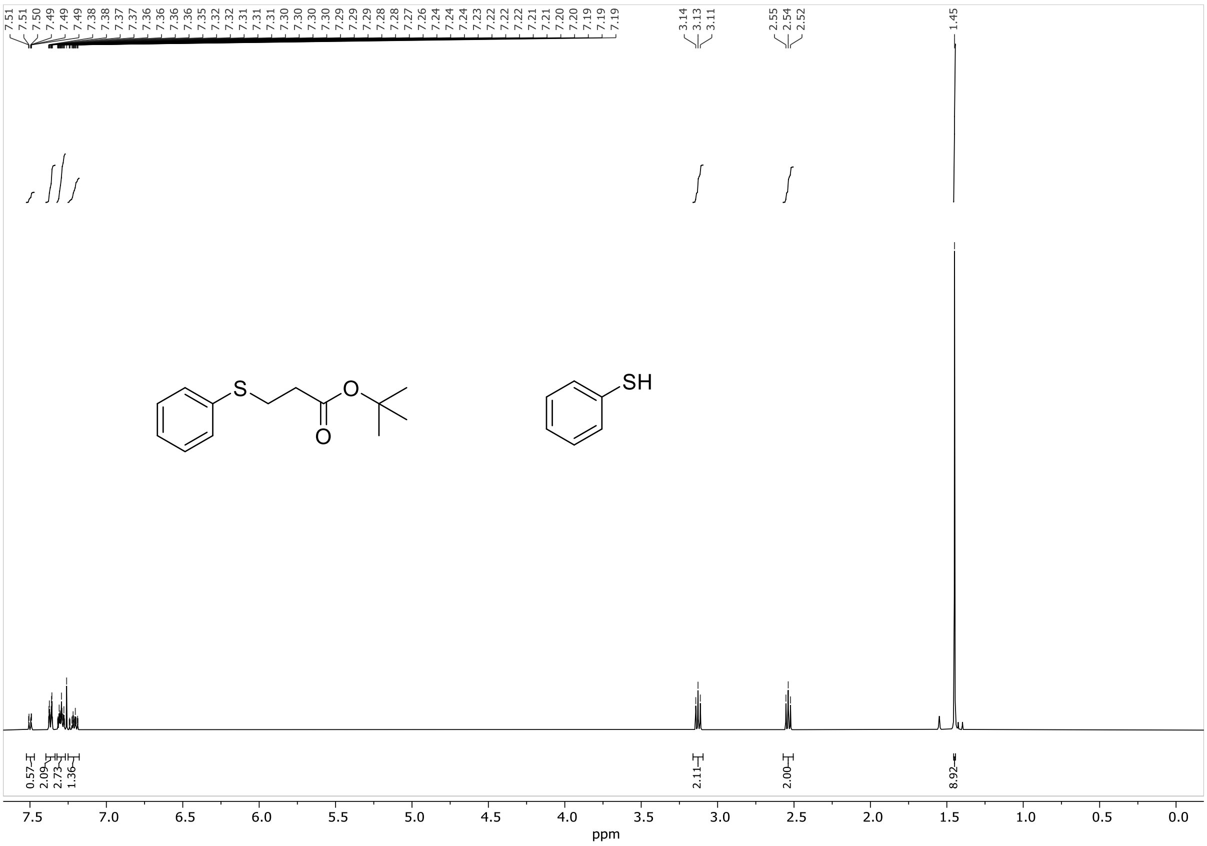

Figure S30.^{1}*H NMR spectrum (CDCl$_3$, 400 MHz) of tert-butyl 3-(phenylsulfanyl)propanoate (**3e**).*

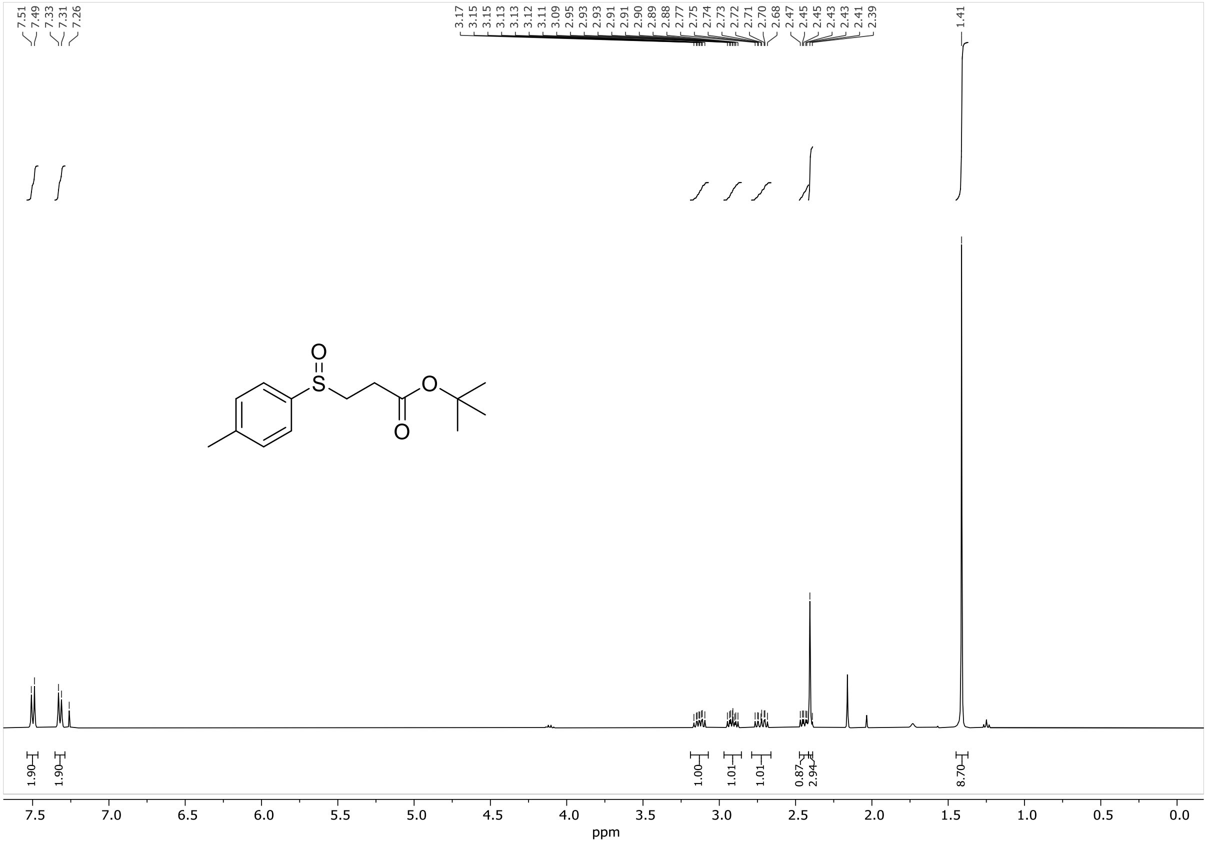

Figure S31.[1]*H NMR spectrum (CDCl$_3$, 400 MHz) of tert-butyl 3-(p-tolylsulfinyl)propanoate (**4a**).*

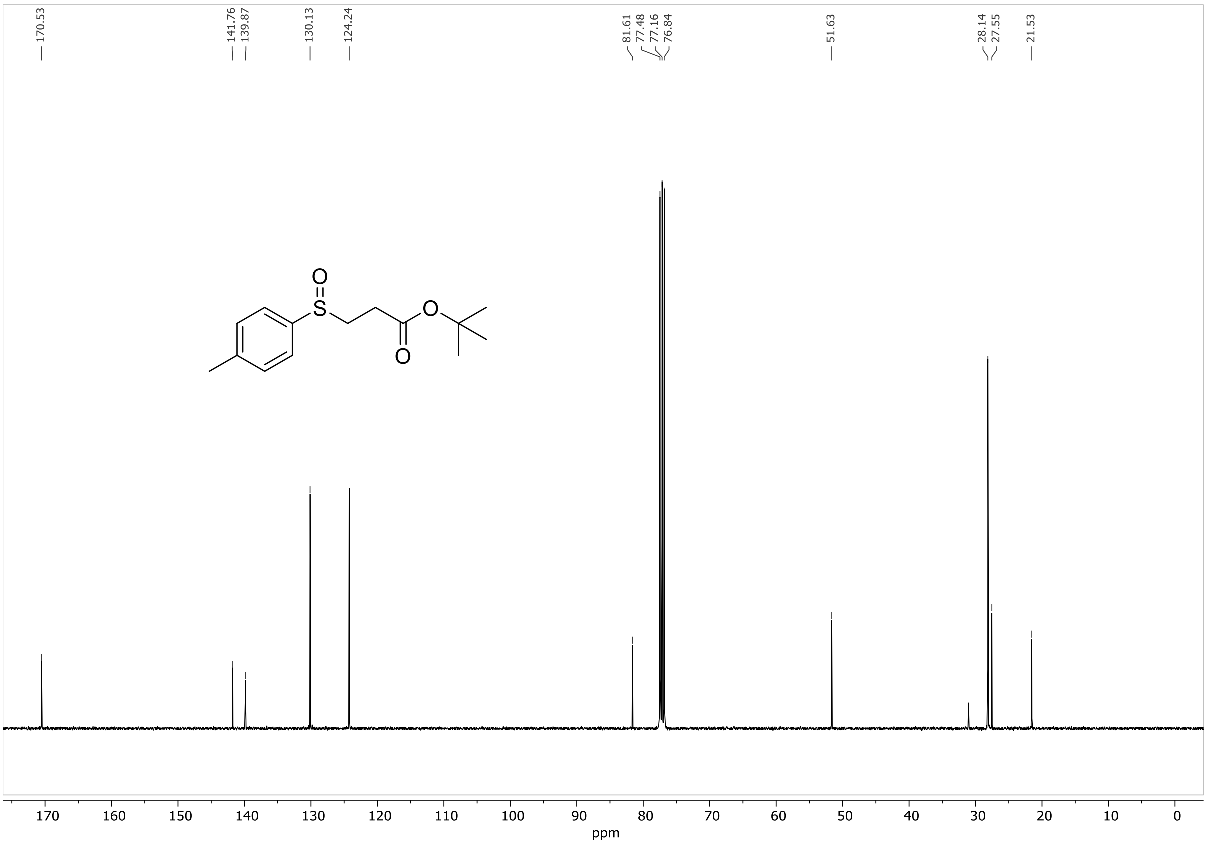

Figure S32.[13]*C NMR spectrum (CDCl$_3$, 101 MHz) of tert-butyl 3-(p-tolylsulfinyl)propanoate (**4a**).*

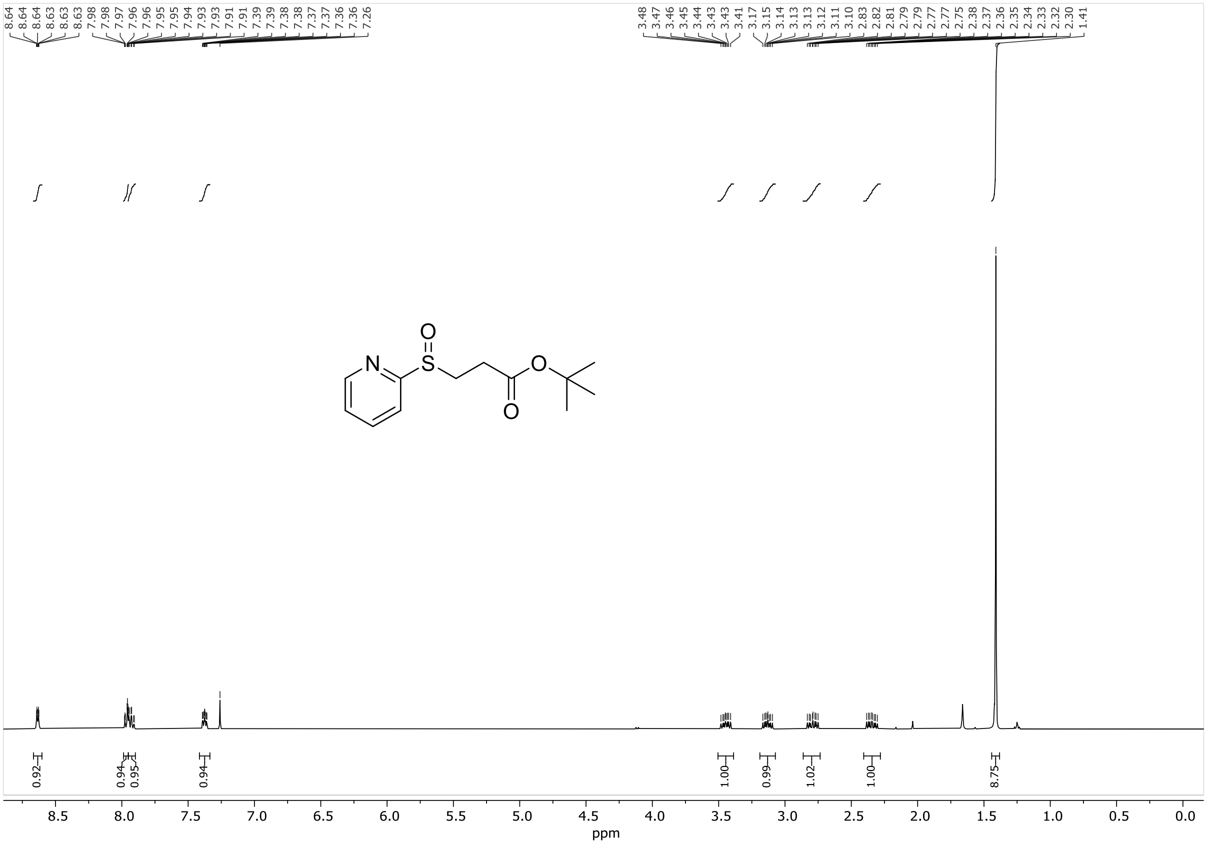

Figure S33.^{1}*H NMR spectrum (CDCl*$_3$*, 400 MHz) of tert-butyl 3-(pyridine-2-ylsulfinyl)propanoate (**4b**).*

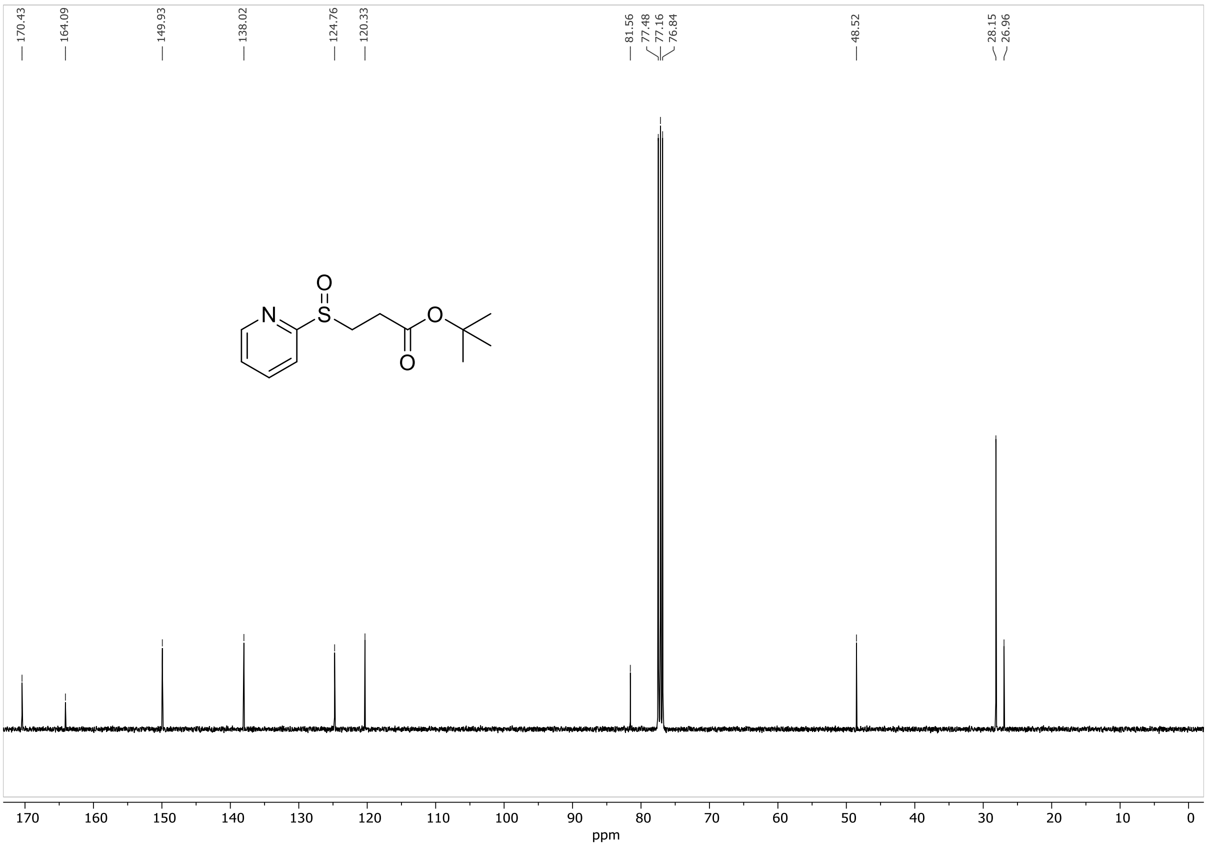

Figure S34.^{13}*C NMR spectrum (CDCl*$_3$*, 101 MHz) of tert-butyl 3-(pyridine-2-ylsulfinyl)propanoate (**4b**).*

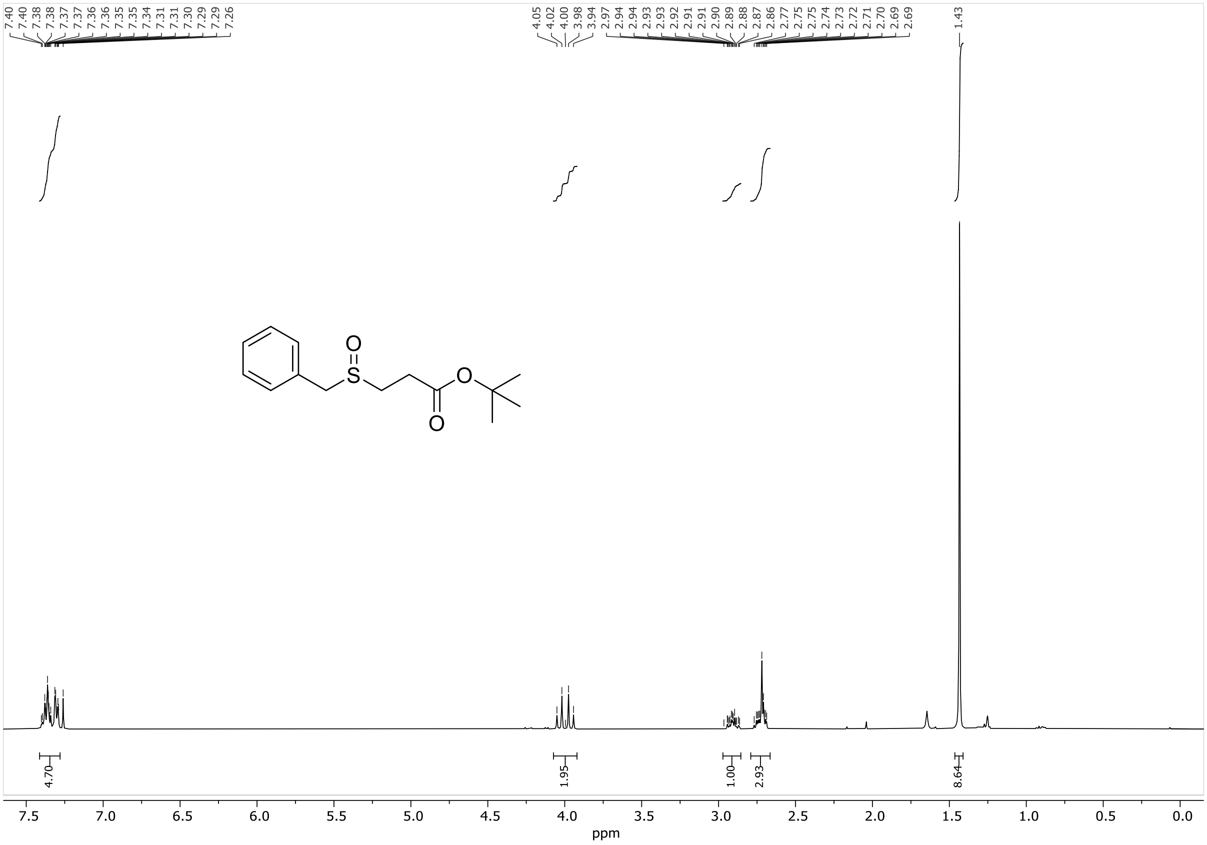

Figure S35. *^{1}H NMR spectrum (CDCl$_3$, 400 MHz) of tert-butyl 3-(benzylsulfinyl)propanoate (**4c**).*

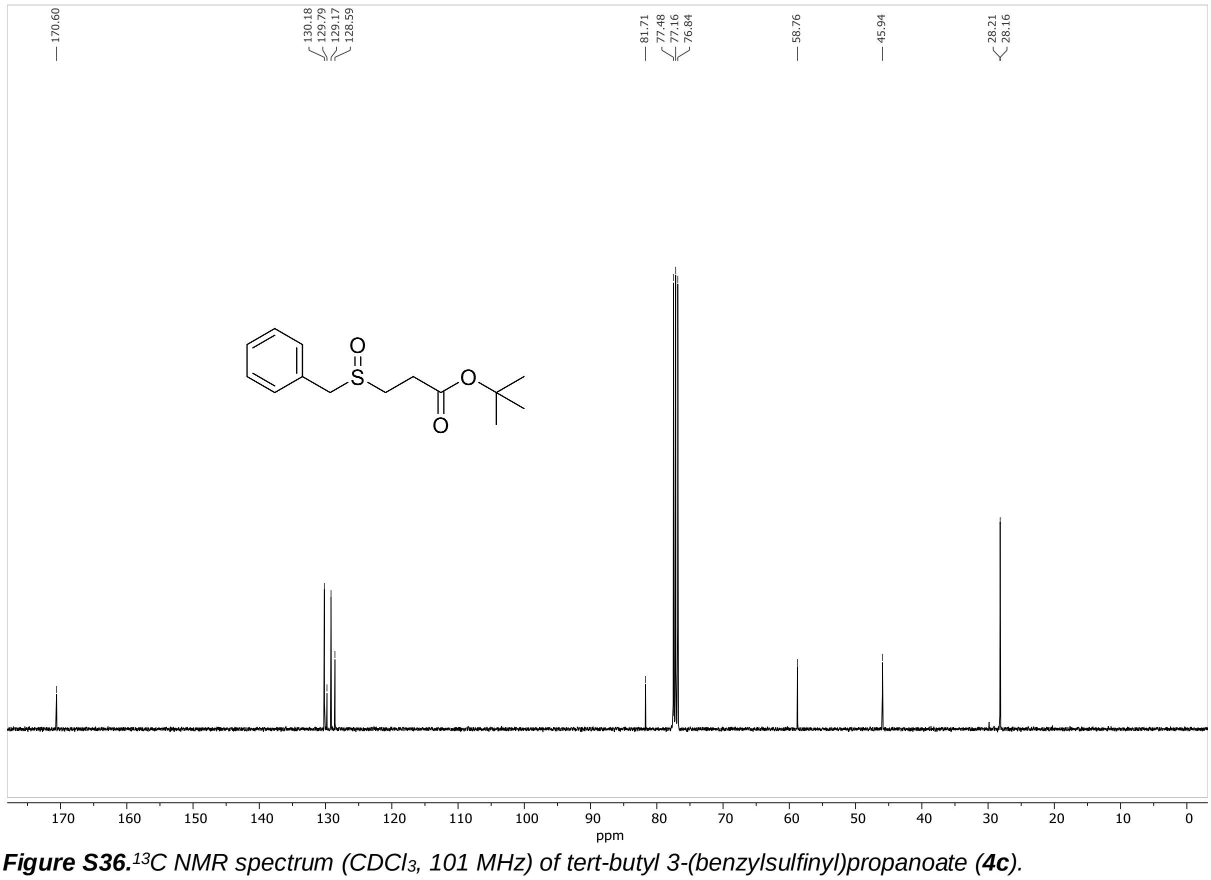

Figure S36. *^{13}C NMR spectrum (CDCl$_3$, 101 MHz) of tert-butyl 3-(benzylsulfinyl)propanoate (**4c**).*

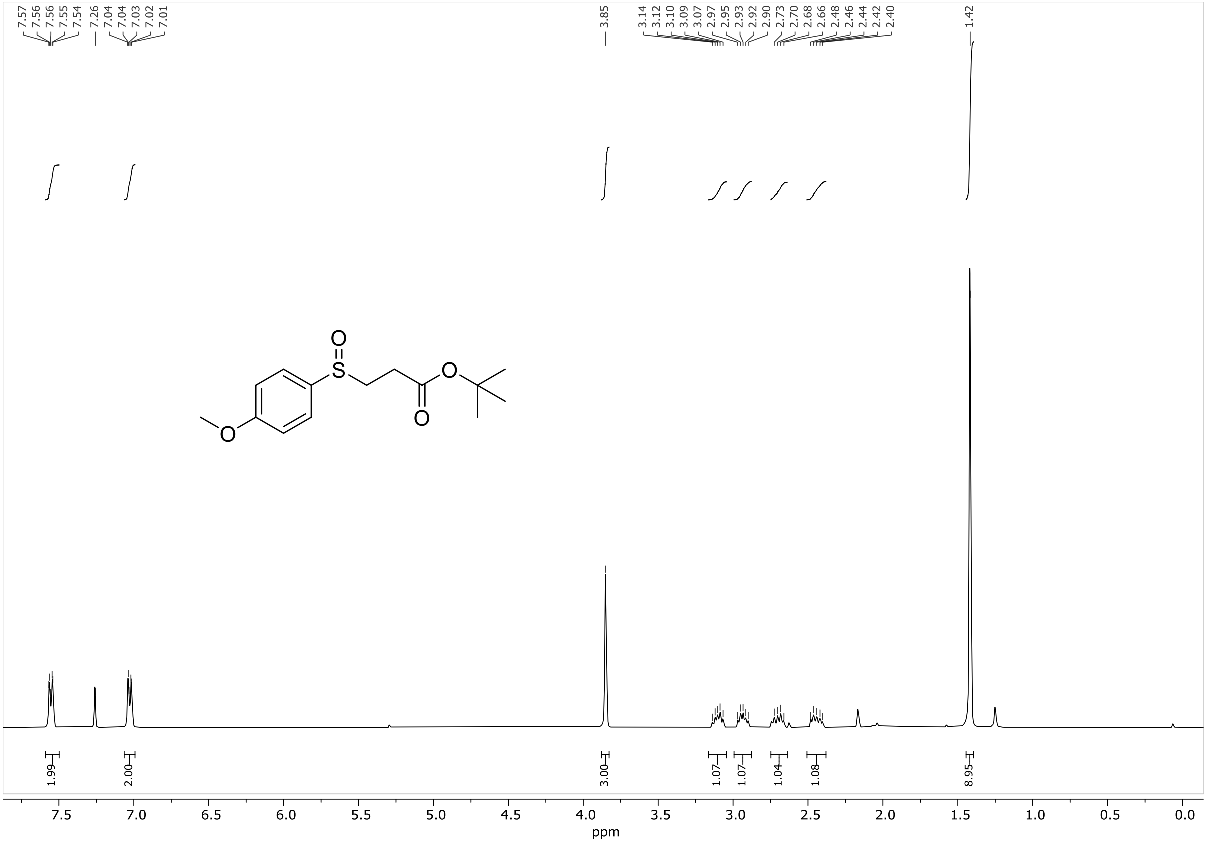

Figure S37. *^{1}H NMR spectrum (CDCl₃, 400 MHz) of tert-butyl 3-((4-methoxyphenyl)sulfinyl)propanoate (**4d**).*

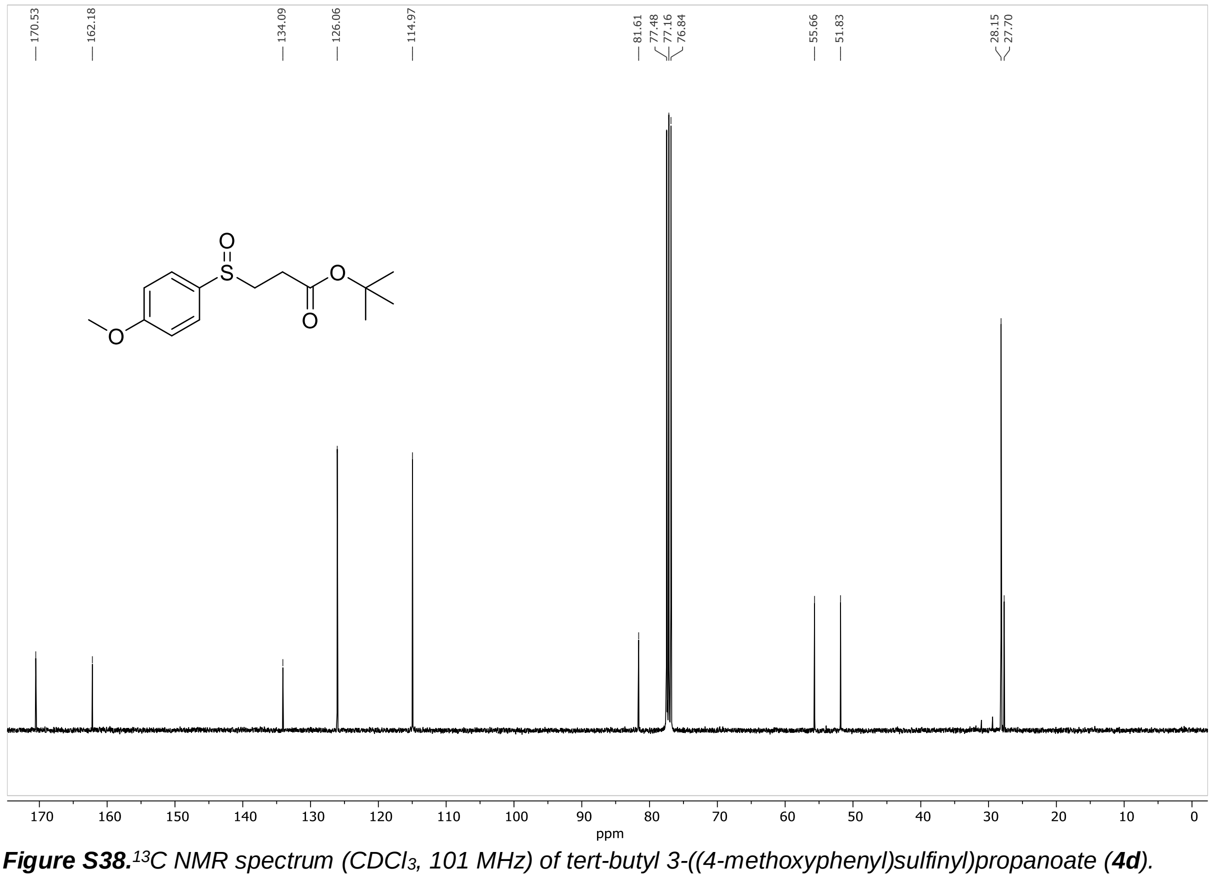

Figure S38. *^{13}C NMR spectrum (CDCl₃, 101 MHz) of tert-butyl 3-((4-methoxyphenyl)sulfinyl)propanoate (**4d**).*

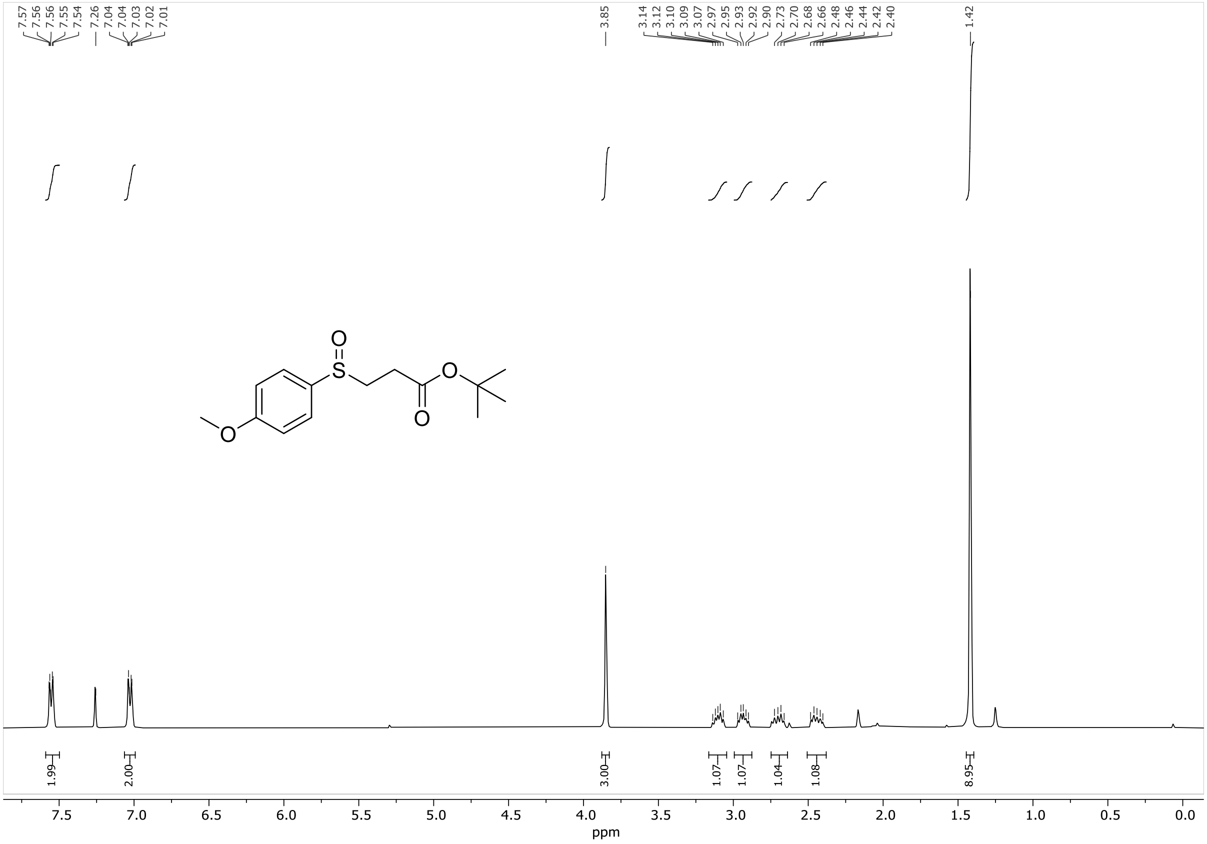

Figure S39.[1]*H NMR spectrum (CDCl$_3$, 400 MHz) of tert-butyl 3-((4-methoxyphenyl)sulfinyl)propanoate (**4d**).*

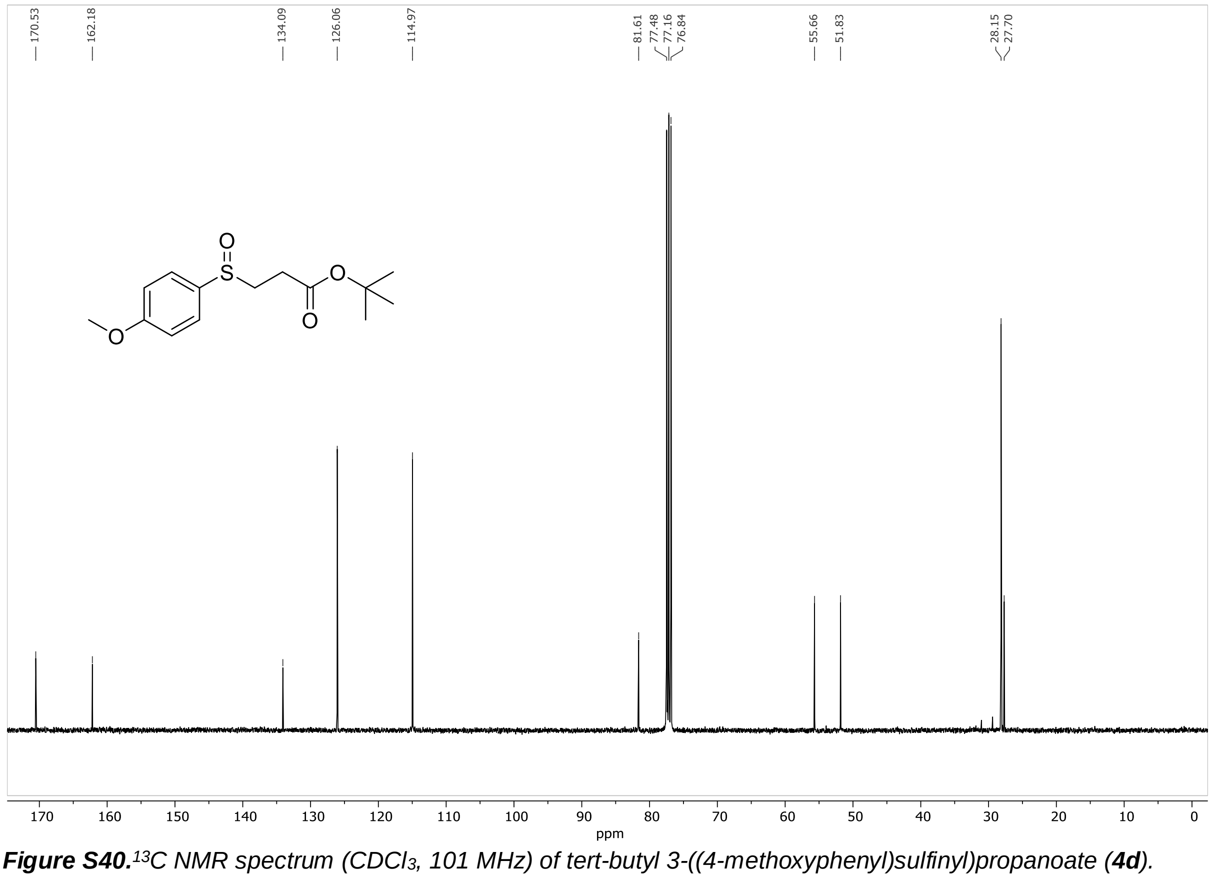

Figure S40.[13]*C NMR spectrum (CDCl$_3$, 101 MHz) of tert-butyl 3-((4-methoxyphenyl)sulfinyl)propanoate (**4d**).*

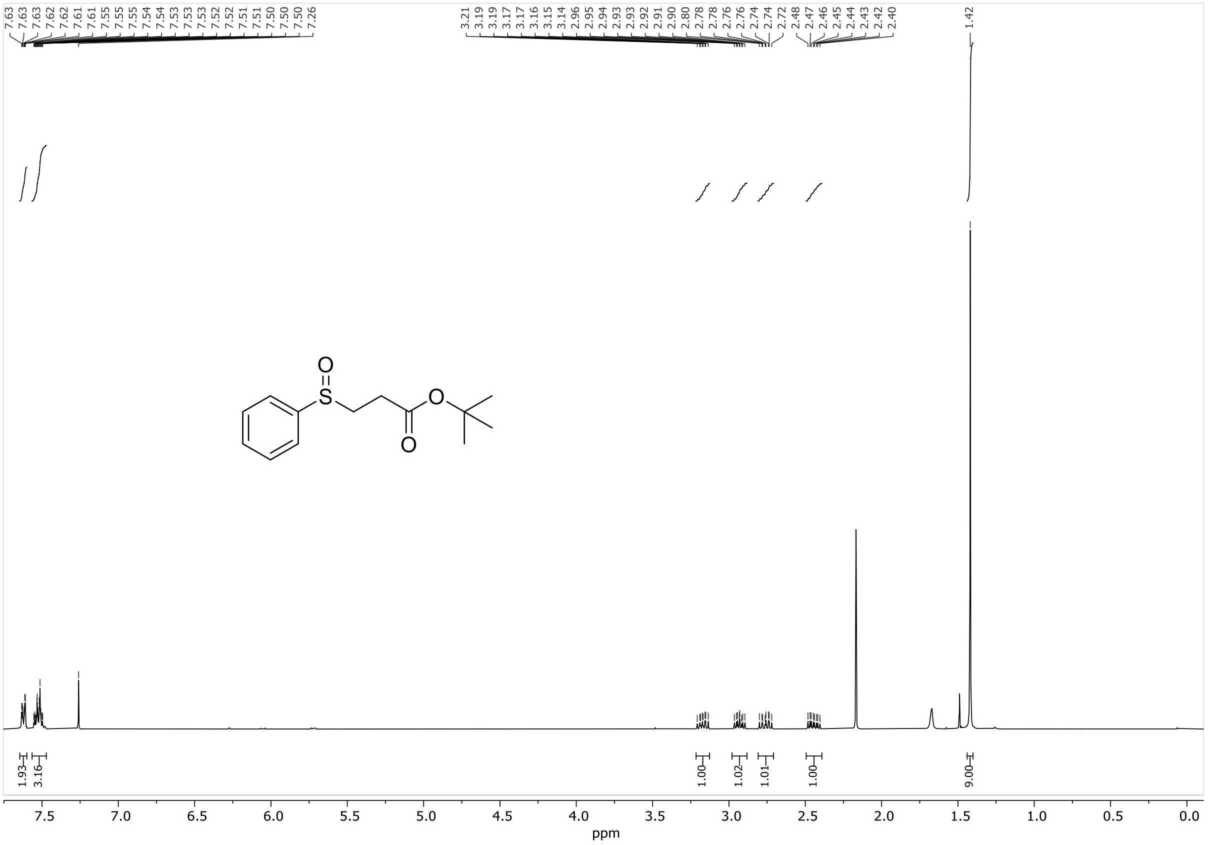

Figure S41.[1]*H NMR spectrum (CDCl*$_3$*, 400 MHz) of tert-butyl 3-(phenylsulfinyl)propanoate (**4e**).*

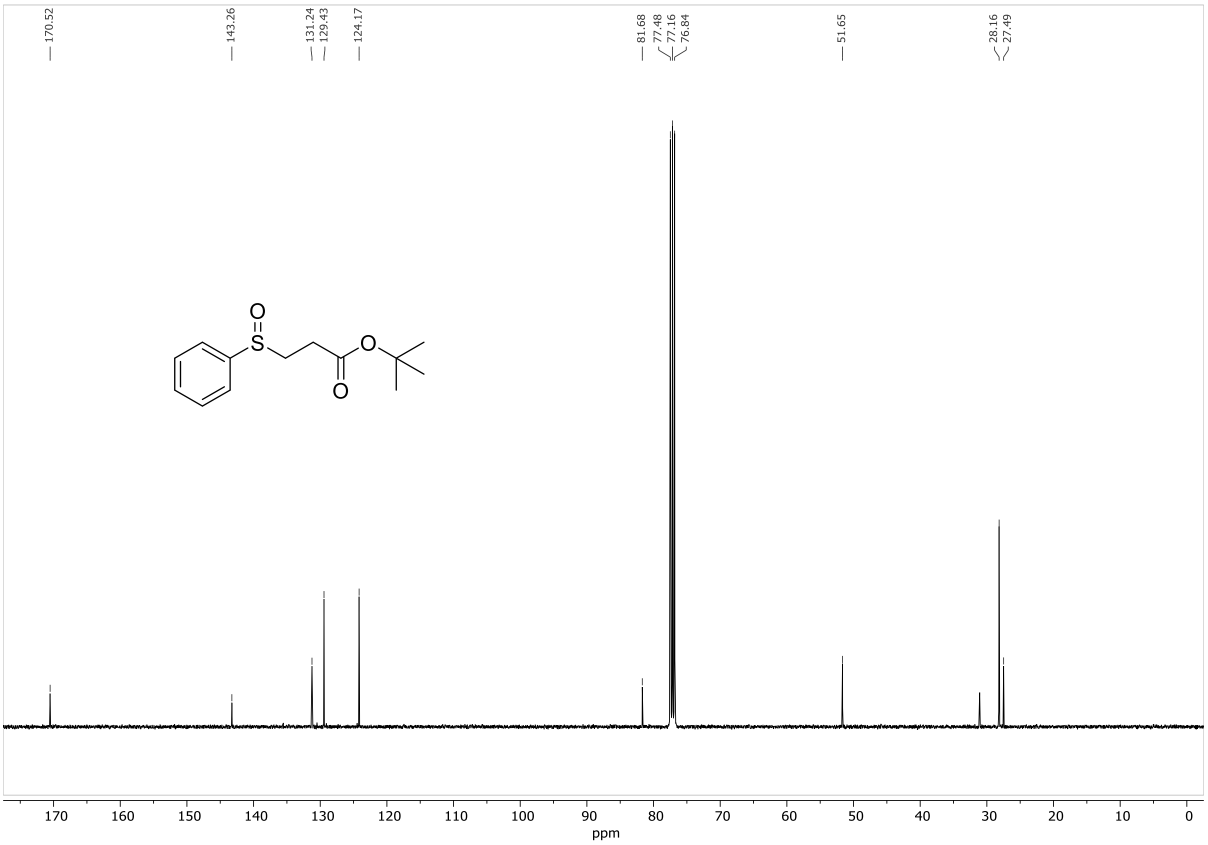

Figure S42.[13]*C NMR spectrum (CDCl*$_3$*, 101 MHz) of tert-butyl 3-(phenylsulfinyl)propanoate (**4e**).*

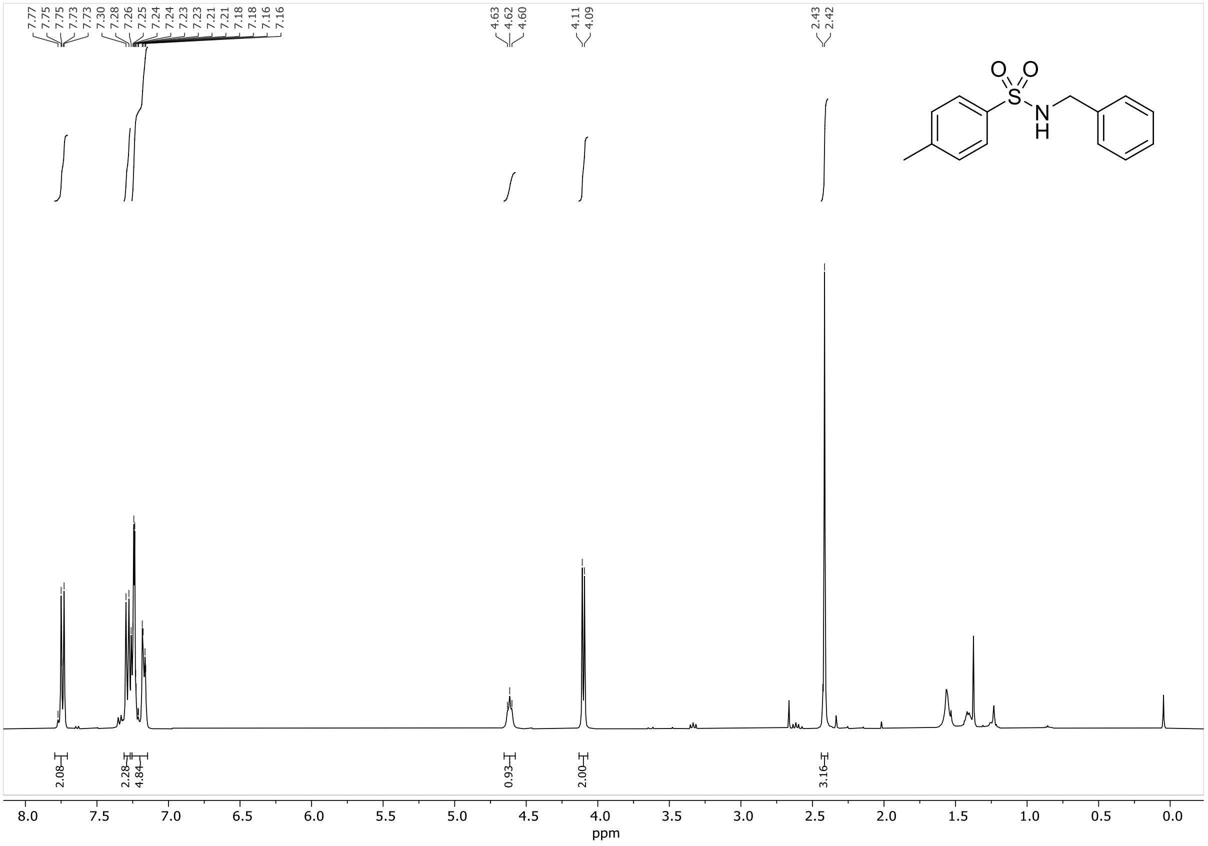

Figure S43.[1]*H NMR spectrum (CDCl$_3$, 400 MHz) of N-benzyl-p-toluenesulfonamide (**5aa**).*

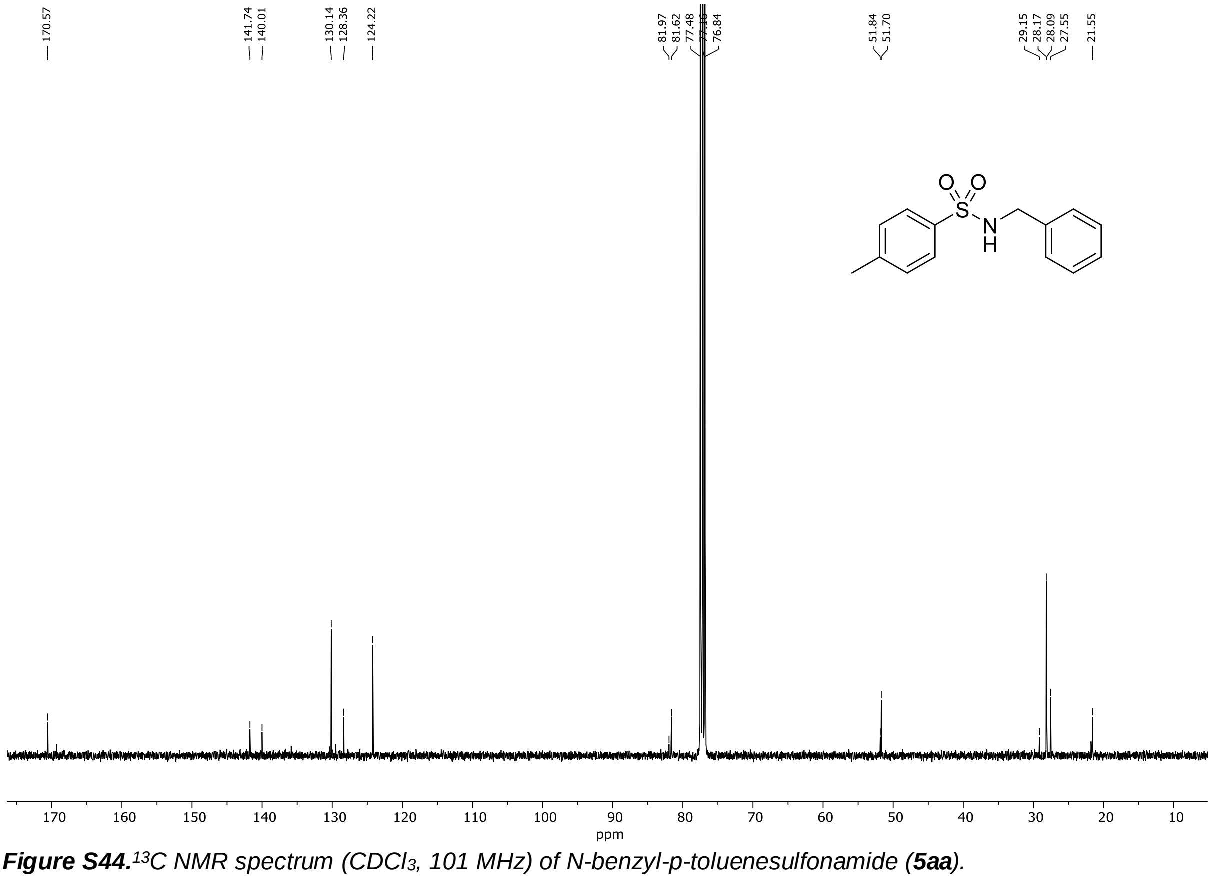

Figure S44.[13]*C NMR spectrum (CDCl$_3$, 101 MHz) of N-benzyl-p-toluenesulfonamide (**5aa**).*

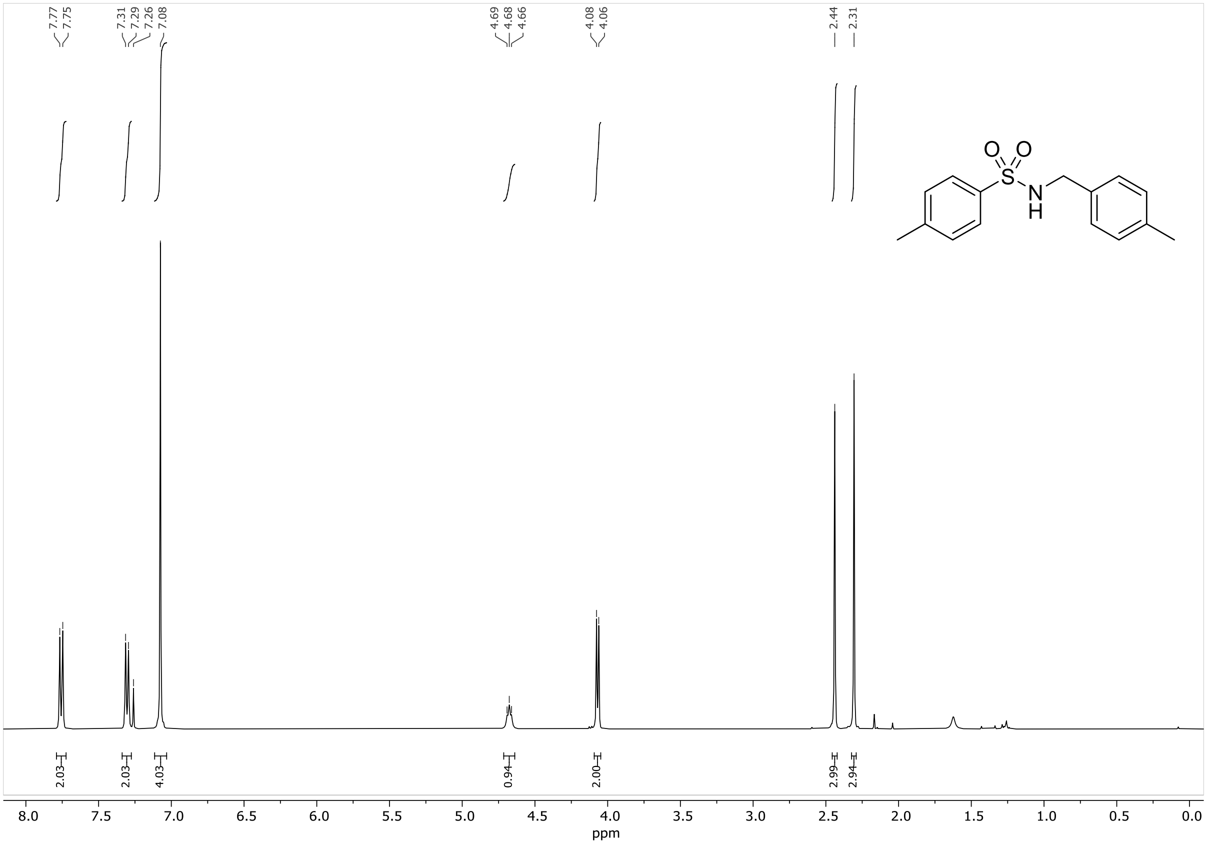

Figure S45.[1]*H NMR spectrum ($CDCl_3$, 400 MHz) of N-(4-methylbenzyl)-p-toluenesulfonamide (**5ab**).*

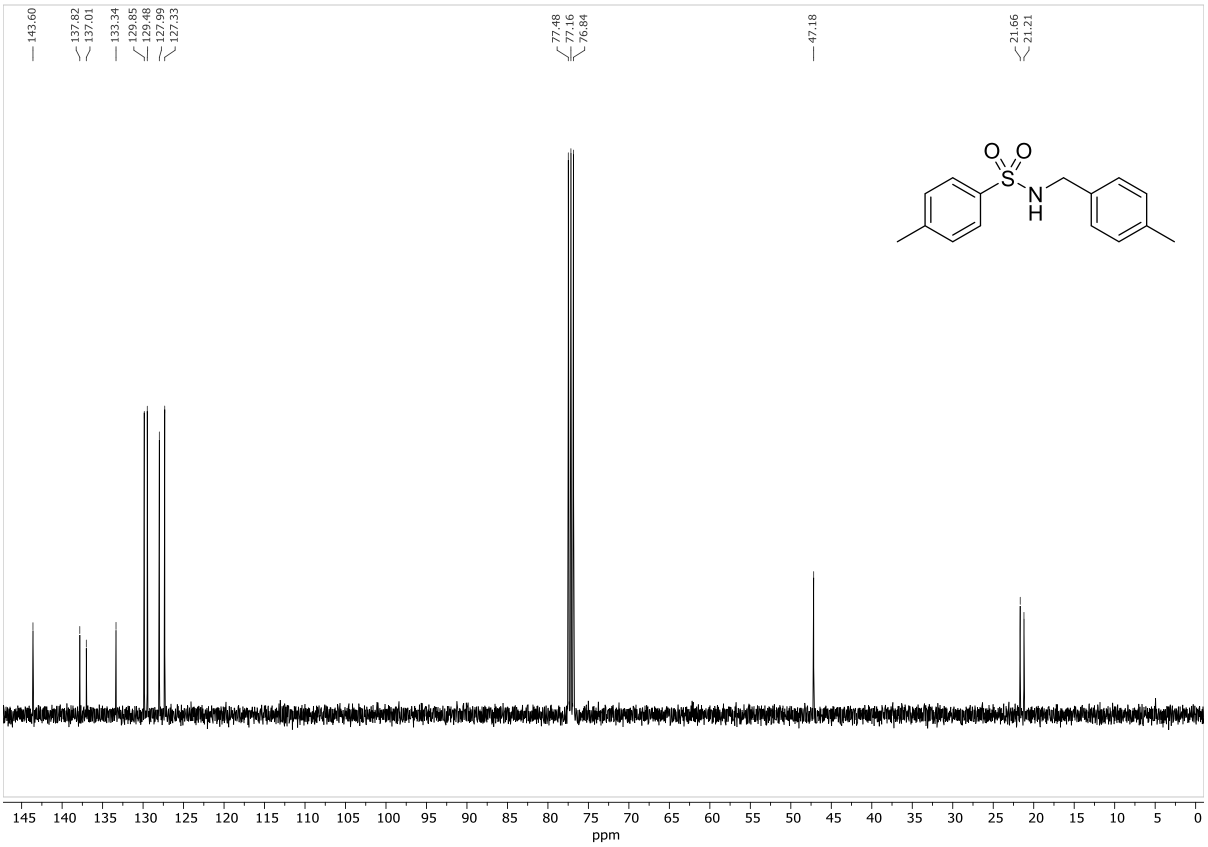

Figure S46.[13]*C NMR spectrum ($CDCl_3$, 101 MHz) of N-(4-methylbenzyl)-p-toluenesulfonamide (**5ab**).*

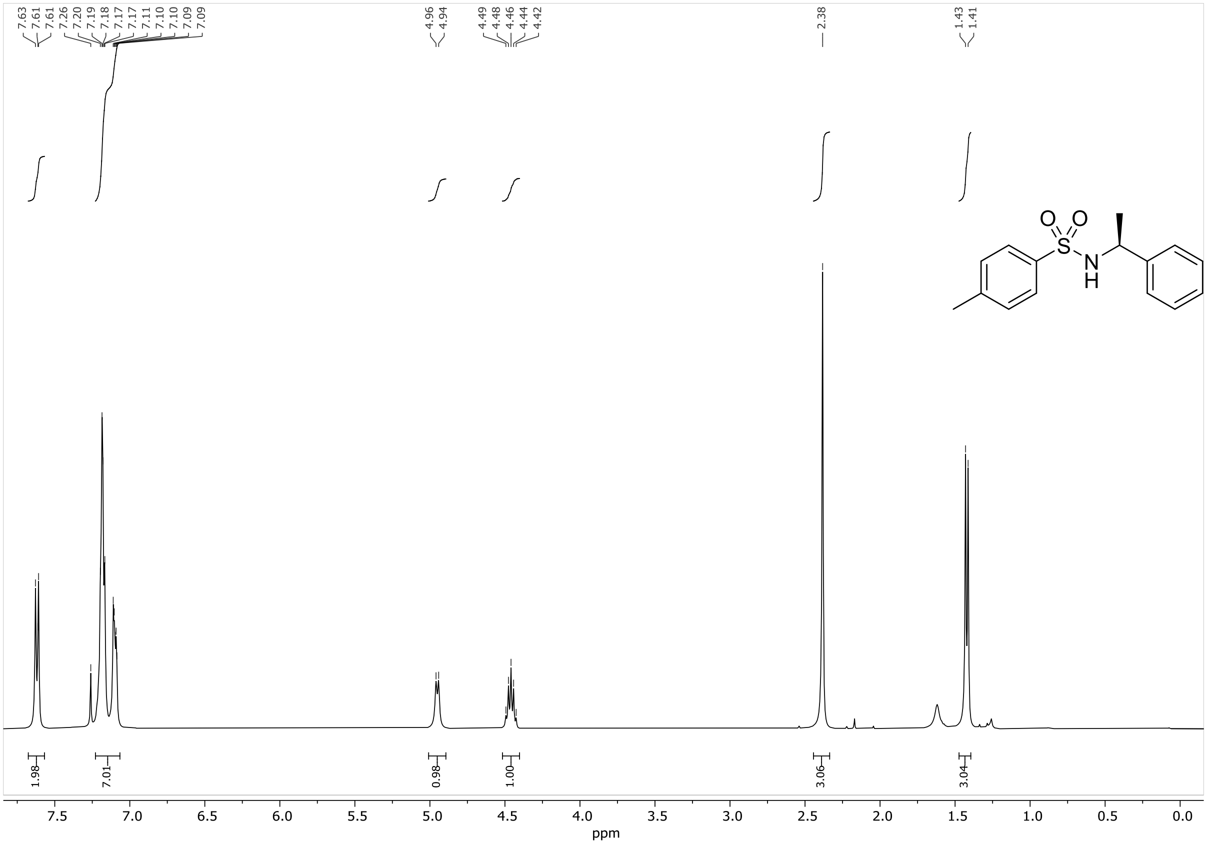

Figure S47. *^{1}H NMR spectrum ($CDCl_3$, 400 MHz) of (S)-4-methyl-N-(1-phenylethyl)benzenesulfonamide (**5ac**).*

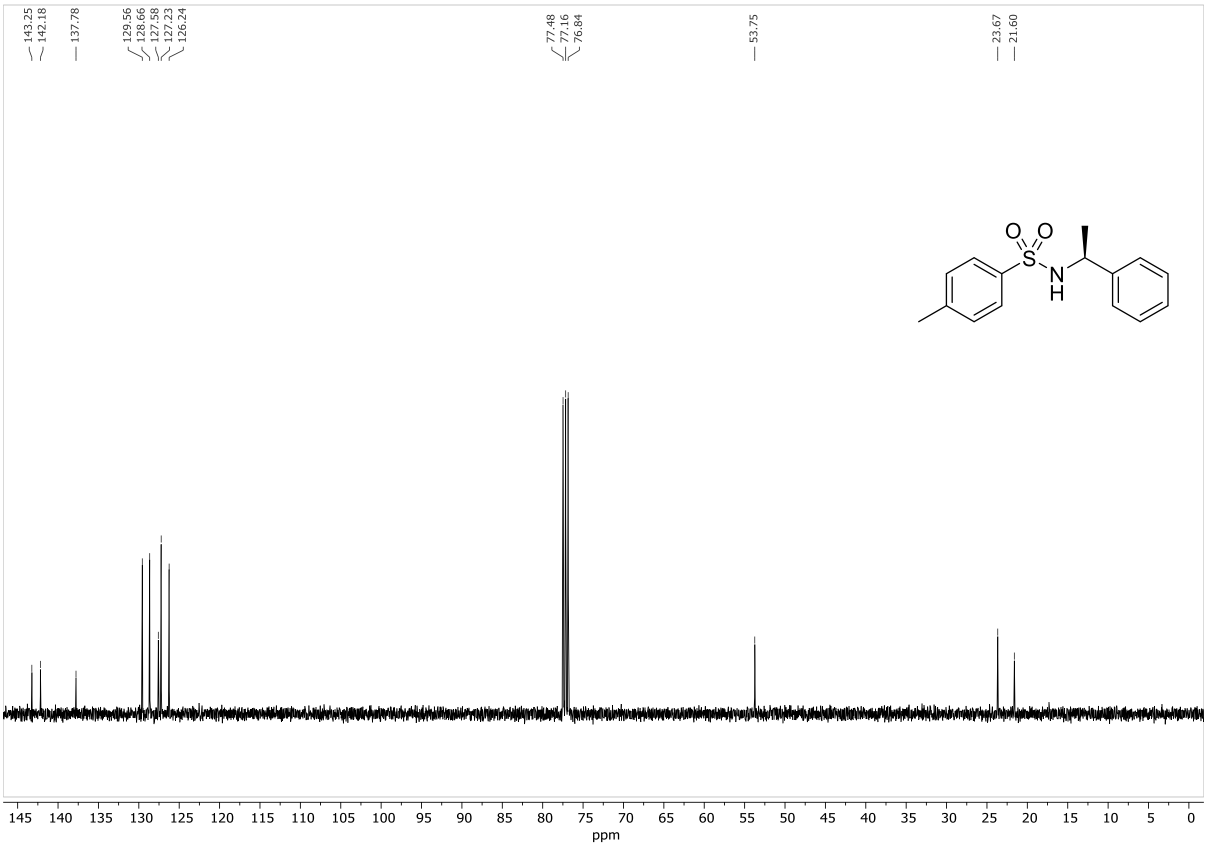

Figure S48. *^{13}C NMR spectrum ($CDCl_3$, 101 MHz) of (S)-4-methyl-N-(1-phenylethyl)benzenesulfonamide (**5ac**).*

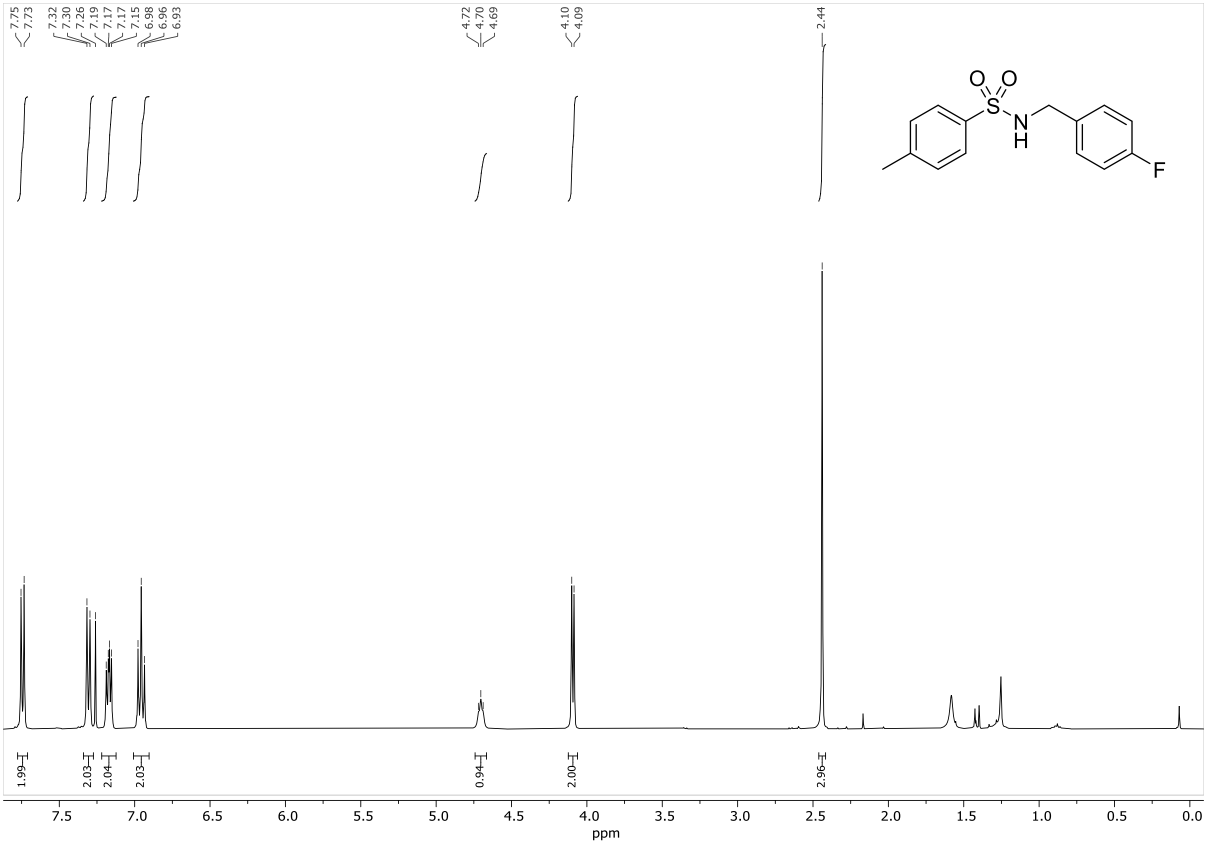

Figure S49.[1]*H NMR spectrum (CDCl$_3$, 400 MHz) of N-(4-fluorobenzyl)-p-toluenesulfonamide (**5ad**).*

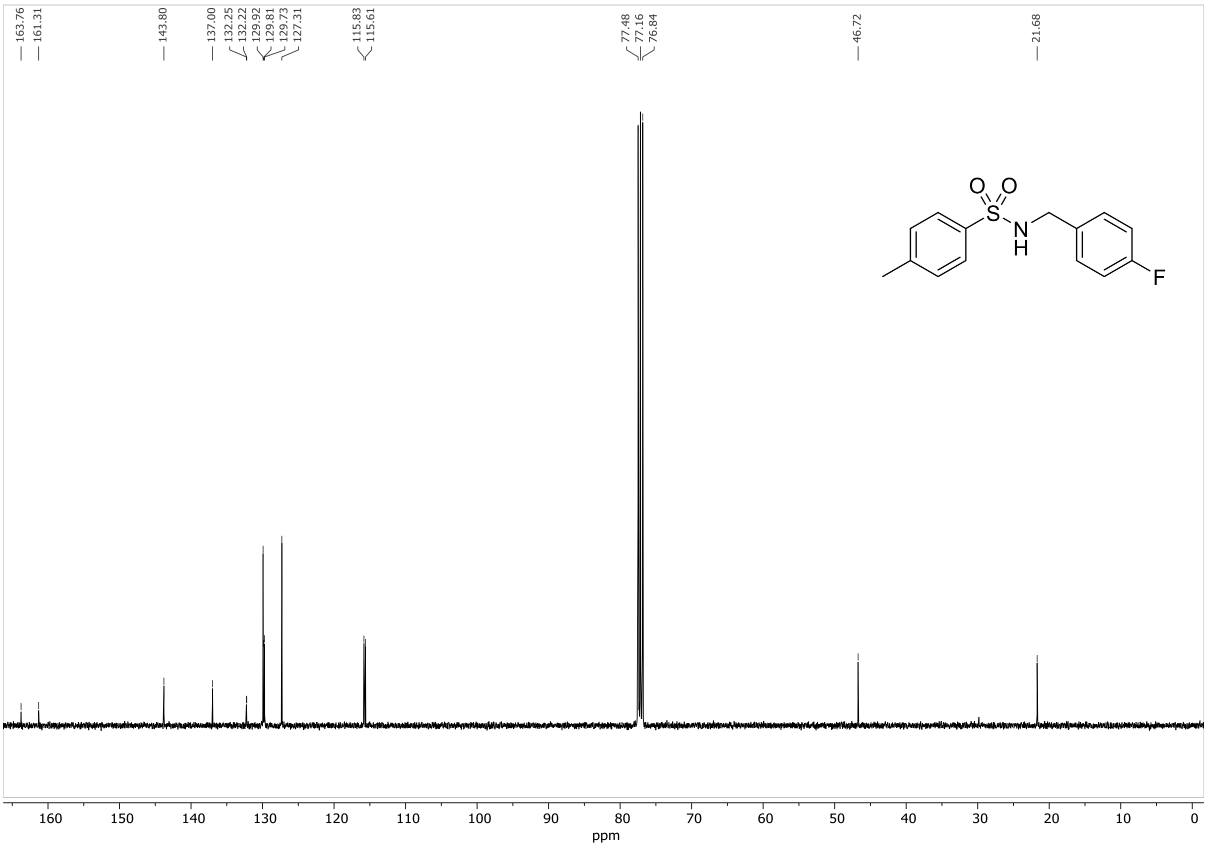

Figure S50.[13]*C NMR spectrum (CDCl$_3$, 101 MHz) of N-(4-fluorobenzyl)-p-toluenesulfonamide (**5ad**).*

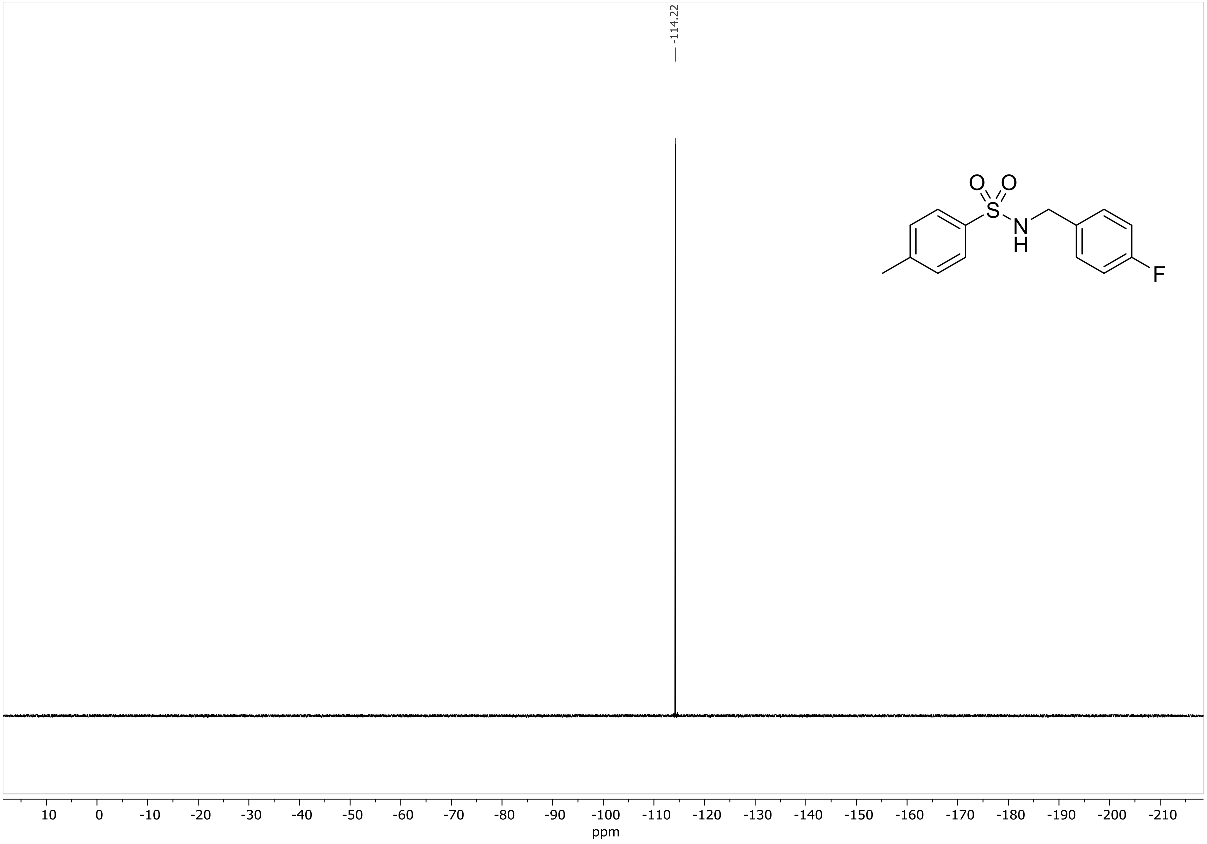

Figure S51.[19]*F NMR spectrum (CDCl$_3$, 376 MHz) of N-(4-fluorobenzyl)-p-toluenesulfonamide (**5ad**).*

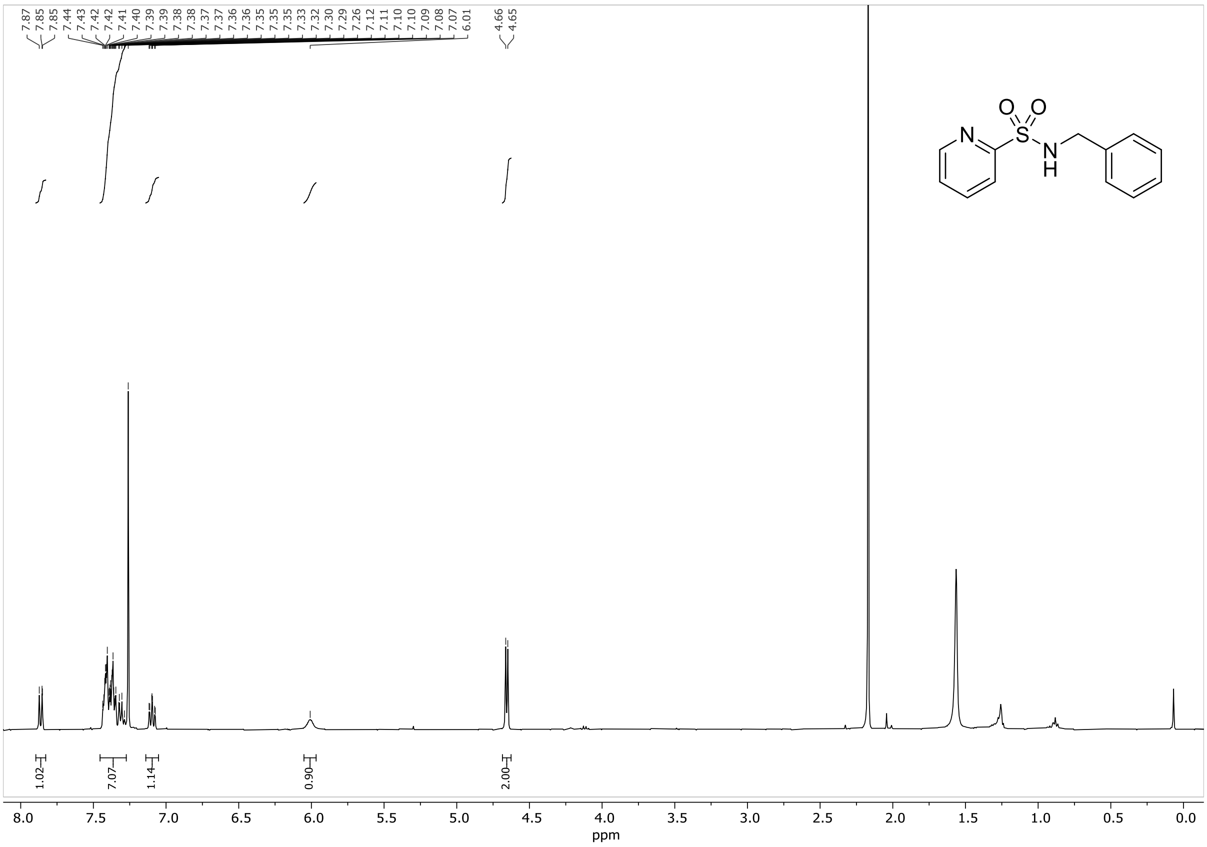

Figure S52.[1]*H NMR spectrum (CDCl$_3$, 400 MHz) of N-benzylpyridine-2-sulfonamide (**5ba**).*

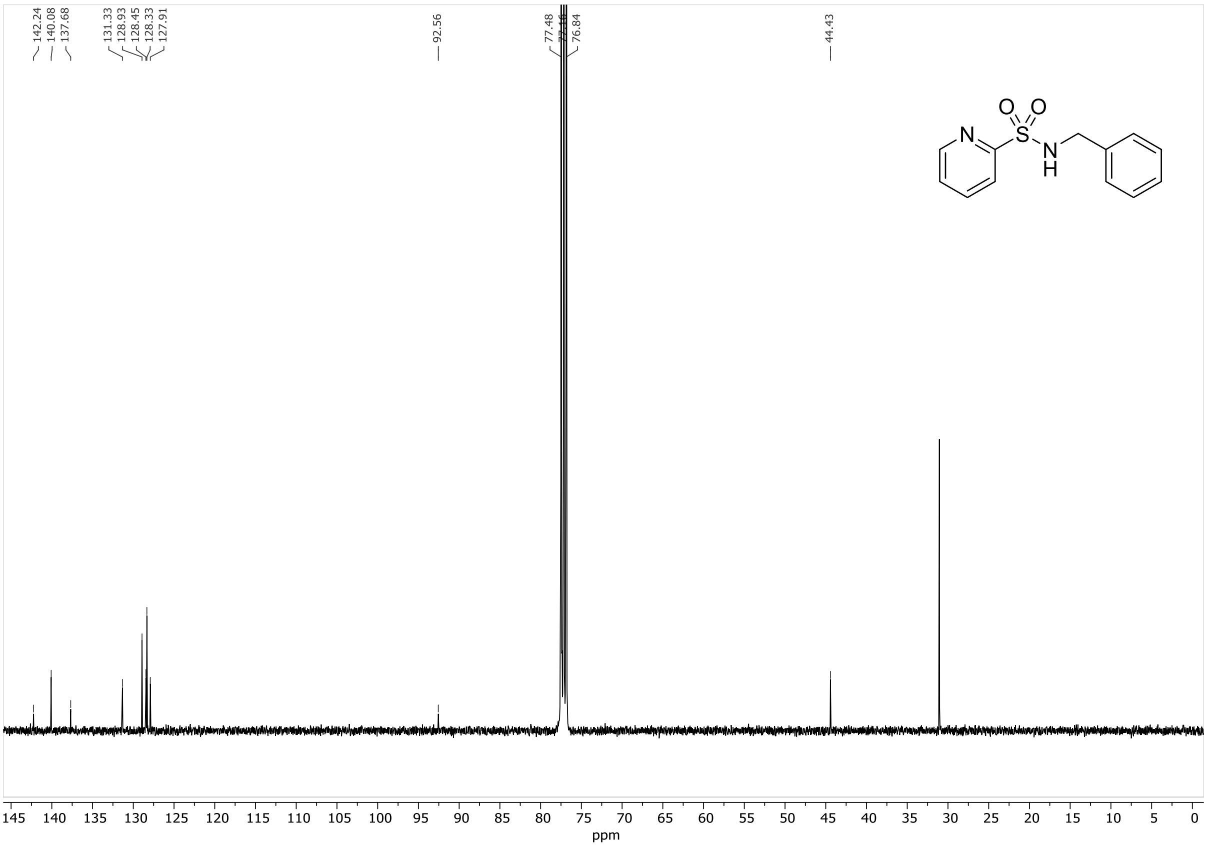

Figure S53.^{13}C NMR spectrum ($CDCl_3$, 101 MHz) of N-benzylpyridine-2-sulfonamide (**5ba**).

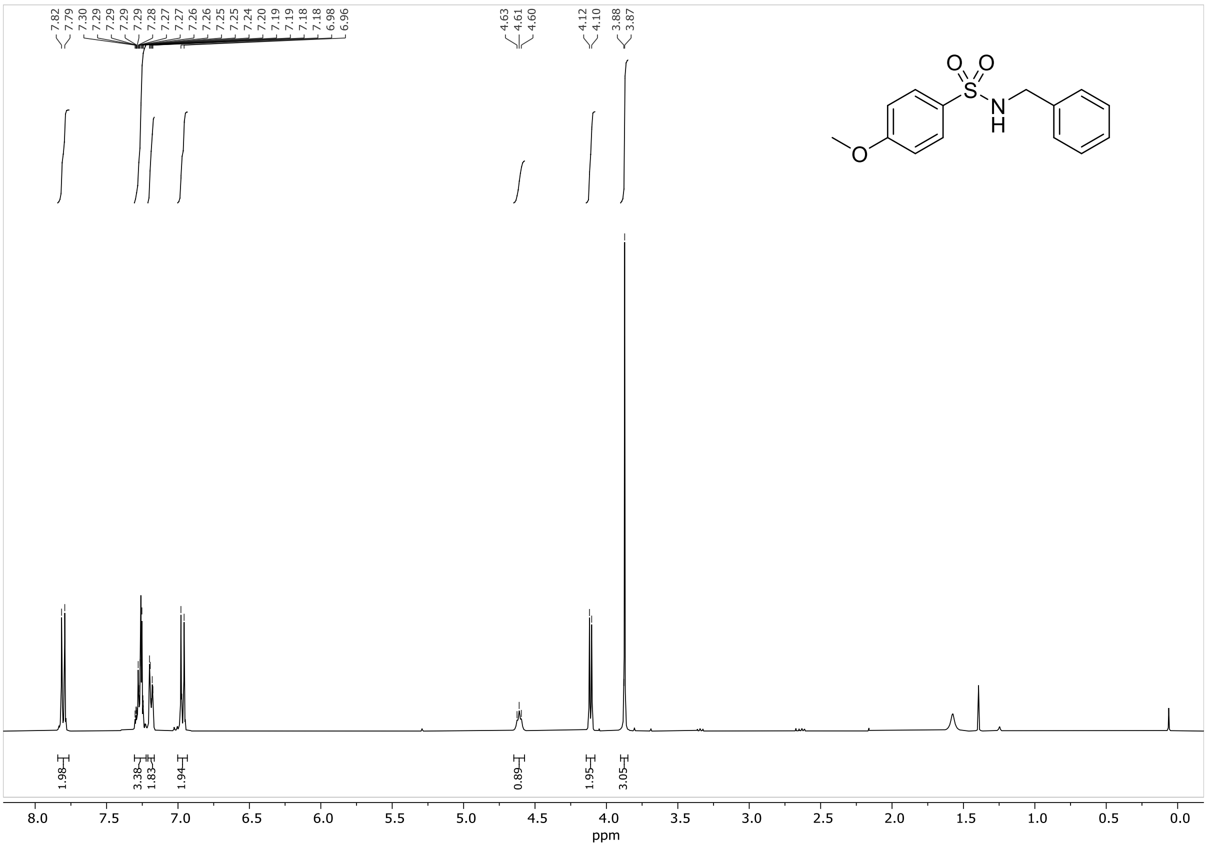

Figure S54.^{1}H NMR spectrum ($CDCl_3$, 400 MHz) of N-benzyl-4-methoxybenzenesulfonamide (**5da**).

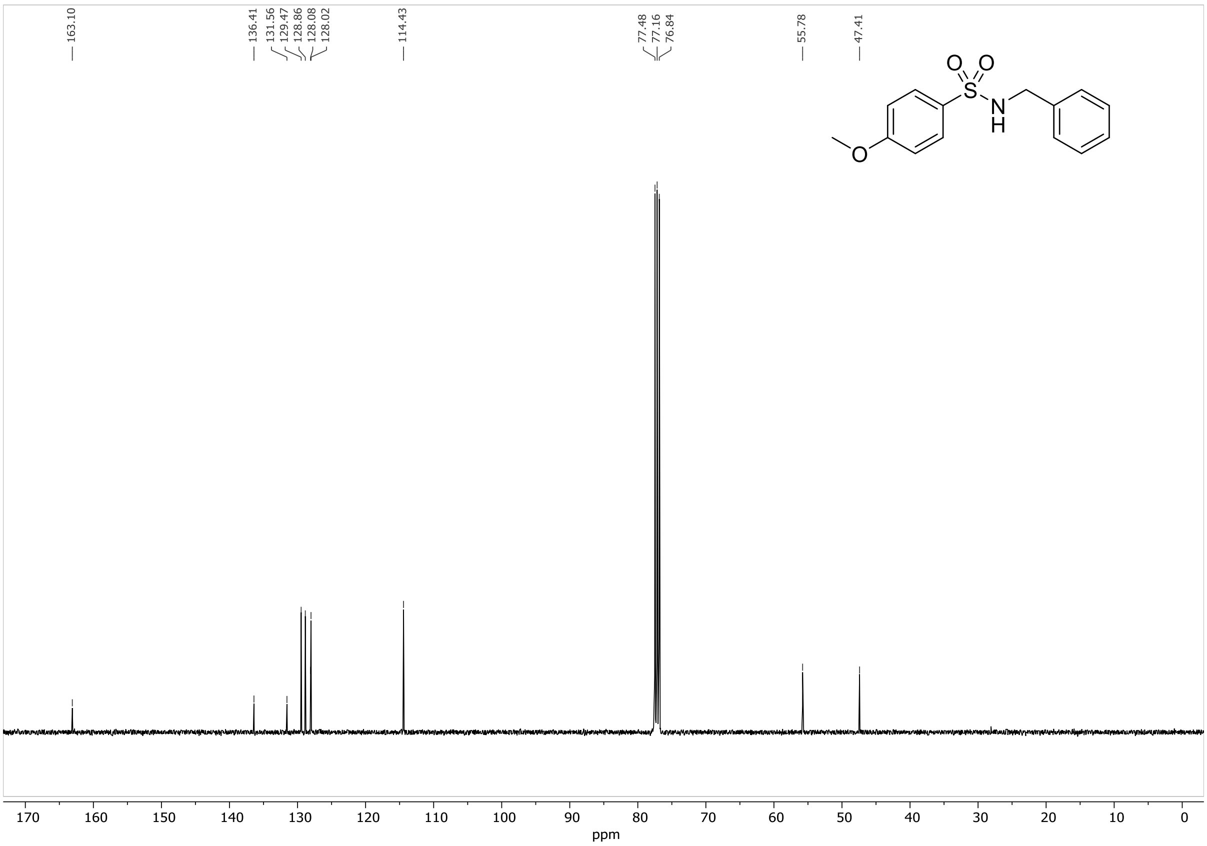

Figure S55.^{13}C NMR spectrum (CDCl$_3$, 101 MHz) of N-benzyl-4-methoxybenzenesulfonamide (**5da**).

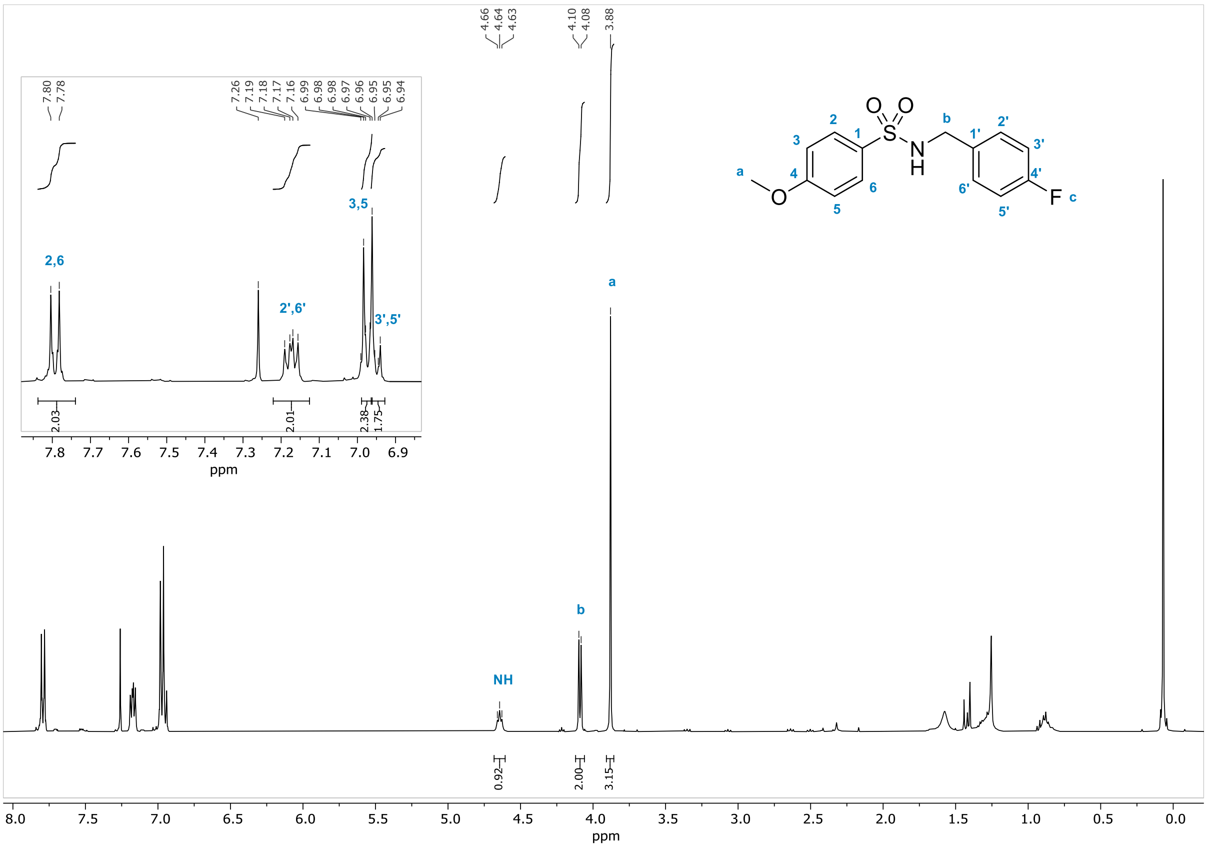

Figure S56.^{1}H NMR spectrum (CDCl$_3$, 400 MHz) of N-(4-fluorobenzyl)-4-methoxybenzenesulfonamide (**5dd**).

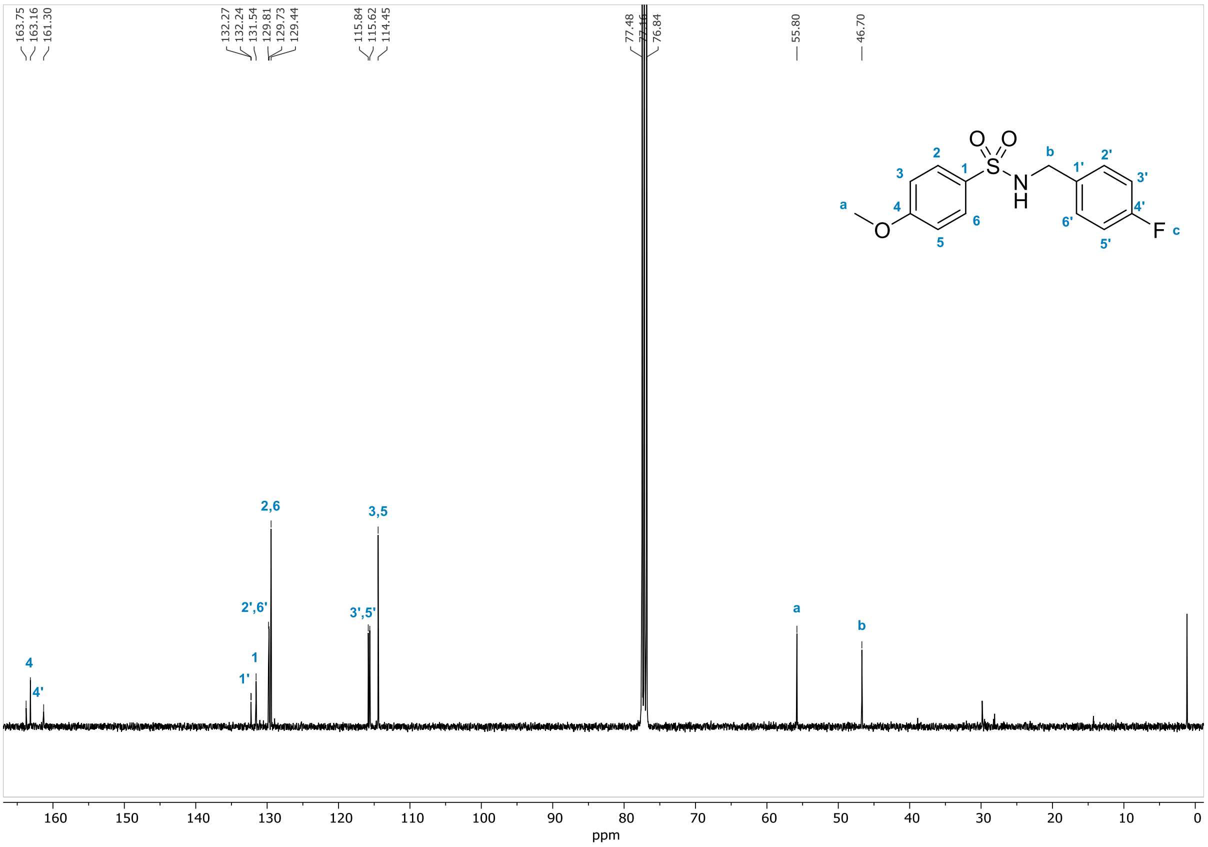

Figure S57. *^{13}C NMR spectrum (CDCl$_3$, 101 MHz) of N-(4-fluorobenzyl)-4-methoxybenzenesulfonamide (**5dd**).*

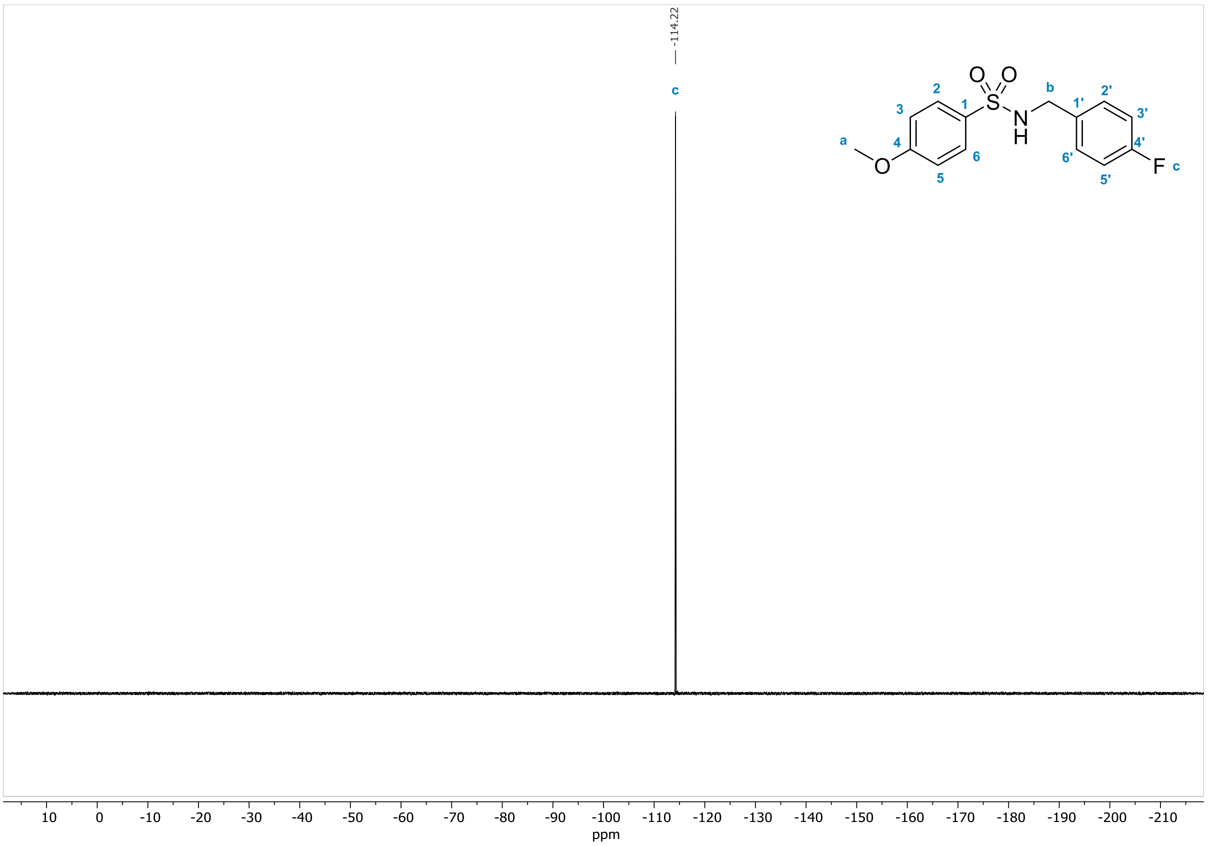

Figure S58. *^{19}F NMR spectrum (CDCl$_3$, 376 MHz) of N-(4-fluorobenzyl)-4-methoxybenzenesulfonamide (**5dd**).*

Table S2. *^{1}H, ^{13}C and ^{19}F NMRs assignment of N-(4-fluorobenzyl)-4-methoxybenzenesulfonamide (**5dd**).*

	^{1}H NMR (ppm)	^{13}C NMR (ppm)	^{19}F NMR (ppm)
1		131.54	
2, 6	7.79 (d, *J* = 8.9 Hz)	129.44	
3, 5	6.99 – 6.96 (m)	114.45	
4		163.16	
1'		132.25 (d, *J* = 3.3 Hz)	
2', 6'	7.17 (dd, *J* = 8.5, 5.4 Hz)	129.77 (d, *J* = 8.3 Hz)	
3', 5'	6.96 – 6.93 (m)	115.73 (d, *J* = 21.6 Hz)	
4'		162.53 (d, *J* = 246.7 Hz)	
a	3.88 (s, 3H)	55.80	
b	4.09 (d, *J* = 6.3 Hz)	46.70	
c			-114.22
NH	4.64 (t, *J* = 6.2 Hz)		

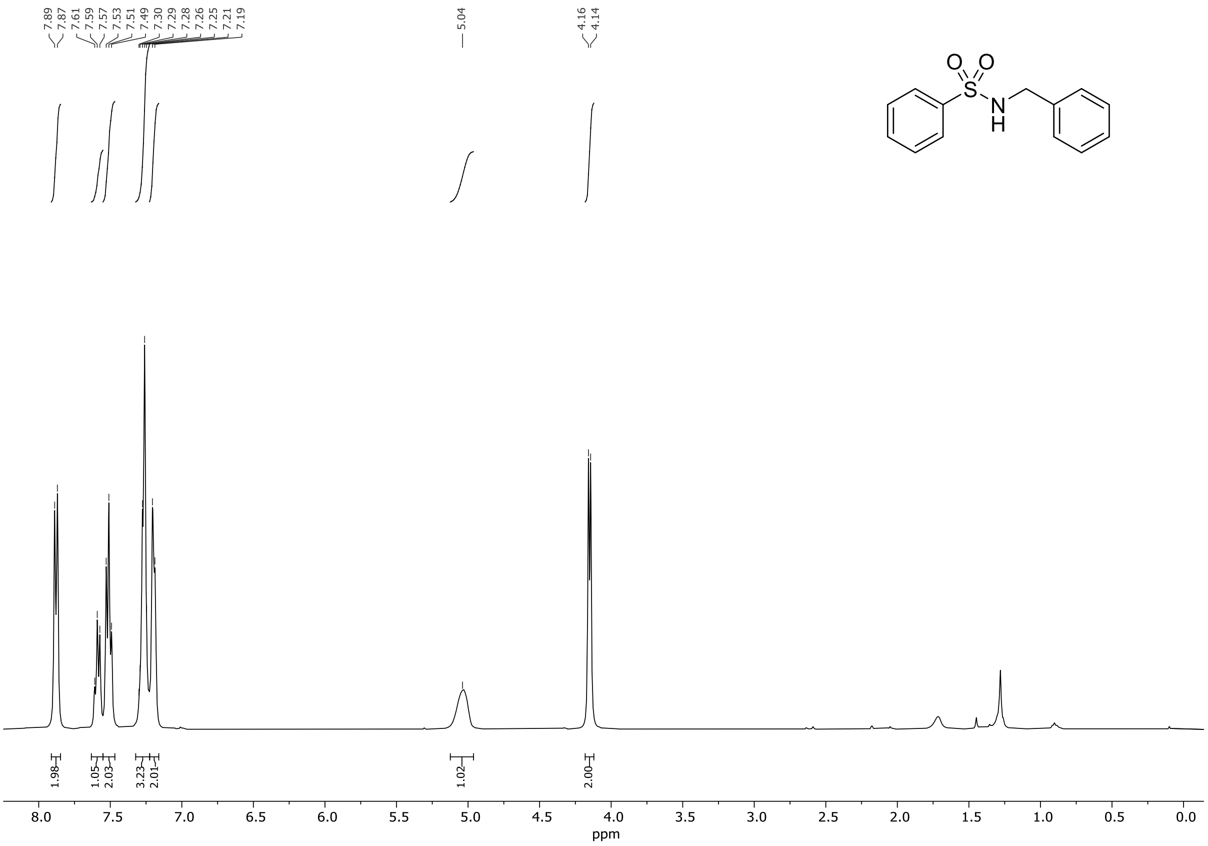

Figure S59. *^{1}H NMR spectrum ($CDCl_3$, 400 MHz) of N-benzylbenzenesulfonamide (**5ea**).*

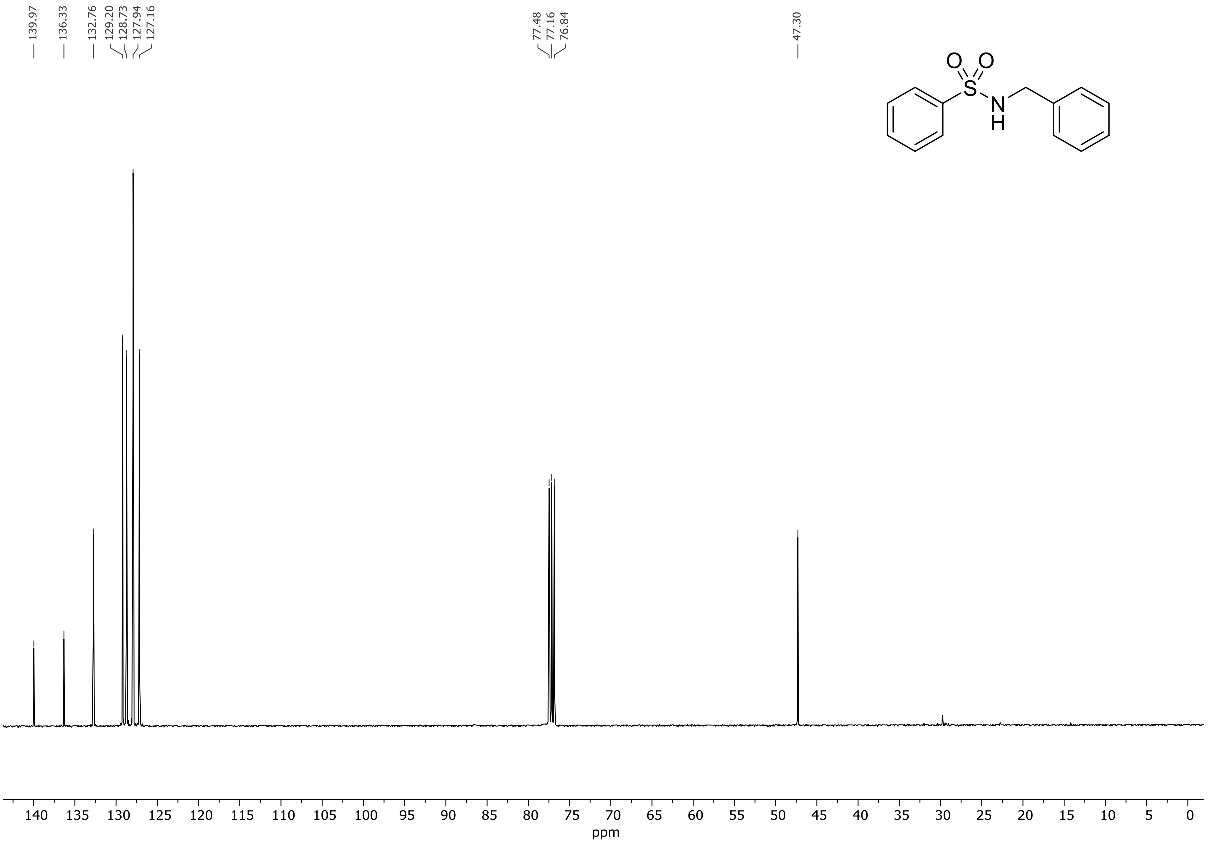

Figure S60. *^{13}C NMR spectrum ($CDCl_3$, 101 MHz) of N-benzylbenzenesulfonamide (**5ea**).*
